# Supplementary material for: Adipose derived stromal vascular fraction and fat graft for treating the hands of patients with systemic sclerosis. A randomized clinical trial
Source: PLoS One. 2023 Aug 14;18(8):e0289594. doi: 10.1371/journal.pone.0289594 (PMC10424873; doi:10.1371/journal.pone.0289594)
Supplement: S1 File — (DOCX) [file pone.0289594.s002.docx]

|  | Comité de Ética en Investigación/Comité de Investigación | Código: |
| --- | --- | --- |
|  |  | Rev. 1 |
|  | **Solicitud de evaluación de protocolos de investigación** | Hoja: 1 de 28 |

No. de registro CIIBH: SCI-1505-15/15-1

| **1. Título del proyecto** |
| --- |
| Seguridad del tratamiento de úlceras digitales isquémicas, refractarias, por esclerosis sistémica con lipo-injerto enriquecido con fracción estromal vascular derivada de tejido adiposo. |
| **2.Número y versión del protocolo (incluya la fecha de la versión)** |
| Número: 1  Versión 1 fecha 29/01/2015 |
| **3.Tipo de investigación** |
| \| **Tipo de investigación** \| **Seleccione una opción** \| \| --- \| --- \| \| Farmacológica \|  \| \| Biomédica \| **x** \| \| Epidemiológica \|  \| \| Intercambiabilidad \|  \| \| Otra \|  \| |
| **4. Investigadores**  **4a. Identificación**   \| **INVESTIGADOR** \| **Posición institucional** \| **Posición en el proyecto** \| **Teléfono (ext.)** \| **Correo-E** \| \| --- \| --- \| --- \| --- \| --- \| \| Dr. Martín Iglesias Morales \| Jefe del Servicio de Cirugía Plástica del Instituto Nacional de Ciencias Médicas y Nutrición “Salvador Zubirán” INCMNSZ \| Investigador principal \| 2140 \| iglesias@drmartiniglesias.com \| \| Dra. Magda Patricia Butrón Gandarillas \| Médico adscrito del Servicio de Cirugía Plástica INCMNSZ \| Investigador Asociado \| 2140 \| butronpaty@yahoo.com.mx \| \| Dr. Francisco Javier Pineda Gutiérrez \| Médico Pasante de Servicio Social en el Servicio de Cirugía Plástica INCMNSZ \| Investigador Asociado \| 2140 \| fjpinedagu@gmail.com \| \| Dr. Armando Roberto Tovar Palacio \| Jefe del Departamento de Fisiología de la Nutrición INCMNSZ \| Investigador Asociado \| 2809 \| tovar.ar@gmail.com \| \| Dra. Tatiana Sofía Rodríguez Reyna \| Investigadora en Ciencias Médicas D. Departamento de Inmunología y Reumatología INCMNSZ \| Investigador Asociado \| 2603 \| sofarodriguez@yahoo.com.mx \| \| Dr. Alejandro Zentella Dehesa \| Jefe del departamento de Bioquímica INCMNSZ \| Investigador Asociado \| 4351 \| azentell@biomedicas.unam.mx \| \| Dr. Iván Torre Villalvazo \| Investigador en Ciencias Médicas C Departamento de Fisiología de la Nutrición INCMNSZ \| Investigador Asociado \|  \| ivan.inn@gmail.com \| \| Dr. Mario Arturo Morán Romero \| Médico Pasante de Servicio Social en el Servicio de Cirugía Plástica INCMNSZ \| Investigador Asociado \|  \| marioarturom@gmail.com \| \| Dr. Ángel Uriel Cruz Reyes \| Médico Pasante de Servicio Social en el Servicio de Cirugía Plástica INCMNSZ \| Investigador Asociado \|  \| drangelcr90@gmail.com \| \| Alan Miguel Hernández Campos \| Estudiante de Medicina, Facultad de Medicina, Universidad Nacional Autónoma de México \| Investigador Asociado \|  \| dr.alan.campos.hdz@gmail.com \| \| Kenia Paulina Zaragoza Cortés \| Estudiante de Medicina, Centro Universitario de Ciencias de la Salud, Universidad de Guadalajara \| Investigador Asociado \|  \| keniia_zc@hotmail.com \| \| Estefanía Alatorre Vázquez \| Estudiante de Medicina, Facultad de Medicina Universidad Autónoma de Sinaloa \| Investigador Asociado \|  \| estefalatorre@gmail.com \| |
| **4b. Pertinencia del grupo de investigadores con respecto del proyecto** |
| \| **Investigador** \| **Pertenencia al SNI** \| **Experiencia en estudios de investigación** \| \| --- \| --- \| --- \| \| Dr. Martin Iglesias Morales \| 1 \| Jefe del Servicio de Cirugía Plástica del INCMNSZ. Cirujano con especialidad de Cirugía Plástica y Reconstructiva; sub especialidad de Microcirugía Reconstructiva y Cirugía de Mano; miembro del Sistema Nacional de Investigadores nivel I; miembro de la Academia Nacional de Medicina; miembro de Academia Mexicana de Cirugía, miembro de International Hand and Composite Tissue Transplantation. Profesor titular del curso de alta especialidad en Cirugía de Mano Reumática de la UNAM en el INCMNSZ, tutor del programa de Investigación del sistema AFINES de la UNAM. Profesor asociado de la Escuela de Medicina del Instituto Tecnológico de Estudios Superiores de Monterrey. \| \| Dra. Magda Patricia Butrón Gandarillas \|  \| Médico Adscrito al Servicio de Cirugía Plástica del INCMNSZ. Cirujano con amplio conocimiento en relación a Cirugía Reconstructiva, Microcirugía y Cirugía de Mano. Miembro del SCOTTCO y de la Asociación Mexicana de Cirugía Plástica Estética y Reconstructiva. \| \| Dr. Armando Roberto Tovar Palacio \| 3 \| Investigador en Ciencias Médicas F. El Dr. Tovar tiene amplia experiencia en análisis y escritura de artículos de investigación básica y participará en el análisis y discusión de resultados y la escritura de los manuscritos. \| \| Dra. Tatiana Sofía Rodríguez Reyna \| 1 \| Investigadora en Ciencias Médicas D. Reumatóloga, encargada de la cohorte de pacientes con Esclerosis Sistémica del INCMNSZ, que actualmente está constituida por 260 pacientes con seguimiento anual. La Dra. Rodríguez tiene formación y experiencia en la atención a pacientes con esclerosis sistémica y sus complicaciones. \| \| Dr. Alejandro Zentella Dehesa \| 2 \| Jefe del departamento de Bioquímica del INCMNSZ. \| \| Dr. Iván Torre Villalvazo \|  \| Investigador en Ciencias Médicas C. El Dr. Torre tiene experiencia en obtención y cultivo primario de células y participará en el procedimiento de aislamiento de células precursoras del estroma vascular de los pacientes, realizará el análisis de expresión de genes por PCR en tiempo real y participará en la discusión de resultados y la escritura de los manuscritos. \| |
| **5. Instituciones participantes** |
| \| **Institución**  **(Razón social y dirección)** \| **Papel que cumplirá en el proyecto** \| **Otorgó aprobación al proyecto?** \| \| --- \| --- \| --- \| \| Instituto Nacional de Ciencias Medica y Nutrición “Salvador Zubirán” Vasco de Quiroga 15, Colonia Sección XVI, Tlalpan C.P.14000, México D.F., MEXICO \| Única institución donde se realizará el estudio. \|  \| |
| **6. Patrocinio**  **6a. Organismos patrocinadores** |
| 1. Departamento de Cirugía del INCMNSZ. Fondos propios del departamento de Cirugía. Proporcionarán los insumos necesarios antes, durante y después del procedimiento de extracción de tejido adiposo y del procedimiento de lipoinjerto enriquecido en células troncales, incluyendo medicamentos y cuidados pre y postquirúrgicos.  2. Departamento de Inmunología y Reumatología del INCMNSZ. Fondos propios destinados a investigación, de la Dra. Tatiana Rodríguez para toma y procesamiento inicial de las muestras de sangre.  3. Departamento de bioquímica del INCMNSZ. Fondos propios del Dr. Alejandro Zentella para reactivos para enriquecimiento del lipoinjerto con células troncales.  4. Departamento de Fisiología de la Nutrición del INCMNSZ. Fondos propios del Dr. Armando Tovar para reactivos para enriquecimiento del lipoinjerto con células troncales. |
| **6b. Especificar si los investigadores reciben pago (monetario o en especie) por su participación específica en la investigación.** |
| Los investigadores no reciben ningún tipo de pago monetario por su participación en esta investigación. |
| **7. Resumen (Límite 400 palabras)** |
| El compromiso de la microcirculación ocasionado por la Esclerosis Sistémica (ES) produce lesiones digitales, caracterizadas por úlceras (UDES), cicatrices puntiformes, necrosis digital y calcinosis; siendo las UDES una complicación frecuente y que causa importante discapacidad y complicaciones como infecciones y amputación del tejido involucrado. Las úlceras suelen ser persistentes, de difícil manejo, extremadamente dolorosas, pueden causar pérdida de tejido y daño a las funciones de la mano. Impactan directamente en la calidad de vida del paciente generando mayor riesgo para el desarrollo de infecciones (gangrena, osteomielitis y septicemia) y amputaciones, periodo prolongado de cicatrización y costo económico elevado. Su tiempo promedio de curación reportado es de 76.2 días (mínimo 7 días, máximo 810 días) realizando el tratamiento a base de curaciones continuas, vasodilatadores, analgésicos y, en caso necesario, antibióticos.  La aplicación de la fracción estromal vascular derivada de tejido adiposo (FEVTA) ha demostrado mayor rapidez en la cicatrización en heridas con compromiso circulatorio. Por lo tanto el objetivo de este protocolo es evaluar la seguridad del trasplante de FEVTA en pacientes con úlceras digitales secundarias a ES. Debido a que no hay información del uso de FEVTA en esta patología, la realización de este estudio piloto nos permitirá obtener mediciones de eficacia de FEVTA en el tiempo de curación de las UD para realizar un estudio posterior con un cálculo de tamaño de muestra enfocado a eficacia.  El estudio será prospectivo, longitudinal, monocéntrico, aleatorizado y no cegado. 10 pacientes con UDES serán grupo control y en 10 pacientes con UDES se les aplicara la FEVTA distribuida en dorso y palma de la mano y dedos. El departamento de Cirugía plástica realizará la extracción de grasa a través de liposucción, la cual posteriormente será procesada en el Departamento de Fisiología de la Nutrición para la extracción FEVTA, para después aplicar la FEVTA más lipoinjerto en las regiones antes descritas.  El seguimiento será a 6 meses y se evaluará: la circulación regional, la mejoría en el dolor con una escala visual análoga y función de la mano con las escalas de COCHIN y SHAQ, la mejoría en la calidad de vida con el cuestionario SF-36 y el efecto inmunomodulador con la detección de las subpoblaciones de las células T y B reguladoras.  Se creará una base de datos con las variables demográficas y de las características clínicas de la enfermedad y de la úlcera, en SPSS versión 18. Se utilizará prueba de U de Mann-Whitney para comparar la media del tiempo de curación de la úlcera índice entre los 2 grupos de tratamiento. Las proporciones se compararán con prueba de Chi cuadrada. |
| **8. Antecedentes** |
| La esclerosis sistémica (ES) es una enfermedad autoinmune que afecta el tejido conectivo, arteriolas y la microcirculación. Se caracteriza por la presencia de microvasculopatía funcional y estructural que resulta en isquemia y, por otro lado, por la aparición de fibrosis cutánea y visceral. La enfermedad vascular inicial se evidencia característicamente por el fenómeno de Raynaud y por la presencia de alteraciones microvasculares observadas en capilaroscopía, incluso años antes de la aparición de otros signos de la enfermedad. ^1-^[^5^](#2et92p0)  El compromiso de la microcirculación manifestado generalmente por el fenómeno de Raynaud en la ES ocasiona lesiones digitales como infartos cutáneos que dejan cicatrices puntiformes, así como úlceras y gangrena en los pulpejos, siendo las úlceras digitales (UDES) una de las complicaciones más frecuentes y refractarias al tratamiento. Las UDES son persistentes, de difícil manejo, extremadamente dolorosas, pueden causar pérdida de tejido, auto amputación y daño a las funciones de la mano. Asimismo impactan directamente en la calidad de vida del paciente generando mayor riesgo para el desarrollo de infecciones (gangrena, osteomielitis y septicemia) y amputaciones. [^1^](#gjdgxs)^,^ [^2^](#30j0zll)^,^ [^3^](#1fob9te)  La etiología de las UDES es multifactorial, pero el vasoespasmo, la vasculopatía, la activación plaquetaria intraluminal, la trombosis los traumas repetitivos, la xerosis y el adelgazamiento cutáneo contribuyen al desarrollo y perpetuación de las mismas. [^2^](#30j0zll), [^4^](#3znysh7)  Las UDES se presentan en alrededor de 30-40% de los pacientes con ES. La localización más común es la punta de los dedos y sobre las prominencias óseas. [^3^](#1fob9te) Las úlceras se observan tanto en pacientes con ES difusa como limitada. [^4^](#3znysh7)  Se considera una úlcera activa aquella cuya evolución es menor a 3 meses, y crónica, si es mayor de 3 meses. El 30% de los pacientes con UDES presentan pérdida de tejido blando y óseo. Hasta 12% de los pacientes requieren hospitalización y cirugía. Del 43-58% desarrollará por lo menos una UDES en algún momento de la enfermedad. En 31.8-71.4% de los casos se transformarán en úlceras crónicas y persistentes. 25% Veinticinco por ciento de los pacientes con UDES se presentan con más de dos úlceras digitales, y entre 14-29% de los casos llegan a progresar a gangrena y auto amputación. [^1^](#gjdgxs)^,^ [^4^](#3znysh7)  El estudio de Amanzi et al mostró las características de 1614 UDES en 100 pacientes en un periodo de 4 años. El promedio de UDES por paciente fue de 15.7. La distribución de las UDES por mano fue de 55% mano derecha y 45% mano izquierda. Las UDES fueron localizadas más frecuentemente en el segundo (24.5%) y tercer (26.6%) dedos. El 52% de las UDES fueron localizadas en la punta del dedo, 30% en la región dorsal de los dedos, 13% en el área ungueal y muy rara vez en la región palmar. El tiempo promedio de curación fue de 76.2 días (mínimo 7 días, máximo 810 días) realizando el tratamiento convencional.[^1^](#gjdgxs)  El impacto en la calidad de vida y la discapacidad de los pacientes con UDES se pueden objetivar a través del Health Assessment Questionaire (HAQ), la escala visual para el dolor, la escala de severidad de la úlcera, la pérdida de la función de la extremidad involucrada y la pérdida de la función de la articulación directamente comprometida por la úlcera. [^4^](#3znysh7)  El manejo actual para el tratamiento y prevención del fenómeno de Raynaud y UDES incluye bloqueadores de los canales de calcio (tratamiento de primera línea), antagonistas de los receptores de angiotensina II, antiagregantes plaquetarios, antagonistas del receptor de endotelina (reducen el número de nuevas lesiones especialmente en pacientes con riesgo de úlceras múltiples, pero no se ha encontrado un efecto en la curación), inhibidores de la 5-fosfodiesterasa (muestran un beneficio significativo en términos de frecuencia, duración y severidad de los ataques), estatinas (muestran eficacia en la reducción de la aparición y disminución en el número total de UDES, mejoría en el fenómeno de Raynaud así como en el daño de la función vascular) y análogos de prostaglandinas. [^2^](#30j0zll) Puede considerarse el uso de anticoagulantes en casos isquemia aguda o cuando se sospecha de una complicación trombótica.  La última opción terapéutica es el manejo quirúrgico. Entre las opciones quirúrgicas están: bypass arterial, reconstrucción de la arteria digital, simpatectomía (periférica y digital), e inyección de toxina botulínica. Estas opciones terapéuticas solo se reservan para pacientes con UDES refractarias al tratamiento médico o en pacientes con dolor intratable.[^2^](#30j0zll) El tratamiento médico-quirúrgico convencional consiste en control de la enfermedad y curaciones seriadas con el objeto de prevenir infección hasta lograr la cicatrización secundaria de la úlcera.  **Células Troncales Derivadas del Tejido Adiposo y Fracción Estromal Vascular de Tejido Adiposo**  Las células troncales son una población celular indiferenciada, tienen la capacidad de autorrenovarse o de diferenciarse a otros tipos celulares. Estas células tienen una importancia esencial para la medicina regenerativa, la cual plantea la posibilidad de reparar o reemplazar células, tejidos y/u órganos enfermos, a través del trasplante de células y tejidos sanos. [^11^](#17dp8vu)  Las células troncales se mantienen durante toda la vida del organismo. Este proceso se lleva a cabo mediante dos mecanismos:   1. Replicación asimétrica obligatoria: con cada división celular una de las células hijas conserva la capacidad de autorrenovación, es decir, siguen siendo células troncales; mientras que la otra célula hija entra en una vía de diferenciación. 2. Diferenciación estocástica: una población de células troncales se mantiene por el equilibrio entre las divisiones de las células troncales que generan dos células hijas troncales con capacidad de autorrenovarse o dos células hijas que entrarán en una vía de diferenciación.   Las células troncales embrionarias (CTE) tienen la mayor capacidad regenerativa, son pluripotenciales y pueden diferenciarse a todos los tejidos corporales. Las células troncales pluripotenciales darán origen a las células troncales multipotenciales con capacidad de diferenciación más limitada las cuales a su vez darán origen a las células diferenciadas de las tres capas embrionarias (ectodermo, mesodermo y endodermo). [^12^](#3rdcrjn)^,^ [^11^](#17dp8vu)  Células troncales del adulto como las células troncales mesenquimales (CTM), fueron descritas por Friedenstein et al. en la década de 1960, [^13^](#26in1rg)^,^ [^14^](#lnxbz9) residen en lugares con un microambiente especial el cual recibe el nombre de nicho. Las células de los nichos generan estímulos que regulan la autorrenovación de las células troncales y la generación de células descendientes. [^12^](#3rdcrjn) Las CTM pueden dar origen a distintos tejidos como cartílago, hueso, piel, médula ósea, tejido adiposo, músculo, células epiteliales y células neuronales. Dentro de este grupo de células se encuentran las células estromales de médula ósea (CEMO), las células troncales derivadas de tejido adiposo (CTDTA) o células estromales derivadas del tejido adiposo nombradas de esta manera por la Sociedad Internacional Aplicada al Tejido Graso [^13^](#26in1rg) y las células estromales de la piel. Todas estas células son multipotenciales y están restringidas a diferenciarse en las líneas celulares de los lugares donde residen. [^11^](#17dp8vu)^,^ [^15^](#35nkun2)^,^ [^16^](#1ksv4uv)  De acuerdo con la Sociedad Internacional de Terapia Celular los criterios mínimos para definir a una célula como CTM es la expresión de los siguientes marcadores en su membrana celular: CD73, CD90 y CD105 con ausencia de los siguientes marcadores membranales: CD34, CD45, CD14 o CD11b, CD79α o CD19 y HLA-DR y tener la capacidad de diferenciación a células especializadas derivadas del mesodermo *in vitro*. [^13^](#26in1rg) Sin embargo, en la revisión realizada por Patricia Zuk las células CTDTA son positivas para CD13, CD29, CD44, CD49, CD90 y CD105; y negativas para CD14, CD31, CD45 y CD144. [^17^](#44sinio) ([Tabla 1](#1opuj5n))  Las células troncales del adulto se dividen muy lento en la mayor parte de los tejidos, estas células generan otras que se dividen con rapidez (células amplificadoras en tránsito) y dan origen a células progenitoras las cuales, a su vez, darán origen a las células diferenciadas que formarán parte de las células residentes del órgano donde se llevó a cabo la diferenciación.  Algunos de los lugares donde se ha estudiado la presencia de células troncales del adulto son: médula ósea, hígado, encéfalo, piel, epitelio intestinal, músculo cardiaco y esquelético, córnea y tejido adiposo.  En la médula ósea existen dos tipos de células troncales, las células troncales hematopoyéticas que dan origen a todas las estirpes celulares de la sangre y las células estromales de la médula ósea (CEMO) que son multipotenciales y pueden dar lugar a osteocitos, condrocitos, osteoblastos, adipocitos, mioblastos y células epiteliales. [^12^](#3rdcrjn)^,^ [^18^](#2jxsxqh)  En el hígado las células troncales se encuentran albergadas en los conductos de Hering/ conducto biliar/, estás células pueden dar origen a unas células precursoras (células ovales) que son bipotenciales y pueden diferenciarse a células biliares o a hepatocitos. [^12^](#3rdcrjn)  En el encéfalo residen las células troncales neurales que dan lugar a neuronas, astrocitos, y oligodendrocitos. Se han descrito en dos localizaciones en los encéfalos de adultos, la zona subventricular y la circunvolución dentada del hipocampo. [^12^](#3rdcrjn)^,^ [^19^](#z337ya)  En la piel se encuentran células troncales en tres regiones de la epidermis, en la protrusión del folículo piloso, en las regiones interfoliculares (células basales) y en las glándulas sebáceas. Estás células dan lugar a células de amplificación en tránsito y estas a su vez generan las células de la epidermis diferenciada. [^12^](#3rdcrjn)^,^ [^20^](#3j2qqm3)  En el epitelio intestinal las células troncales se encuentran por encima de las células de Paneth en el intestino delgado y en la base de la cripta de Lieberkühn en el colon. Las células dan origen al epitelio intestinal. [^12^](#3rdcrjn)^,^ [^21^](#1y810tw)  En el músculo cardiaco y esquelético la regeneración celular de los miocitos se da por la replicación de las células satélite, localizadas principalmente en la periferia del sarcolema del miocito. [^12^](#3rdcrjn)  En la córnea, las troncales del limbo se localizan en el limbo esclerocorneal o unión esclerocorneal, estás células mantienen a las células del epitelio anterior de la córnea y con esto ayudan al mantenimiento de la transparencia de la córnea, esencial para una visión óptima. [^12^](#3rdcrjn)^,^ [^22^](#4i7ojhp)  En el 2001 Zuk y cols.[^23^](#2xcytpi) aislaron y caracterizaron células troncales mesenquimatosas (CTM) del tejido adiposo subcutáneo con el mismo potencial de diferenciación encontrado para las CTMO. Este descubrimiento ha abierto la posibilidad de la obtención de células troncales sin procedimientos de alto riesgo, además de que el tejido adiposo contiene gran cantidad de estas células comparado con otros tejidos adultos (5000-8000 CTDTA/ml). [^13^](#26in1rg)^,^ [^24^](#1ci93xb)^,^ [^25^](#3whwml4) Si al tejido adiposo lo dividiéramos en 2 componentes estos serían: el componente graso (adipocitos) y el estroma (tejido conectivo laxo + fracción estromal vascular del tejido adiposo o FEVTA). Es en este segundo es donde se encuentran las CTDTA. [^26^](#2bn6wsx)  A la fecha una amplia variedad de especialidades ha utilizado terapias con células troncales derivadas de tejido adiposo [^27^](#qsh70q) ([Tabla 2](#48pi1tg)). Se han utilizado para el tratamiento de enfermedad de injerto contra huésped [^65^](#2r0uhxc)^,^[^66^](#1664s55), púrpura trombocitopénica idiopática [^67^](#3q5sasy)^,^ [^68^](#25b2l0r), aplasia pura de células rojas [^69^](#kgcv8k), diabetes mellitus tipo I [^70^](#34g0dwd)^,^ [^71^](#1jlao46), fístula entero-cutánea [^72^](#43ky6rz), fístula perianal compleja [^73^](#2iq8gzs)^,^ [^74^](#xvir7l), fístula traqueo-mediastinal [^75^](#3hv69ve), artritis reumatoide [^76^](#1x0gk37), esclerosis múltiple [^77^](#4h042r0), reconstrucción de maxila [^78^](#2w5ecyt), reconstrucción de cigomático [^79^](#1baon6m), reconstrucción de cráneo [^80^](#3vac5uf), reconstrucción de cabeza femoral [^81^](#2afmg28) y corrección de incontinencia urinaria de esfuerzo post prostatectomía [^82^](#pkwqa1). Los autores han reportado disminución en el tiempo de cicatrización y aumento en la regeneración de tejidos. Hasta el momento no se han reportado efectos adversos de las infusiones o administración intra-lesional de las CTDTA. Se refiere un efecto benéfico inmunomodulador. [^27^](#qsh70q)  En 2011, Ra et al. [^29^](#1pxezwc) publicaron un estudio de 8 pacientes los cuales padecían lesión de la médula espinal que fueron tratados con infusiones intravenosas de CTDTA. El autor no observó efectos adversos serios. A las 12 semanas la función motora mejoró en 4 pacientes, el autor concluyó que no podían determinar la eficacia de la terapia con CTDTA por el pequeño grupo de pacientes y el corto periodo de seguimiento. [^27^](#qsh70q)  **Usos de CTDTA en cirugía plástica**  Las CTDTA han sido usadas en tres campos principalmente: el aumento de tejidos blandos, la cicatrización de heridas e ingeniería de tejidos. [^27^](#qsh70q) ([Tabla 3](#2nusc19)).  **Aumento de tejidos blandos**  Se han publicado varios estudios donde se utiliza la técnica de trasplante autólogo de tejido adiposo (TA) enriquecido con FEVTA , para tratamiento de atrofia facial,[^30^](#49x2ik5)^,^ [^31^](#2p2csry)^,^ [^32^](#147n2zr), aumento de mama con fines cosméticos y en el aumento de mama inmediato después de la extracción de un implante [^31^](#2p2csry)^,^ [^33^](#3o7alnk)^,^ [^34^](#23ckvvd), *pectus excavatum* [^31^](#2p2csry), pérdida de tejidos blandos por trauma o iatrogenia o enfermedades degenerativas [^31^](#2p2csry), tratamiento para cicatrices deprimidas [^27^](#qsh70q)^,^ [^35^](#ihv636) y para el tratamiento de escleroderma localizada (*en coup de sabre*) [^9^](#4d34og8). Estos autores reportan en todos sus casos mejoría en el aumento de volumen en tejidos blandos, mayor supervivencia del injerto, mejora en las características de la piel y concluyeron que la técnica para aumento de tejidos blandos es segura y efectiva. Sin embargo los estudios no fueron comparados con grupos control.  **Cicatrización de heridas**  Las CTDTA y su potencial para la cicatrización de heridas se ha mostrado en varios estudios *in vitro* o *in vivo***.** CTDTA y sus factores secretorios han mostrado mejoría en la curación de heridas crónicas en modelos animales y en ensayos clínicos. [^36^](#32hioqz) La primera aplicación clínica de CTDTA fue un reporte de caso donde se trató un defecto de “calvarium” después de una lesión. Después de tres meses del tratamiento, se reportó la formación de nuevo hueso y hubo una cicatrización de la herida casi completa.  Posteriormente Rigoti y Akita las han utilizado para el tratamiento en úlceras post-radioterapia con resultados favorables. [^27^](#qsh70q)  Hasta el momento el mecanismo de acción no se ha descrito adecuadamente. Se propone que el uso de CTDTA tenga efectos inmunomoduladores que provea condiciones favorables para el crecimiento tisular. Esto se especula ya que las CTDTA tienen la capacidad de secretar factores de crecimiento, citocinas y sustancias quimioatrayentes que mejoran la angiogénesis e incrementan el suministro de sangre a los tejidos así puede crear o suministrar soporte a las células. A la fecha, no se han reportado casos de neoplasias en pacientes tratados con lipoinyección o inyección de CTDTA directamente. Estudios clínicos recientes que usaron CTDTA en otras patologías no indicaron riesgo para la formación de tumores con el uso de CTDTA. Sin embargo, estudios de seguimiento a largo plazo son necesarios para establecer los efectos adversos del uso de estas células. [^36^](#32hioqz)  **Ingeniería de tejidos**  Los principios generales de las estrategias de la ingeniería de tejidos incorporan una combinación de tres factores: 1.- Las células vivas que están incrustadas en el sitio del defecto, 2.- Una estructura de protección tridimensional (3D) (andamio) de células en condiciones estructurales, funcionales y mecánicas características, 3.- Creación de un microambiente para proporcionar factores adicionales que finalmente promuevan el crecimiento y formación de nuevo tejido. Cuando el tejido nuevo está formado, la estructura del andamio biodegradable es reemplazada. [^37^](#1hmsyys)  Muchos biomateriales han sido investigados con el fin de ser utilizados como andamios en la construcción en ingeniería de tejido adiposo, tanto naturales como polímeros sintéticos. [^37^](#1hmsyys)  El avance de la ingeniería de tejidos como terapia regenerativa se basa en una rápida vascularización de las construcciones de los tejidos formados ex vivo por la vasculatura del huésped. Las células troncales mesenquimatosas pueden resultar beneficiosas para promover el ensamblaje rápido y el crecimiento de vasculatura para apoyar los resultados de ingeniería de tejidos in vivo. [^38^](#41mghml)  Bajo estas condiciones se han utilizado para la regeneración de tejido óseo [^39^](#2grqrue)^,^ cartílago [^40^](#vx1227), músculo [^37^](#1hmsyys) y nervio. [^41^](#3fwokq0)  **Estudios experimentales realizados con CTDTA**  Los estudios experimentales con CTDTA se han realizado principalmente en roedores. [^42^](#1v1yuxt)^,^ [^43^](#4f1mdlm)^,^ [^44^](#2u6wntf)^,^ [^45^](#19c6y18)^,^ [^46^](#3tbugp1)^,^ [^47^](#28h4qwu)^,^ [^48^](#nmf14n)^,^ [^49^](#37m2jsg)^,^ [^50^](#1mrcu09) También se han reportado algunos estudios en conejo [^51^](#46r0co2) y cerdo. [^52^](#2lwamvv) Han sido enfocados básicamente en la cicatrización de heridas en roedores diabéticos [^42^](#1v1yuxt), [^43^](#4f1mdlm)^,^ [^44^](#2u6wntf)^,^ [^45^](#19c6y18)^,^ [^46^](#3tbugp1)^,^ [^47^](#28h4qwu) y en roedores radiados. [^49^](#37m2jsg), [^50^](#1mrcu09) Se han aplicado en conjunto con andamios de submucosa de intestino delgado, matriz de dermis acelular y en andamios compuestos por colágeno, condroitín sulfato y ácido hialurónico. No se encontraron diferencias significativas entre estos. La cicatrización fue mejor en el grupo de CTDTA y andamios que en los grupos controles. No se comparó el uso de CTDTA con andamios contra CTDTA sin andamios [^16^](#1ksv4uv). Los resultados con la aplicación de CTDTA en forma aislada en heridas excisionales en ratones diabéticos han reportado aceleración de la cicatrización la cual ocurrió entre el día 9-18 comparada con los 28 días de los roedores diabéticos sin aplicación de CTDTA. [^42^](#1v1yuxt)^,^ [^44^](#2u6wntf)^,^ [^45^](#19c6y18)^,^ [^46^](#3tbugp1)^,^ [^47^](#28h4qwu) Se observó que el uso de CTDTA en heridas en ratones diabéticos igualó el tiempo de cicatrización comparada en las mismas heridas en ratones sanos sin aplicación de CTDTA. [^48^](#nmf14n) La cicatrización acelerada fue debida a la capacidad de síntesis y secreción de factores de crecimiento en un ambiente hipóxico, liberadas por las CTDTA. El estudio de Zhu et al. [^53^](#111kx3o) sustenta estos resultados mostrando que las CTDTA promueven el aumento en la expresión genética y secreción de grandes cantidades de factores de crecimiento angiogénicos, incluyendo el factor de crecimiento vascular endotelial (VEGF por sus siglas en inglés). Adicionalmente se ha reportado la secreción de TGF-β, KGF, FGF2, PDGF, HGF, fibronectina 1 y colágena 1. [^36^](#32hioqz) Lin et al. [^54^](#3l18frh) compararon láminas de CTDTA en multicapas contra láminas de CTDTA en una sola capa. Reportaron mejoría en la cicatrización de la herida en el modelo de herida excisional murino en el que usaron láminas de CTDTA en multicapas. [^16^](#1ksv4uv)  El estudio de Cianfarani et al. [^55^](#206ipza) sugiere que las comorbilidades como la diabetes, podría afectar el potencial de las CTDTA en modelos murinos, aunque las implicaciones clínicas de esta aseveración no han sido establecidas. Nambu et al. [^42^](#1v1yuxt) han sido los únicos que han utilizado modelos murinos diabéticos y ellos aún encontraron mejoría en la cicatrización de heridas. Esto sugiere que las comorbilidades como la diabetes podrían no tener efecto en el potencial de las CTDTA. [^16^](#1ksv4uv)  El-Ftesi et al. [^57^](#4k668n3) mostraron que las CTDTA provenientes de ratones diabéticos añosos tenían una respuesta alterada a la hipoxia (un decremento en la expresión de FEVTA) cuando se comparó con CTDTA de ratones sanos y saludables.  **Complicaciones del uso de CTDTA**  En la mayor parte de los estudios publicados en los cuales se ha utilizado CTDTA no se han reportado complicaciones o efectos adversos, aunque el uso clínico de CTDTA es nuevo. [^26^](#2bn6wsx)  En algunos ensayos clínicos donde se utilizaron lipoinjertos enriquecidos con CTDTA se observó el desarrollo de sangrado subcutáneo así como inflamación de 4 semanas de duración en seis pacientes con lipoatrofia facial (un paciente con diagnóstico de síndrome de Parry-Roomberg y los cinco restantes diagnosticados con lupus eritematoso profundo). Se dividieron en dos grupos el control consistió en tres pacientes a los que se les inyectó tejido graso subcutáneo en el área con lipoatrofia, mientras que el grupo experimental conformado por tres pacientes se inyectó tejido graso enriquecido con CTDTA. No se realizaron maniobras terapéuticas para tratar el sangrado subcutáneo que presentaron algunos pacientes. Dicha complicación resolvió en todos los pacientes pasadas 1 a 2 semanas [^30^](#49x2ik5). En ensayos clínicos realizados se observó la presencia de fibrogénesis ectópica y linfadenopatía distal en dos pacientes. [^57^](#2zbgiuw) Se ha reportado también comportamiento desfavorable de células troncales mesenquimales, como la diferenciación a miofibroblastos. [^58^](#1egqt2p) Por este motivo se sugiere que las CTDTA deberían estar adheridas a células, tejidos, o andamios biológicos antes de ser administradas, para evitar la migración o diferenciación inesperada. [^59^](#3ygebqi)  En algunas publicaciones se concluye que las CTDTA pueden aumentar el crecimiento de células tumorales activas, pero no de las que están en descanso (G0). [^26^](#2bn6wsx)^,^ [^60^](#2dlolyb)  En un estudio en el cual se inyectaron CTDTA humanas a ratones inmunodeficientes en tejido subcutáneo no se encontró formación de teratomas. [^15^](#35nkun2)  **Estatus actual, incertidumbre de aplicación y regulación.**  La “Food and Drug Administration” (FDA) ha desarrollado un marco de regulación basado en tres áreas: la prevención del uso de tejidos y células contaminadas; la prevención del manejo y procesamiento inadecuado que pudiera dañar o contaminar a las células o tejidos; y la seguridad clínica de todos los tejidos y células que pudieran ser procesados, usados para otros fines diferentes a sus funciones normales, su combinación con otros componentes además de los tejidos o los usados con fines metabólicos. En los Estados Unidos de América, las CTDTA están consideradas en el contexto de células humanas, tejidos humanos por lo que sus productos y su producción deben cumplir con los requerimientos actuales de la buena práctica de tejidos, reglamentado bajo el Código de las Regulaciones Federales, Título 21, Parte 1271. [^27^](#qsh70q)  En Europa las CTDTA son consideradas productos medicinales de terapia avanzada, definido de esta manera por la Regulación Europea (Comisión Europea), la cual contiene reglas para la “autorización”, supervisión y requerimientos técnicos con respecto al resumen de las características del producto, el etiquetado, y el empacado de los productos medicinales de terapia avanzada que son preparados por la industria y por las instituciones académicas. [^27^](#qsh70q)  El proceso de conversión de protocolos basados en la investigación que utilizan CTDTA a un proceso de fabricación seguro que cuenta con un buen proceso de manufacturación requiere protocolos que hayan tenido cuidadosas consideraciones acerca de todos los riesgos y beneficios para el paciente. Sensebé et al. establecieron los siguientes parámetros que debieran ser considerados: fuentes y métodos de recolección, siembra de células, la velocidad de proliferación y el medio de cultivo. [^27^](#qsh70q)  En México la Ley General de Salud publicó su última reforma en el Diario Oficial de la Federación el 15/01/2014. Ésta estipula en su Título XIV (donación, trasplantes y pérdida de vida) en el artículo 330 lo siguiente:  Artículo 330: El Centro Nacional de Trasplantes tendrá a su cargo el Registro Nacional de Trasplantes, el cual integrará y mantendrá actualizada la siguiente información:   1. El registro de establecimientos autorizados conforme al artículo 315 de esta Ley; 2. Los cirujanos de trasplantes responsables de la extracción y trasplantes, y los coordinadores hospitalarios de la donación; 3. Los datos de los trasplantes con excepción de los autotrasplantes y los relativos a células progenitoras o troncales;   El registro de los trasplantes de células progenitoras o troncales estará a cargo del Centro Nacional de la Transfusión Sanguínea. [^61^](#sqyw64)  Respecto al uso de FEVTA en escleroderma localizada y sistémica hay pocos estudios, la mayoría son reportes de casos aislados, que han mostrado mejoría en las lesiones cutáneas. Por ejemplo, hay reportes de la aplicación de injertos de grasa para escleroderma localizada (*en golpe de sable*), Consorti et al. reportaron el caso de una mujer de 34 años de edad afectada con escleroderma localizada en la región fronto-orbitaria asimetría en el arco de la ceja ambas en el lado derecho de la cara. Se le realizaron injertos grasos. Después de dos años de seguimiento la condición del paciente fue satisfactoria, con mejoría en la simetría y morfología de la región fronto-orbitaria así como en la atrofia celular y la textura. [^6^](#tyjcwt) Oh et al. reportaron un reporte de un hombre de 21 años con una depresión atrófica trilinear en la frente y la porción frontal del cuero cabelludo, secundarias a escleroderma localizada. Se le inyectó injerto de grasa 50% e injerto de dermis 50% en las regiones con depresión. El paciente presentó elevación convexa de la forma en el postoperatorio hasta aproximadamente los dos meses cuando las áreas inyectadas ya presentaban características similares a las áreas circundantes y la apariencia café obscuro de las líneas atróficas habían desaparecido. [^7^](#3dy6vkm) Se concluye que el uso de injertos grasos con FEVTA tiene buenos resultados y no se reportan complicaciones [^5^](#2et92p0). Bank et al. Realizaron una serie de 13 casos (12 mujeres y un hombre) de pacientes. Nueve tenían escleroderma, dos poseían enfermedad mixta del tejido conectivo y dos padecían fenómeno de Raynaud primario. Cada mano con manifestaciones fue tomada por separado para la aplicación de grasa en manos. Un total de 21 manos fueron tratadas. En el pretratamiento se evaluó dolor y nivel funcional. Al concluir el estudio se encontró una mejoría en el dolor después de la aplicación de tejido graso (p<0.001) 18 de las 21 manos tuvieron disminución en el dolor, las 3 sobrantes no presentaron cambios en el dolor. 20 de las 21 manos presentaron mejoría en los ataques de frío (p<0.001) así como mejoría en la severidad de los ataques de frío, ya que las 21 manos tuvieron disminución de la misma (p<0.001). 12 de las 21 manos tenían ulceras antes del tratamiento; 5 de las 21 manos continuaron con úlceras después del tratamiento. Como conclusión Bank et al. describen que este procedimiento parece ser seguro y sencillo para el tratamiento de este padecimiento. La mejoría de los pacientes se atribuye a la angiogénesis y una disminución en las vías que fomentan la cicatrización y fibrosis, esto atribuido a la FEVTA. [^8^](#1t3h5sf) Experimentalmente también se ha utilizado para el tratamiento de lesiones de piel ocasionadas por esclerosis sistémica, a los ratones se les inyectó bleomicina subcutánea para provocar esclerosis de la piel. Una vez provocada la esclerosis se inyectó a las ratas FEVTA en el sitio de la esclerosis mostrando una disminución en la fibrosis de la dermis y un efecto proangiogénico [^5^](#2et92p0).  Lo anterior ha atraído la atención para la aplicación de FEVTA en el tratamiento de úlceras digitales producidas por esclerosis sistémica. Sólo este autor ha utilizado FEVTA para el tratamiento de esclerosis sistémica localizada (*en coup de sabre*) con buenos resultados [^9^](#4d34og8)  Actualmente existe un protocolo (SCLERADEC [NCT01813279]. www.clinicaltrials.gov) llevado a cabo en Marsella, el cual tiene como objetivo evaluar los efectos de la inyección de la fracción estromal vascular de tejido adiposo (FEVTA) autólogo en pacientes con esclerosis sistémica que presentan alteraciones funcionales en manos, realizando una inyección subcutánea de la FEVTA en los dedos en contacto con pedículos neurovasculares en 11 pacientes con escleroderma con seguimiento a un año. Resultados preliminares del grupo de la Dra. Del Papa mostraron disminución en el tiempo de curación de las úlceras y mejoría en la cantidad de capilares en 15 pacientes con ES a quienes se les administraron células troncales derivadas de tejido adiposo autólogo (Del Papa N, Di Luca G, Sambataro D, et al. Regional implantation of adipose tissue-derived cells induces a prompt healing of long-lasting indolent digital ulcers in patients with systemic sclerosis. Arthritis & Rheumatology 2014; supplement 1, S737).  Asimismo, hay datos que sugieren que la administración de células troncales hematopoyéticas autólogas (que se encuentran en gran cantidad en el FEVTA) puede producir mejoría clínica en diversos parámetros clínicos, vasculares e inmunológicos en pacientes con esclerosis sistémica. Ejemplo de ello son los resultados del estudio ASTIS que reclutó a pacientes con ES grave, temprana y mostró que, si bien hubo mayor mortalidad temprana asociada al trasplante (por infecciones graves), dicho tratamiento se asoció a mayor sobrevida libre de enfermedad a largo plazo, disminución en la fibrosis cutánea y estabilización del daño a órganos internos.[^83^](#39kk8xu)  Existe también evidencia indirecta proveniente de reportes de casos. Por ejemplo: Guiducci et al, reportan el caso de una paciente con diagnóstico de esclerosis sistémica, la cual desarrolló gangrena aguda en extremidades. El tratamiento consistió en 3 infusiones intravenosas de células troncales mesenquimales autólogas obtenidas de médula ósea. Los resultados de la angiografía y la alta expresión de factores angiogénicos en la regeneración de tejidos obtenidos una semana después de la administración del tercer pulso, sugieren que las células troncales mesenquimales promovieron la formación de nuevos vasos y la remodelación vascular en las extremidades. [^10^](#2s8eyo1) |
| **9. Definición del problema** |
| El tratamiento convencional actual de las UDES consiste en vasodilatadores, antiagregantes plaquetarios, analgésicos, el control de la esclerosis sistémica, de enfermedades concomitantes y curaciones continuas para evitar infecciones locales hasta obtener la cicatrización secundaria. Durante este período los pacientes necesitan de múltiples consultas y curaciones, tienen dolor y discapacidad importante la cual puede ser irreversible y riesgo de infecciones y pérdida de tejido. |
| **10.Justificación** |
| Debido a dolor, al tiempo prolongado necesario para obtener la cicatrización secundaria, a la discapacidad que producen y al alto costo que genera para el paciente el tratamiento de las UDES, se propone la aplicación local de fracción estromal vascular derivada del tejido adiposo, ya que hay evidencia experimental y clínica que sugiere que acelera el proceso de cicatrización de heridas crónicas asociadas a vasculopatía. Al no haber ensayos clínicos controlados previos que evalúen el uso de FEVTA en esta patología, proponemos un estudio piloto para evaluar la seguridad y obtener datos que permitan calcular un tamaño de muestra para posteriormente diseñar un estudio donde se evalúe eficacia del uso de FEVTA. |
| **11. Hipótesis** |
| El uso de lipoinjerto enriquecido con la fracción vascular del tejido adiposo inyectado subcutáneamente en pacientes con UDES, será seguro en pacientes con UDES secundarias a ES.  Como hipótesis secundaria: El uso de lipoinjerto enriquecido con la fracción vascular del tejido adiposo inyectado subcutáneamente en pacientes con UDES permitirá acelerar el proceso de cicatrización secundaria comparado con el tratamiento médico y quirúrgico convencionales |
| **12. Objetivos**. |
| **Objetivo principal**  Evaluar la seguridad de la administración de fracción estromal vascular del tejido adiposo (autólogo) en pacientes con úlceras digitales isquémicas asociadas a esclerosis sistémica.  **Objetivos Secundarios.**   - 1. Valorar el efecto del tratamiento propuesto sobre el tiempo de curación de la úlcera índice.   2. Evaluar la mejoría en el dolor en mano.   3. Evaluar la mejoría en la función de la mano.   4. Evaluar la mejoría en la calidad de vida.   5. Evaluar el efecto del tratamiento propuesto en el número de capilares ungueales por videocapilaroscopía.   6. Evaluar el efecto inmunomodulador de la administración de FEVTA en pacientes con UDES.   7. Evaluar la reproducibilidad del procedimiento de extracción de la fracción vascular del tejido adiposo (FEVTA).   8. Como objetivo exploratorio deseamos determinar si la administración de FEVTA en pacientes con UDES disminuye la concentración de los siguientes marcadores inflamatorios: velocidad de sedimentación globular (VSG), proteína C reactiva (PCR), endotelina-1, (ET-1), molécula de adhesión intercelular (ICAM-1), interleucina 1 (IL-1), interleucina 6 (IL-6), interleucina 17 (IL-17), interleucina 22 (IL-22), factor de crecimiento vascular endotelial (VEGF). |
| **13. Metodología: Diseño general.** |
| Prospectivo, longitudinal, aleatorizado, controlado, no cegado.  **OBTENCION Y AISLAMIENTO DE FEVTA:**  En la valoración inicial se solicitarán radiografías de manos en proyección AP y oblicua, así como a las pacientes en edad fértiles se realizará prueba de embarazo para corroborar que no se encuentre embarazada, ya que el embarazo de motivo de exclusión.  El procedimiento se realizará en los quirófanos de la unidad del paciente ambulatorio (UPA) del INCMNSZ. Preoperatoriamente se efectuará oximetría percutánea de todos los dedos de la mano, con un oxímetro portátil, para determinar grado de perfusión como control.  Con el paciente de pie se marcarán los depósitos de grasa susceptibles a realizar liposucción para extracción de grasa.  Como profilaxis se aplicará una dosis IV de Amoxicilina y Sulbactam 1g/500 mgr IV o en caso de ser alérgico Ciprofloxacino a dosis de 400 mg IV DU. Adicionalmente se aplicará Ketorolaco 30 mg IV DU. Se administrará anestesia local con solución de Klein (Solución Hartmann 1000 ml, 25 cc de Xilocaina al 2% simple, l ml de adrenalina y 10 mEq de bicarbonato) y sedación intravenosa.  Se infiltrarán de 100 a 200 ml solución de Klein, (técnica tumescente) en el sitio de mayor concentración de tejido adiposo subcutáneo susceptible a liposucción. Quince minutos después se hará liposucción del área, con una cánula roma con orificios de 2 mm de diámetro y una jeringa de 20 ml. Se extraerá un mínimo de 100 ml de grasa. De esta cantidad 60 ml serán colocados en frascos estériles con de Solución Salina Balanceada de Hank (HBSS) con albúmina al 5% y se transportará inmediatamente al Departamento de Fisiología de la Nutrición del INCMNSZ para su procesamiento.  La fracción vascular se aislará del tejido adiposo por el método de separación con colagenasa descrito por Rodbell de la siguiente manera:  El tejido se lavará con solución salina amortiguada por fosfatos (PBSIx) estéril con antibiótico-antimicótico (1%) a 37°C dos veces para eliminar la sangre residual, posteriormente se incubará por 40 min en una solución de colagenasa al 5% en HBSS. Una vez disgregadas, las células se filtrarán en una malla de 100 micras y se centrifugan a 1000 rpm por 5 min para separar las células del estroma vascular de los adipocitos. Se elimina el sobrenadante y las células compactadas se resuspenden en 30 ml de HBSS estéril a 37ºC, se cuantifica la densidad y viabilidad.  Cuarenta ml de grasa aspirada serán colocados en una jeringa de 50 ml y será mantenido en reposo, para decantarla. La sangre y suero que se separen de la grasa, serán eliminados.  El procedimiento será realizado bajo lo estipulado en la Ley General Título XIV (donación, trasplantes y pérdida de vida) artículo 330.  **APLICACIÓN DE FEVTA:**  El estroma vascular extraído y caracterizado previamente será mezclado con los 40 ml. de grasa que fueron mantenidos en la jeringa de 50 ml. Se aplicará anestesia local con Xilocaina al 2% sin epinefrina en el nervio mediano, ulnar y radial a nivel de la muñeca, con el objeto de obtener anestesia de toda la mano. Después de mezclarlos, esta solución será colocada en jeringas de 1 ml y 3 ml. Con una aguja de 19 gauge (0.8 mm) se aplicará 0.5 ml en el borde radial y ulnar de cada falange de cada dedo (3 ml por dedo) y 10 ml subcutáneamente distribuidos por todo el dorso de la mano, y 5 ml subcutáneos distribuidos en la región palmar.  Se realizará oximetría digital por dedo un ahora después de haber terminado la infiltración de grasa. Así mismo se evaluará el dolor y se aplicará analgésicos IV en caso necesario.  Un apósito oclusivo será colocado en las úlceras digitales.   1. Tamaño de la muestra. Se trata de un estudio exploratorio para evaluar la reproducibilidad del procedimiento de extracción de la FEVTA y la seguridad del lipoinjerto en pacientes con úlceras digitales isquémicas asociadas a esclerosis sistémica. Los resultados obtenidos en este estudio nos servirán como base para calcular el tamaño de muestra para un estudio posterior donde se evaluará la eficacia del lipoinjerto en estos pacientes. 2. Mecanismo de asignación del tratamiento: aleatorio. 3. Grupos de tratamiento: a) Grupo control con tratamiento convencional. b) Grupo de estudio con tratamiento convencional más aplicación de la fracción vascular estromal del tejido adiposo. 4. Duración del seguimiento individual: 6 meses a partir de la visita cero. |
| **14. Temporalidad del estudio.** |
| \| **Tipo de estudio** \| **Seleccione una opción** \| \| --- \| --- \| \| Retrospectivo \|  \| \| Prospectivo \| X \| |
| **15.Proceso de asignación de los grupos en estudio** |
| \| Maniobra \| Si (Incluya la información correspondiente) \| No \| No aplica \| \| --- \| --- \| --- \| --- \| \| Aleatorización \| X \|  \|  \| \| Estudio abierto \|  \| x \|  \| \| Estudio ciego simple \|  \| X \|  \| \| Estudio doble ciego \|  \| X \|  \| \| Estudio triple ciego \|  \| X \|  \| |
| **16.Descripción de las maniobras o las intervenciones** |
| Una vez que el paciente haya completado el procedimiento de consentimiento informado se realizará la aleatorización para determinar el grupo en el que participará el paciente y se agendará la visita al día inicial que se llamará día cero.  **Visita día cero.**  **Fase Pre-tratamiento:**  A todos los participantes en el ensayo clínico se les realizara una evaluación inicial que constara de:   1. Evaluación de las ulceras secundarias a esclerosis sistémica (se evaluará la cantidad, la localización, tamaño de las mismas). 2. Se realizarán fotografías de las manos con úlceras (de la región palmar y dorsal). 3. Se realizará una videocapilaroscopía, estudio que nos ayudará a conocer cambios en la microcirculación digital. 4. Se realizará una oximetría transcutánea con oxímetro portátil en cada dedo, esto con el fin de conocer el grado de perfusión. 5. Se realizará evaluación del dolor (Escala visual análoga). 6. Se realizarán evaluaciones de funcionalidad con los cuestionarios SHAQ y COCHIN. 7. Se realizará cuestionario para conocer su calidad de vida (SF 36). 8. Se realizarán radiografías AP y oblicuas de manos. 9. Se realizará evaluación estándar de la esclerosis sistémica que incluye determinación de puntaje de Rodnan modificado y los parámetros de la escala de severidad de Medsger. Este seguimiento es el que se realiza a todos los participantes de la cohorte de esclerosis sistémica del Instituto periódicamente. 10. Se realizará prueba inmunológica de embarazo a todas las participantes mujeres en edad fértil. 11. Se prescribirá tratamiento estándar para las úlceras digitales isquémicas que incluye los medicamentos permitidos según lo descrito en criterios de inclusión y exclusión del protocolo. 12. En la visita del día 0 y en la del día 84 se tomará una muestra de sangre venosa periférica. Se aislarán células mononucleares de sangre periférica (PBMC) por gradiente de centrifugación con Ficoll-Paque (Amersham Bioscience, Diegem, Bélgica) y se obtendrá suero a partir de una muestra de 20 ml de sangre periférica.   Se detectarán subpoblaciones de células T (Th1, Th2, Th17, Treg) s con la metodología previamente publicada, la cual se resume a continuación:[^62^](#3cqmetx)  Se teñirán 1 x 10^6^ células mononucleares de sangre periférica (PBMCs) con 5 μL de anticuerpos monoclonales anti-CD4 teñido con PECy5 y anti-CD14 teñido con FITC (BD Biosciences, San José CA), a temperatura ambiente, en la oscuridad, por 20 min. Después de 2 lavados, las PBMCs se permeabilizarán con 200 μL de solución de citofix/citoperm (BD Biosciences) a 4°C por 20 min; después de 2 lavados con solución permwash (BD, Biosciences) las PBMCs se teñirán para citocinas intracelulares y factores de transcripción con anti-IFNγ teñido con PE para células Th1 (BD, Biosciences), anti-IL-4 teñido con PE para células Th2 (BD, Biosciences), anti-IL-17 teñido con PE para células Th17 (clona eBio64CAP17, IgG1_1_K de ratón, de eBioscience, San Diego, CA) y anti-Foxp3 teñido con PE para células T reguladoras (eBioscience; clona 259D/C7, IgG1K de ratón) por 30 min a 4°C en la oscuridad. Finalmente, después de lavar con solución permwash, se analizarán los subtipos de PBMCs con citometría de flujo con un FACScan (BD Biosciences). Se utilizará una ventana para células CD4+/CD14-, se registrarán 50,000 eventos para cada muestra y se analizarán con el programa CellQuest (BD, Biosciences). Los resultados se expresarán como el porcentaje de células que expresen IFNγ, IL-4, IL-17 o Foxp3 en cada ventana. Se utilizarán controles de isotipos (IgG_1_-FITC/IgG_1_-PE/CD45-PeCy5/IgG_1_k murinos (BD Tritest, BD Biosciences) para establecer los umbrales y ventanas en el citómetro. Para evitar resultados falsos positivos de PE y para establecer la compensación para el análisis por citometría multicolor se realizarán procedimientos de calibración del instrumental cada día de acuerdo con los protocolos establecidos por nuestro laboratorio. Brevemente, se realizará un corrimiento sin colorante (control de autofluorescencia) y un corrimiento con una muestra de PBMCs permeabilizadas. El control de autofluorescencia (células no teñidas) se comparará con controles positivos con tinción única para confirmar que las células teñidas estuvieran en escala para cada parámetro. Además, se utilizarán 3 perlas de calibración de BD para ajustar los parámetros del instrumento, establecer compensaciones de fluorescencia y revisar la sensibilidad del instrumento (BD CaliBRITE^TM^, BD, Biosciences).  Se medirán velocidad de sedimentación globular (VSG), proteína C reactiva (PCR), endotelina-1, (ET-1), molécula de adhesión intercelular (ICAM-1), interleucina 1 (IL-1), interleucina 6 (IL-6), interleucina 17 (IL-17), interleucina 22 (IL-22), factor de crecimiento vascular endotelial (VEGF). La VSG se determinará el día de la toma de la muestra por Westergern, la PCR ultrasensible se determinará el día de la toma de la muestra por nefelometría. El resto de la muestra se congelará para su posterior procesamiento por luminometría siguiendo las instrucciones del fabricante (Bioplex, Biorad, EUA).  A los pacientes que se asigne al tratamiento con FEVTA, el día cero, además de las evaluaciones antes descritas, se realizarán los siguientes procedimientos:  **OBTENCION Y AISLAMIENTO DE FEVTA:**  El procedimiento se realizará en los quirófanos de la unidad del paciente ambulatorio (UPA) del INCMNSZ. Preoperatoriamente se efectuará oximetría percutánea de todos los dedos de la mano, con un oxímetro portátil, para determinar grado de perfusión como control.  Con el paciente de pie se marcarán los depósitos de grasa susceptibles a realizar liposucción para extracción de grasa.  Como profilaxis se aplicará una dosis IV de Amoxicilina y Sulbactam 1000/500 mg IV o en caso de ser alérgico Ciprofloxacino a dosis de 400 mg IV DU. Adicionalmente se aplicará Ketorolaco 30 mg IV DU. Se administrará anestesia local con Solución de Klein (Solución Hartmann 1000 ml, 25 cc de Xilocaína al 2% simple, l ml de adrenalina y 10 MEq de bicarbonato) y sedación intravenosa.  Se infiltrarán de 100 a 200 ml solución de Klein, (técnica tumescente) en el sitio de mayor concentración de tejido adiposo subcutáneo susceptible a liposucción. Quince minutos después se hará liposucción del área, con una cánula roma con orificios de 2 mm de diámetro y una jeringa de 20 ml. Se extraerá un mínimo de 100 ml de grasa. De esta cantidad 60 ml serán colocados en frascos estériles con de Solución Salina Balanceada de Hank (HBSS) con albúmina al 5% y se transportará inmediatamente al Departamento de Fisiología de la Nutrición del INCMNSZ para su procesamiento.  La FEVTA se aislarán del tejido adiposo por el método de separación con colagenasa descrito por Rodbell de la siguiente manera:  El tejido se lavará con solución salina amortiguada por fosfatos (PBSIx) estéril con antibiótico-antimicótico (1%) a 37°C dos veces para eliminar la sangre residual, posteriormente se incubará por 40 min en una solución de colagenasa al 5% en HBSS. Una vez disgregadas, las células se filtrarán en una malla de 100 micras y se centrifugan a 1000 rpm por 5 min para separar las células del estroma vascular de los adipocitos. Se elimina el sobrenadante y las células compactadas se resuspenden en 30 ml de HBSS estéril a 37ºC, se cuantifica la densidad y viabilidad.  Cuarenta ml de grasa aspirada serán colocados en una jeringa de 50 ml y será mantenido en reposo, para decantarla. La sangre y suero que se separen de la grasa, serán eliminados.  **APLICACIÓN DE LIPOINJERTO ENRIQUECIDO CON FEVTA:**  El estroma vascular extraído y caracterizado previamente será mezclado con los 40 ml. de grasa que fueron mantenidos en la jeringa de 50 ml. Se aplicará anestesia local con Xilocaina al 2% sin epinefrina en el nervio mediano, ulnar y radial a nivel de la muñeca, con el objeto de obtener anestesia de toda la mano. Después de mezclarlos, esta solución será colocada en jeringas de 1 ml y 3 ml. Con una aguja de 19 gauge (0.8 mm) se aplicará 0.5 ml en el borde radial y ulnar de cada falange de cada dedo (3 ml por dedo) y 10 ml subcutáneamente distribuidos por todo el dorso de la mano, y 5 ml subcutáneos distribuidos en la región palmar.  Se realizará oximetría digital por dedo un ahora después de haber terminado la infiltración de grasa. Así mismo se evaluará el dolor y se aplicará analgésicos IV en caso necesario.  Un apósito oclusivo será colocado en las úlceras digitales.  **Fase de seguimiento y evaluación de resultados**  Independientemente del grupo al que sea asignado se llevará a cabo seguimiento primero semanal y después cada 28 días (cada mes) hasta el día 168 (hasta los 6 meses).  En las visitas de los días 7, 14, 21, 28, 56, 84, 112, 140 y 168 Se realizará a todos los pacientes:  1) Evaluación de las úlceras secundarias a esclerosis sistémica (se evaluará la cantidad, la localización, tamaño de las misma) la cual se llevará a cabo semanalmente durante el primer mes y posteriormente mensual (días 28, 56, 84, 112, 140, 168) hasta los 6 meses (día 168).  2) Se le realizarán radiografías de mano en 2 posiciones (en la visita cero y a los 168 días, 6 meses).  3) Se realizarán fotografías de las manos con úlceras (de la región palmar y dorsal) las cuales se llevarán a cabo semanalmente durante el primer mes y posteriormente mensualmente (días 28, 56, 84, 112, 140, 168) hasta los 6 meses (día 168).  4) Se realizará videocapilaroscopía en la visita basal, la del día 28 y del día 168 (6 meses).  5) Se realizará oximetría transcutánea con oxímetro portátil en cada dedo, esto con el fin de evaluar la perfusión inmediatamente después de la cirugía, semanal mente durante el primer mes y posteriormente mensual hasta los 6 meses.  6) Se realizará evaluación del dolor semanalmente durante el primer mes y después, mensualmente hasta los 6 meses (días 28, 56, 84, 112, 140, 168).  7) Se administrarán los cuestionarios SHAQ y COCHIN a la semana de postquirúrgico, al mes (día 28) y a los 6 meses (día 168).  8) Se realizará cuestionario para conocer calidad de vida (SF 36) a la semana de postquirúrgico, al mes (día 28) y a los 6 meses (día 168). |
| **17.Tratamientos (si aplica) (incluya una tabla para cada medicamento en estudio)** |
| \| Medicamento 1 \| Incluya la información correspondiente \| No \| No aplica \| \| --- \| --- \| --- \| --- \| \| Nombre \|  \|  \| X \| \| ¿Cumple con “Buenas prácticas de fabricación”? \|  \|  \| X \| \| Códigos, etiquetado, almacenamiento, retención y resguardo de las muestras de medicamento \|  \|  \| X \| \| Forma farmacéutica \|  \|  \| X \| \| Dosis \|  \|  \| X \| \| Intervalo de administración \|  \|  \| X \| \| Vía de administración \|  \|  \| X \| \| Velocidad de administración \|  \|  \| X \| \| Duración del tratamiento \|  \|  \| X \| |
|  |
| **18. Seguimiento** |
| \|  \| Incluya la información correspondiente \| No \| No aplica \| \| --- \| --- \| --- \| --- \| \| Número de fases del estudio \|  \|  \| X \| \| Número de visitas y su programación (incluya los horarios) \| 10 visitas en horario matutino \|  \|  \| \| Duración de cada fase del estudio \|  \|  \| X \| \| Estudios de laboratorio y gabinete que serán usados \| Radiografías AP y oblicuas de ambas manos  Videocapilaroscopía \|  \|  \| \| Duración total del seguimiento \| 168 días \|  \|  \| \| Métodos de muestreo \|  \|  \| X \| \| Opciones de tratamiento que se ofrecerán al término del estudio \|  \|  \| X \| |
|  |
| **19. Manejo de sobredosis** |
| No aplica |
| **20. Terapia de rescate** |
| Curación de heridas, analgésicos, antibioticoterapia, antiagregantes plaquetarios y terapia con cámara hiperbárica. |
| **21. Terapias concomitantes permitidas** |
| Tratamiento habitual según indicaciones de su médico tratante, excepto los descritos en la sección 22. |
| **22. Terapias concomitantes prohibidas** |
| 1. Pacientes con tratamiento vasodilatador (como antagonistas de canales de calcio, IECA, nitroglicerina, bloqueadores alfa adrenérgicos, antagonistas de receptores II de angiotensina, inhibidores de 5 fosfodiesterasa, inhibidores de receptores de endotelina, prostanoides), N-acetilcisteína, tratamiento antiagregante, heparina convencional o de bajo peso molecular, que han recibido este tratamiento por menos de 2 semanas previas a la visita de selección o cuyo tratamiento no ha estado estable por este periodo. 2. Tratamiento inmunomodulador (como prednisona o su equivalente, ciclofosfamida, azatioprina, ácido micofenólico, D-penicilamina, metotrexato, cloroquina, hidroxicloroquina, leflunomida o cualquier otro inmunosupresor) que se haya iniciado en los 3 meses previos a la visita de selección o cuyo tratamiento no haya estado estable por al menos 1 mes previo a la visita de selección. 3. Uso de factores de crecimiento tópicos u oxígeno hiperbárico concomitante al estudio. 4. Inyección local de toxina botulínica durante el estudio o hasta 4 semanas previas a la visita de inicio. 5. Simpatectomía quirúrgica de extremidades superiores o debridación de la herida quirúrgica dentro del mes previo a la visita de inicio. 6. Tratamiento concomitante con otro medicamento experimental dentro de las 4 semanas previas al estudio o durante el estudio. |
| **23. Definición de las variables de seguimiento** |
| Presencia de eventos adversos  Tiempo de cicatrización de las UDES  Mejoría en el dolor de la mano  Mejoría en la función de la mano  Mejoría en la calidad de vida  Número de capilares por campo en la videocapilaroscopía  Efecto inmunomodulador del tratamiento (proporciones de subpoblaciones de células TCD4+)  Niveles de marcadores de inflamación y citocinas |
| **24. Métodos que se usarán para la recolección de la información** |
| Se utilizará una tabla en Excel en la cual se recolectarán los datos de las variables en estudio (se anexa como apéndice). |
| **25. Procedimiento de monitoreo y auditorias durante el desarrollo del estudio** |
| No aplica. |
| **26. Criterios de falla y de éxito** |
| Éxito (desenlace primario).  Cicatrización total de la úlcera digital índice dentro del período de observación, con mejoría o sin cambios en: el dolor, en la función de la mano y en la calidad de vida.  Mejoría (desenlaces secundarios).   1. Mejoría en el dolor en mano. 2. Mejoría en la función de la mano. 3. Mejoría en la calidad de vida. 4. Aumento en el número de capilares por campo en la videocapilaroscopía.   Falla.   1. Falta de cicatrización dentro del período de estudio. 2. Aumento del dolor de acuerdo a la escala realizada previo tratamiento. 3. Deterioro en la función de la mano. 4. Deterioro en la calidad de vida.   Eventos adversos:   1. Infección de la úlcera o del sitio de inyección de FEVTA. 2. Infección del sitio de obtención del tejido adiposo. 3. Necrosis tisular en el sitio de aplicación de FEVTA. 4. Deterioro en la función de la mano. 5. Deterioro en la calidad de vida. 6. Aumento del dolor. 7. Aparición de nuevas úlceras. 8. Cualquier evento que requiera hospitalización no programada del paciente (relacionado o no con el tratamiento), prolongación de una hospitalización o muerte se considerará evento adverso grave. |
| **27. Tamaño de muestra (por favor incluya la fórmula empleada para el cálculo y la fuente de información en que se fundamentaron los supuestos)** |
| Se trata de un estudio piloto para evaluar la seguridad de la administración de FEVTA en úlceras digitales de pacientes con esclerosis sistémica. No hay estudios previos que permitan calcular adecuadamente el tamaño de muestra por lo que los resultados de este estudio darán información para poder calcular el tamaño de muestra para un estudio en el que se evaluará la eficacia de FEVTA en el tiempo de curación de úlceras digitales en pacientes con ES. |
| **28. Descripción de las técnicas, aparatos y/o instrumentos que se utilizarán en la medición (Incluidos: equipos mecánicos, electrónicos, cibernéticos especiales)** |
| Videocapilaroscopía. Videocapilaroscopio Optilia 200x.  Oxímetro de pulso omron o equivalente. |
| **29. Descripción de los formatos de evaluación, cuestionarios, tablas de cotejo, etc., señalando los criterios de validez, reproducibilidad y controles de calidad que se tengan de los mismos** |
| COCHIN  SHAQ  SF-36  (se anexan como apéndices) |
| **30. ¿El protocolo implica el manejo y etiquetado de muestras biológicas? En caso de ser aplicable, mencione los procedimientos que se usarán** |
| El tejido adiposo obtenido de cada paciente se rotulará con el nombre y número de registro de cada paciente, así como la fecha de la obtención. También se le asignará a cada paciente un número consecutivo que se anexará a las etiquetas del tejido adiposo y de todos los tubos. Durante todo el procesamiento del tejido y la obtención de la FEVTA, todos los tubos se marcarán con las iniciales, el número de registro de cada paciente y el número de protocolo asignado, ya que se trata de un estudio abierto (no cegado) no es necesario codificar las muestras para cegarlas.  Si, Con el paciente de pie se marcaran los depósitos de grasa susceptibles a realizar liposucción para extracción de grasa.  Como profilaxis se aplicará una dosis IV de Amoxicilina y Sulbactam 1g/500 mg IV o en caso de ser alérgico Ciprofloxacino a dosis de 400 mg IV DU. Adicionalmente se aplicará Ketorolaco 30 mg IV DU. Se administrará anestesia local con solución de Klein (solución Hartmann 1000 ml, 25 cc de Xilocaina al 2% simple, l ml de adrenalina y 10 mEq de bicarbonato) y sedación intravenosa.  Se infiltrarán de 100 a 200 ml solución de Klein, (técnica tumescente) en el sitio de mayor concentración de tejido adiposo subcutáneo susceptible a liposucción. Quince minutos después se hará liposucción del área, con una cánula roma con orificios de 2 mm de diámetro y una jeringa de 20 ml. Se extraerá un mínimo de 100 ml de grasa. De esta cantidad 60 ml serán colocados en frascos estériles con de Solución Salina Balanceada de Hank (HBSS) con albúmina al 5% y se transportará inmediatamente al Departamento de Fisiología de la Nutrición del INCMNSZ para su procesamiento.  La FEVTA se aislarán del tejido adiposo por el método de separación con colagenasa descrito por Rodbell de la siguiente manera:  El tejido se lavará con solución salina amortiguada por fosfatos (PBSIx) estéril con antibiótico-antimicótico (1%) a 37°C dos veces para eliminar la sangre residual, posteriormente se incubará por 40 min en una solución de colagenasa al 5% en HBSS. Una vez disgregadas, las células se filtrarán en una malla de 100 micras y se centrifugan a 1000 rpm por 5 min para separar las células del estroma vascular de los adipocitos. Se elimina el sobrenadante y las células compactadas se resuspenden en 30 ml de HBSS estéril a 37ºC, se cuantifica la densidad y viabilidad.  Cuarenta ml de grasa aspirada serán colocados en una jeringa de 50 ml y será mantenido en reposo, para decantarla. La sangre y suero que se separen de la grasa, serán eliminados. |
| **31. Información correspondiente para asegurar que las muestras biológicas obtenidas no serán utilizadas para líneas celulares permanentes ni inmortales o fines no relacionados al estudio** |
| Las muestras biológicas obtenidas serán utilizadas en su totalidad para fines de este estudio. En caso de que una parte de estas no se utilice serán desechadas de acuerdo a la normatividad correspondiente. Los investigadores participantes se asegurarán de que las muestras biológicas remanentes sean desechadas. |
| **32. Descripción de los grupos de tratamiento** |
| Grupo control con tratamiento convencional.  Grupo de estudio con tratamiento convencional más aplicación de la fracción vascular estromal del tejido adiposo. |
| **33. Mecanismos para la asignación de los tratamientos** |
| Aleatorización. |
| **34. Si se emplea un grupo con placebo, incluya su justificación** |
| Nuestro estudio no incluye grupo con placebo, incluye un grupo de pacientes que no recibirán la intervención pero que continuarán con tratamiento convencional. Debido a la naturaleza de la intervención (inyección de FEVTA obtenidas de tejido adiposo) no es posible cegar el estudio, ni sería factible ni ético ofrecer una intervención “blanco” (sham) ya que no se justifica someter a liposucción e inyección de alguna solución sin FEVTA en pacientes del grupo control. |
| **35. Criterios para el retiro prematuro del estudio** |
| Solo por deseo del paciente de renunciar al protocolo. |
| **36.Procedimientos para el retiro de un paciente del estudio** |
| Se le informara por escrito mediante una carta, que ya no formará parte del protocolo, se explicara la causa, firmará de conformidad y se le programarán sus citas subsecuentes de acuerdo a la atención médica habitual del instituto. |
| **37. Criterios para la suspensión prematura (parcial o completa) del estudio** |
| Pérdida significativa de pacientes del estudio. |
| **38. Criterios de selección** |
| a) Criterios de inclusión (Deberá incluir la definición de los grupos de edad, sexo y la severidad del padecimiento que serán permitidos en el estudio) |
| 1. Cumplir con el procedimiento de consentimiento informado y firmar la forma de consentimiento informado. 2. Pacientes mayores de edad que hayan cumplido los criterios de Esclerosis Sistémica de ACR 2013, los criterios de LeRoy-Medsger o los de síndrome de CREST (con esclerodactilia y 2 de 4 del resto de los criterios (calcinosis, fenómeno de Raynaud, dismotilidad esofágica y telangiectasias). 3. Presencia de al menos una úlcera digital activa en el momento de la inclusión al estudio. La úlcera debe haber aparecido o empeorado al menos 30 días antes de la inclusión al estudio y debe estar localizada sobre o distal a las articulaciones interfalángicas proximales.    1. Se define úlcera activa como una lesión en el dedo con profundidad discernible a simple vista y con pérdida de la continuidad del epitelio, que está asociada con dolor, y que no se debe a otras causas primarias como infección, artritis, etc. Esta definición no incluye fisuras, paroniquia, cicatrices puntiformes, extrusión de calcio y lesiones indeterminadas (lesiones en las que no se observa claramente denudación de tejido y que no pueden juzgarse, debido a la presencia de una costra o tejido necrótico). 4. Tratamiento vasodilatador estable en las 2 semanas previas a la inclusión al estudio. 5. Tratamiento con estatinas estable en las 4 semanas previas a la inclusión al estudio. 6. Tratamiento inmunomodulador o inmunosupresor estable en las 4 semanas previas a la inclusión del estudio (dosis estable de prednisona o su equivalente, ciclofosfamida, azatioprina, ácido micofenólico, D-penicilamina, metotrexato, cloroquina, hidroxicloroquina, leflunomida o cualquier otro inmunosupresor). 7. Las mujeres en edad fértil deben utilizar 1 método de anticoncepción confiable.    1. Se podrán incluir mujeres en edad fértil con prueba de embarazo previo tratamiento negativa y que utilicen consistentemente y correctamente (desde la visita de selección y hasta 30 días después de terminado el estudio) 1 método anticonceptivo confiable. Los métodos de anticoncepción confiables incluyen: dispositivos intrauterinos, oclusión tubal bilateral, métodos hormonales (anticonceptivos orales combinados o de progesterona sola, parches transdérmicos, anillos vaginales, inyecciones e implantes) y métodos de barrera (preservativo, diafragma o capuchón vaginal). La vasectomía de la pareja y la abstinencia todavía requieren un método anticonceptivo adicional.    2. Se considera que una mujer no es fértil si cumple uno o más de los siguientes criterios:       1. Salpingo-ooforectomía bilateral, oclusión tubaria bilateral o histerectomías previas.       2. Falla ovárica prematura confirmada por un especialista.       3. Síndromes genéticos: Turner, genotipo XY, agenesia uterina.       4. Edad mayor a 50 años, sin tratamiento con terapia hormonal de reemplazo por los 2 años previos a la visita de selección, con amenorrea de al menos 24 meses consecutivos antes de la visita de selección. Puede utilizarse la medición de FSH mayor a 40 UI/L como equivalente de postmenopausia. |
| b) Criterios de exclusión |
| 1. Úlceras digitales secundarias a un padecimiento distinto a ES. 2. Comorbilidades que pudieran afectar seriamente la evaluación de la función de la mano. 3. Mujeres embarazadas, lactando o que planeen embarazarse durante el curso del estudio. 4. Abuso o dependencia de alcohol y/u otras sustancias en los 12 meses previos a la visita de selección. 5. Pacientes con tratamiento vasodilatador (como antagonistas de canales de calcio, IECA, nitroglicerina, bloqueadores alfa adrenérgicos, antagonistas de receptores II de angiotensina, inhibidores de 5 fosfodiesterasa, inhibidores de receptores de endotelina, prostanoides), N-acetilcisteína, tratamiento antiagregante, heparina convencional o de bajo peso molecular, que han recibido este tratamiento por menos de 2 semanas previas a la visita de selección o cuyo tratamiento no ha estado estable por este periodo. 6. Tratamiento inmunomodulador (como prednisona o su equivalente, ciclofosfamida, azatioprina, ácido micofenólico, D-penicilamina, metotrexato, cloroquina, hidroxicloroquina, leflunomida o cualquier otro inmunosupresor) que se haya iniciado en los 3 meses previos a la visita de selección o cuyo tratamiento no haya estado estable por al menos 1 mes previo a la visita de selección 7. Úlceras digitales secundarias a ES infectadas. 8. Uso de factores de crecimiento tópicos u oxígeno hiperbárico concomitante al estudio. 9. Inyección local de toxina botulínica durante el estudio o hasta 4 semanas previas a la visita de inicio. 10. Simpatectomía quirúrgica de extremidades superiores o debridación de la herida quirúrgica dentro del mes previo a la visita de inicio. 11. Tratamiento concomitante con otro medicamento experimental dentro de las 4 semanas previas al estudio o durante el estudio. 12. Índice de masa corporal <18. 13. Cualquier condición que evite que el paciente acuda a las visitas del protocolo o que comprenda la naturaleza del protocolo. 14. Diagnóstico de osteomielitis activa con o sin tratamiento local o sistémico. 15. Antecedente de cáncer en la familia (padres/hermanos/hijos). 16. Presencia de cáncer. 17. Presencia de diabetes mellitus. 18. Presencia de infección por virus de hepatitis B, C o de inmunodeficiencia humana. |
| c) Criterios de eliminación |
| Deseo del paciente de renunciar al protocolo. |
| **39. Desenlaces y variables** |
| **a)** **Variables/desenlaces principales a medir.**   - La variable principal de desenlace será la presencia de eventos adversos.   **b) Variables/desenlaces secundarios a medir.**   1. Se registrarán variables demográficas, así como subtipo clínico de la enfermedad y afección a órganos internos según la escala de severidad de Medsger [^63^](#1rvwp1q). 2. Características de las úlceras: Se evaluarán las siguientes características inmediatamente antes del tratamiento propuesto; después del tratamiento estas se evaluarán cada semana durante las primeras cuatro semanas, y posteriormente mensual hasta los 6 meses.    1. Número de las úlceras.    2. Localización. Esta variable será anotada en una hoja de registro con dibujo de una mano.    3. Dimensiones de las úlceras digitales en forma clínica. Las dimensiones serán tomadas en el eje mayor y eje menor y serán expresadas en milímetros. Para evaluar el desenlace primario se tomará en cuenta la úlcera de mayor tamaño y profundidad, la cual será designada como úlcera índice.    4. Tejidos lesionados. Se registrará si la piel, tejido celular subcutáneo, tendones, articulaciones y/o hueso están afectados.    5. Fotografías clínicas en proyección dorsal y palmar. 3. Dolor. Se realizará una evaluación basada en una escala visual análoga pre-tratamiento, inmediatamente después del tratamiento, cada semana durante las primeras cuatro semanas, y posteriormente mensual hasta los 6 meses 4. Perfusión digital. A cada dedo se le registrará el valor de la oximetría transcutánea con un oxímetro portátil. Tendrá un registro inmediatamente antes del tratamiento, inmediatamente después del tratamiento, una hora después del tratamiento; y posteriormente cada semana durante las primeras cuatro semanas, y posteriormente mensual hasta los 6 meses. 5. Vascularidad del lecho ungueal con un videocapilaroscopio. Se evaluará en la visita del día 0, antes de la administración del tratamiento y en el día 168. Se clasificará según el patrón capilaroscópico en patrón temprano, activo o tardío y se contará el número de capilares por campo.[^64^](#4bvk7pj) 6. Funcionalidad de la mano.    1. Se realizarán los cuestionarios SHAQ (apéndice 1) Y COCHIN (apéndice 2), análoga pre-tratamiento, y a la semana, al mes y a los 6 meses. 7. Mejoría en calidad de vida.    1. Se realizará la evaluación realizando el cuestionario SF-36 análoga pre-tratamiento, y a la semana, al mes y a los 6 meses 8. Efecto inmunomodulador del tratamiento: Cambio del porcentaje de células Th1, Th2, Th17 y Treg en sangre venosa periférica entre el día 0 y el 84. 9. Niveles de marcadores de inflamación y de citocinas en suero en el día 0 y 84. |
| **40. Métodos que serán usados para ponerse en contacto con los pacientes** |
| 1. Citas médicas. 2. Vía telefónica. |
| **41. Análisis estadístico (Descripción del plan de procesamiento y presentación de la información. Incluya la justificación de las pruebas estadísticas que serán usadas)** |
| Se creará una base de datos con las variables demográficas y de las características clínicas de la enfermedad y de la úlcera, en SPSS versión 18. Se utilizará prueba de U de Mann-Whitney para comparar la media del tiempo de curación de la úlcera índice y los otros parámetros numéricos entre los 2 grupos de tratamiento. Las proporciones se compararán con prueba de Chi cuadrada y exacta de Fisher en caso necesario. |
| **42. Justificación del tamaño de muestra (incluya el poder del estudio y el valor de p que será considerado como significativo)** |
| Se trata de un estudio piloto para evaluar la seguridad de la administración de FEVTA en úlceras digitales de pacientes con esclerosis sistémica. No hay estudios previos que permitan calcular adecuadamente el tamaño de muestra por lo que los resultados de este estudio darán información para poder calcular el tamaño de muestra para un estudio en el que se evaluará la eficacia de FEVTA en el tiempo de curación de úlceras digitales en pacientes con ES. |
| **43. Potencial de reclutamiento (número de sujetos que se pretende reclutar)** |
| 20 |
| **44. En caso de ser multicéntrico, incluya el número global y el número local de la muestra** |
| No aplica. |
| **45. Procedimientos para reportar desviaciones del plan estadístico original** |
| No aplica. |
| **46. Molestias posibles resultantes del estudio** |
| - 1. Dolor y datos de inflamación en la zona de liposucción que limitarán sus actividades cotidianas por 3 días.   2. Dolor en la zona de infiltración del lipoinjerto enriquecido con Fracción Vascular Estromal. |
| **47. Riesgos potenciales** |
| 1. Infección del sitio quirúrgico. 2. Infección en la zona de infiltración del lipoinjerto enriquecido con fracción vascular estromal del tejido adiposo. 3. Exacerbación de la isquemia tisular en la mano o dedos tratados. |
| **48. Métodos de detección de riesgos anticipados** |
| Se estudiará la historia clínica de los pacientes que sean considerados para participar en el protocolo. |
| **49. Medidas de seguridad para el diagnóstico oportuno y prevención de los riesgos** |
| 1. Comunicación telefónica con el equipo médico para reporte de sintomatología o signos alarma. (dolor, hiperemia y/o enrojecimiento en el sitio de la liposucción; dolor, hiperemia y/o enrojecimiento en las manos; fiebre; hipotermia y/o palidez digital en la mano). 2. Para evitar la infección el sitio quirúrgico como profilaxis se aplicará una dosis IV de Amoxicilina/Sulbactam 1 gr/500 mg IV o en caso de ser alérgico Ciprofloxacino a dosis de 400 mg IV DU. |
| **50. Procedimientos a seguir para resolver los riesgos en caso de que se presenten** |
| 1. Se indicarán al paciente los datos clínicos de alarma de infección e isquemia, comentándole que acuda dentro de las siguientes 24 horas a consulta con alguno de los cirujanos participantes en el protocolo. En ese caso se le otorgará una consulta gratuita y se le prescribirán los medicamentos necesarios. 2. Antibióticos. 3. Analgésicos. 4. Curaciones. 5. Aplicación de Medicina hiperbárica. |
| **51. Beneficios directos esperados** |
| 1. Disminución del tiempo de curación de la UDES. 2. Disminución del dolor secundario a la presencia de las UDES. 3. Mejoría en la funcionalidad de la mano. 4. Mejoría en la calidad de vida. |
| **52. Beneficios indirectos esperados** |
| Seguimiento estrecho por un equipo multidisciplinario con experiencia en la patología de estos pacientes. |
| **53. Ponderación general de riesgos contra beneficios del estudio propuesto** |
| Los riesgos son previsibles con la correcta selección de los pacientes participantes en el estudio y con la aplicación de antibióticos. La aparición del riesgo más importante es la isquemia digital, la cual sería tratada con Medicina Hiperbárica. Por lo tanto la previsibilidad de las complicaciones y el manejo exitoso de las mismas, versus la disminución en la cicatrización de las úlceras justifica probar esta modalidad de tratamiento. |
| **54. Especifique costos (directos/indirectos, monetarios, en tiempo de participación, visitas/traslados) que la investigación genere para los sujetos del estudio** |
| Los sujetos deberán acudir a 10 consultas de 1 hora cada una aproximadamente, excepto la visita 0 que requerirá aproximadamente 4 horas. Los sujetos no pagarán ninguno de los procedimientos, tratamientos ni consultas. |
| **55. Especifique si las consultas, exámenes de laboratorio/gabinete y tratamientos médicos/quirúrgicos, generados con motivo del estudio serán o no cubiertos por el paciente/sujeto de investigación** |
| Ningún procedimiento, tratamiento ni consulta serán pagados por el paciente. |
| **56. Informe quién cubrirá los costos asociados a la investigación** |
| 1. Departamento de Cirugía del INCMNSZ. Fondos propios del departamento de Cirugía. Proporcionarán los insumos necesarios antes, durante y después del procedimiento de extracción de tejido adiposo y del procedimiento de lipoinjerto enriquecido en células troncales, incluyendo medicamentos y cuidados pre y postquirúrgicos.  2. Departamento de Inmunología y Reumatología del INCMNSZ. Fondos propios de la Dra. Tatiana Rodríguez para toma y procesamiento de las muestras de sangre para evaluar subpoblaciones de linfocitos T y niveles de marcadores de inflamación y citocinas.  3. Departamento de bioquímica del INCMNSZ. Fondos propios del Dr. Alejandro Zentella para reactivos para enriquecimiento del lipoinjerto con células troncales.  4. Departamento de Fisiología de la Nutrición del INCMNSZ. Fondos propios del Dr. Armando Tovar para reactivos para enriquecimiento del lipoinjerto con células troncales. |
| **57. En caso de que corresponda, especifica los incentivos que se ofrecerán (se entiende incentivo como un ofrecimiento o influencia que compele a realizar una acción sin que implique una desviación importante con nuestro plan general de vida; v. gr.: dar un libro por haber participado)**  **Nota: Una compensación/incentivo fuera de proporción se considera una actitud coercitiva** |
| No aplica. |
| **58. Citas bibliográficas**. |
| **Bibliografía**   1. Amanzi L, Braschi F, Fiori G, Galluccio F, Miniati I, Guiducci S, et al. Digital ulcers in scleroderma: staging, characteristics and sub-setting through observation of 1614 digital lesions. *Rheumatology (Oxford)*. 2010 Jul;49(7):1374–82. 2. Galluccio F, Matucci-Cerinic M. Two faces of the same coin: Raynaud phenomenon and digital ulcers in systemic sclerosis. *Autoimmun Rev*. Elsevier B.V.; 2011 Mar;10(5):241–3. 3. Botzoris V, Drosos A. Management of Raynaud’s phenomenon and digital ulcers in systemic sclerosis. *Joint Bone Spine*. 2011 Jul;78(4):341–6. 4. Nitsche A. Raynaud, digital ulcers and calcinosis in scleroderma. *Reumatol Clin*. SEGO; 2012;8(5):270–7. 5. Daumas a, Eraud J, Hautier a, Sabatier F, Magalon G, Granel B. Interests and potentials of adipose tissue in scleroderma. *Rev Med Interne*. Elsevier Masson SAS; 2013 Dec;34(12):763–9. 6. Oh C-K, Lee J, Jang B-S, Kang Y-S, Bae Y-C, Kwon K-S, et al. Treatment of atrophies secondary to trilinear scleroderma en coup de sabre by autologous tissue cocktail injection. *Dermatol Surg*. 2003 Oct;29(10):1073–5. 7. Consorti G, Tieghi R, Clauser LC. Frontal linear scleroderma: long-term result in volumetric restoration of the fronto-orbital area by structural fat grafting. *J* *Craniofac Surg*. 2012 May;23(3): e263–5. 8. Bank J, Fuller SM, Henry GI, Zachary LS. Fat grafting to the hand in patients with Raynaud phenomenon: a novel therapeutic modality. *Plast Reconstr Surg*. 2014 May;133(5):1109–18. 9. Karaaltin MV, Akpinar AC, Baghaki S, Akpinar F. Treatment of “en coup de sabre” deformity with adipose-derived regenerative cell-enriched fat graft*. J Craniofac Surg*. 2012 Mar;23(2): e103–5. 10. Guiducci S, Porta F, Saccardi R, Guidi S, Ibba-Manneschi L, Manetti M, et al. Autologous mesenchymal stem cells foster revascularization of ischemic limbs in systemic sclerosis: a case report. *Annals of internal medicine*. 2010. p. 650–4. 11. Salibian A, Widgerow AD, Abrouk M, Evans GR. Stem cells in plastic surgery: a review of current clinical and translational applications*. Arch Plast Surg*. 2013; 40:666–75 12. Kumar, V. et al., 2010. *Robbins & Cotran Pathologic Basis of Disease*. 13. TrojahnKølle S-F, Oliveri RS, Glovinski P viktor, Elberg JJ, Fischer-Nielsen A, Drzewiecki KT. Importance of mesenchymal stem cells in autologous fat grafting: A systematic review of existing studies. *J PlastSurg Hand Surg*. 2012;46:59–68. 14. Friedenstein AJ, Petrakova KV, Kurolesova AI, Frolova GP. Heterotopic of bone marrow. Analysis of precursor cells for osteogenic and hematopoietic tissues. *Transplantation*.1968; 6:230–47. 15. López-Iglesias, P. et al., 2011. Short and long term fate of human AMSC subcutaneously injected in mice. *World journal of stem cells*, 3(6), pp.53–62. 16. Toyserkani NM, Christensen ML, Sheikh SP, Sørensen JA. Adipose-Derived Stem Cells: New Treatment for Wound Healing? *Ann Plast Surg*. 2014;00. 17. Zhu M, et al. Manual isolation of adipose-derived stem cells from human lipoaspirates. *Journal of Visualized Expermients*. 2013; 79:1-10. 18. Owen M, Friedenstein AJ. Stromal stem cells: marrowderivedosteogenic precursors. *Ciba Found Symp.*1988; 136:42–60. 19. Taupin, P., 2006. Adult neural stem cells, neurogenic niches, and cellular therapy. *Stem cell reviews*, 2, pp.213–219. 20. Watt, F.M., Lo Celso,C. & Silva-Vargas, V. Epidermal stem cells: an update. *Current opinion in genetics & development*.2006;16:518-524 21. Yen, T.H. & Wright, N.A. The gastrointestinal tract stem cell niche. *Stem Cell Rev*. 2006; 2:203-212. 22. Daniels, J.T., Harris, A.R. & Mason, C., 2006. Corneal epithelial stem cells in health and disease. *Stem cell reviews*, 2, pp.247–254. 23. Zuk PA, Zhu M, Mizuno H, Huang J, Futrell JW, Katz AJ, et al. Multilineage cells from human adipose tissue: implications for cell-based therapies. *TissueEng*. 2001;7:211–28. 24. Francis MP, Sachs PC, Elmore LW, Holt E. Isolating adipose-derived mesenchymal stem cells from lipoaspirateblood and saline fraction. *Organogenesis*. 2010;6:11–14. 25. BarretJP, Sarobe N, Grande N, Vila D, Palacin JM. Maximizing results for lipofilling in facial reconstruction. *ClinPlastSurg* 2009; 36:487–92. 26. Tabit CJ, Slack GC, Fan K, Wan DC, Bradley JP. Fat grafting versus adipose-derived stem cell therapy: Distinguishing indications, techniques, and outcomes. *Aesthetic Plast Surg.* 2012;36: 704–13. 27. Gir P, Oni G, Brown S, Mojallal A, Rohrich RJ. Human adipose stem cells: current clinical applications. *PlastReconstr Surg*. 2012;129:1277–90. 28. Fang B, Song YP, Li N, Li J, Han Q, Zhao RC. Resolution of refractory chronic autoimmune thrombocytopenic purpurafollowing mesenchymal stem cell transplantation: A case report. *Transplant Proc.* 2009;41:1827–1830. 29. RaJC, Shin IS, Kim SH, Kang SK, Kang BC, Lee HY, et al. Safety of intravenous infusion of human adipose tissue-derived mesenchymal stem cells in animals and humans. *Stem Cells Dev*. 2011; 20:1297–308. 30. Yoshimura K, Sato K, Aoi N, Kurita M, Inoue K, Suga H, et al. Cell-assisted lipotransfer for facial lipoatrophy: Efficacy of clinical use of adipose-derived stem cells. *Dermatologic Surg*. 2008; 34:1178–85. 31. TiryakiT, Findikli N, Tiryaki D. Staged stem cell-enriched tissue (SET) injections for soft tissue augmentation in hostile recipient areas: A preliminary report. *Aesthetic Plast Surg*. 2011; 35:965–71. 32. Castro-Govea, Y. et al., 2012. Cell-assisted lipotransfer for the treatment of parry-romberg syndrome. *Archives of plastic surgery*, 39(6), pp.659–62. 33. Yoshimura K, Sato K, Aoi N, Kurita M, Hirohi T, Harii K. Cell-assisted lipotransfer for cosmetic breast augmentation: Supportive use of adipose-derived stem/stromal cells. *Aesthetic Plast Surg*. 2008; 32:48–55. 34. Kamakura T, Ito K. Autologous cell-enriched fat grafting for breast augmentation. *Aesthetic Plast Surg*. 2011;35:1022–1030. 35. Kim M, Kim I, Lee SK, Bang SI, Lim SY. Clinical trial of autologous differentiated adipocytes from stem cells derived from human adipose tissue*Dermatol Surg*. 2011;37:750–9. 36. Hassan WU, Greiser U, Wang W. Role of adipose-derived stem cells in wound healing. *Wound Repair Regen*. 2014 May; 22(3):313–25. 37. NaeS, Bordeianu I, Stăncioiu AT, Antohi N. Human adipose-derived stem cells: definition, isolation, tissue-engineering applications. *Rom J MorpholEmbryol*. 2013;54:919–24. 38. Matsuda K, Falkenberg KJ, Woods AA, Choi YS, Morrison WA, Dilley RJ. Adipose-Derived Stem Cells Promote Angiogenesis and Tissue Formation for In Vivo *Tissue Engineering. Tissue Eng Part A*. 2013;19:1327–35. 39. BarbaM, Cicione C, Bernardini C, Michetti F, Lattanzi W. Adipose-derived mesenchymal cells for bone regereneration: state of the art. *Biomed Res Int*. 2013 Jan;2013:416391. 40. Wu L, Cai X, Zhang S, Karperien M, Lin Y. Regeneration of articular cartilage by adipose tissue derived mesenchymal stem cells: perspectives from stem cell biology and molecular medicine. *J Cell Physiol*. 2013 May;228(5):938–44. 41. Euler de Souza Lucena E, Guzen FP, Lopes de Paiva Cavalcanti JR, Galvão Barboza CA, Silva do Nascimento Júnior E, Cavalcante JDS. Experimental considerations concerning the use of stem cells and tissue engineering for facial nerve regeneration: a systematic review*. J Oral Maxillofac Surg*. 2014 May;72(5):1001–12. 42. NambuM, Kishimoto S, Nakamura S, Mizuno H, Yanagibayashi S, Yamamoto N, et al. Accelerated wound healing in healing-impaired db/db mice by autologous adipose tissue-derived stromal cells combined with atelocollagen matrix. *Ann Plast Surg*. 2009;62:317–21. 43. Amos PJ, Kapur SK, Stapor PC, et al. Human adipose-derived stromal cellsaccelerate diabetic wound healing: impact of cell formulation and delivery.*Tissue Eng Part A*. 2010;16:1595Y1606. 44. Di Rocco G, Gentile A, Antonini A, Ceradini F, Wu JC, Capogrossi MC, et al. Enhanced healing of diabetic wounds by topical administration of adipose tissue-derived stromal cells overexpressing stromal-derived factor-1: biodistribution and engraftment analysis by bioluminescent imaging. *Stem Cells Int*. 2010; 2011:304562. 45. MaharlooeiMK, Bagheri M, Solhjou Z, Jahromi BM, Akrami M, Rohani L, et al. Adipose tissue derived mesenchymal stem cell (AD-MSC) promotes skin wound healing in diabetic rats. *Diabetes Res ClinPract*. 2011; 93:228–34. 46. Nie C, Yang D, Xu J, Si Z, Jin X, Zhang J. Locally administered adipose-derived stem cells accelerate wound healing through differentiation and vasculogenesis. *Cell Transplant*. 2011; 20:205–16. 47. Nie C, Zhang G, Yang D, et al. Targeted delivery of adipose-derived stem cells via acellular dermal matrix enhances wound repair in diabetic rats. *J Tissue EngRegen Med*. 2012. 48. Kim EK, Li G, Lee TJ, Hong JP. The effect of human adipose-derived stem cells on healing of ischemic wounds in a diabetic nude mouse model.*PlastReconstr Surg*. 2011;128:387–94. 49. EbrahimianTG, Pouzoulet F, Squiban C, et al. Cell therapy based on adipose tissue derived stromal cells promotes physiological and pathological wound healing. *ArteriosclerThrombVasc Biol*. 2009; 29:503Y510. 50. TsumanoT, Kawai K, Ishise H, Nishimoto S, Fukuda K, Fujiwara T, et al. A new mouse model of impaired wound healing after irradiation.*J PlastSurg Hand Surg*. 2013;47:83–8. 51. SteinbergJP, Hong SJ, Geringer MR, Galiano RD, Mustoe TA. Equivalent Effects of Topically-Delivered Adipose-Derived Stem Cells and Dermal Fibroblasts in the Ischemic Rabbit Ear Model for Chronic Wounds.*AesthetSurg J*. 2012;32:504–19. 52. Hadad I, Johnstone BH, Brabham JG. Development of a porcine delayed wound healing model and its use in testing a novel cell-based therapy. *Int J Radiat Oncol Biol Phys*. 2010; 78:888Y896. 53. Zhu M, Zhou Z, Chen Y, et al. Supplementation of fat grafts with adipose-derived regenerative cells improves long-term graft retention. *Ann Plast Surg.* 2010; 64:222–8. 54. LinYC, Grahovac T, Oh SJ, Ieraci M, Rubin JP, Marra KG. Evaluation of a multi-layer adipose-derived stem cell sheet in a full-thickness wound healing model*. ActaBiomater*. 2013; 9:5243–50. 55. CianfaraniF, Toietta G, Di Rocco G, Cesareo E, Zambruno G, Odorisio T. Diabetes impairs adipose tissue-derived stem cell function and efficiency in promoting wound healing. *Wound Repair Regen*. 2013; 21:545–53. 56. El-Ftesi S, Chang EI, Longaker MT, Gurtner GC. Aging and diabetes impair the neovascular potential of adipose-derived stromal cells. *PlastReconstr Surg*. 2009; 123:475–85. 57. Yoshimura, K. et al. Ectopic fibrogenesis induced by transplantation of adipose-derived progenitor cell suspension immediately after lipoinjection. *Transplantation*.2008;85:12:1868–9. 58. RussoFP, Alison MR, Bigger BW, Amofah E, Florou A, Amin F, et al. The Bone Marrow Functionally Contributes to Liver Fibrosis. Gastroenterology. 2006; 130:1807–21. 59. Yoshimura, K. et al. *In vivo* Manipulation of stem cells for adipose tissue repair/reconstruction. *Regenerative Medicine*.2011;6:33–41. 60. DonnenbergVS, Zimmerlin L, Rubin JP, Donnenberg AD. Regenerative therapy after cancer: what are the risks? *Tissue Eng Part B Rev*. 2010; 16:567–75. 61. Ley General de Salud. Estados Unidos Mexicanos. Diario Oficial de la Federación 7 de febrero de 1984. Última reforma publicada en el Diario Oficial de la Federación el 15 de enero del 2014. 62. Rodríguez-Reyna TS, Furuzawa J, Cabiedes J. et al. Th17 peripheral cells are increased in diffuse cutaneous systemic sclerosis compared with limited illness: a cross-sectional study. *Rheumatol Int*. 2012;32(9):2653-60. 63. Medsger TA Jr, Bombardieri S, Czirjak L, et al. Assessment of disease severity and prognosis. Clin Exp Rheumatol. 2003; 21 (Suppl 29):S60-S64. 64. Maricq HR, Spencer-Green G, LeRoy EC. Skin capillary abnormalities as indicators of organ involvement in scleroderma (systemic sclerosis), Raynaud’s syndrome and dermatomyositis. *Am J Med*. 1976; 61:862-70. 65. Fang B, Song YP, Liao LM, Han Q, Zhao RC. Treatment of severe therapy-resistant acute graft-versus-host disease with human adipose tissue-derived mesenchymal stem cells. *Bone Marrow Transplant*. 2006; 38:389–390. 66. Fang B, Song Y, Zhao RC, Han Q, Lin Q. Using human adipose tissue-derived mesenchymal stem cells as salvage therapy for hepatic graft-versus-host disease resembling acute hepatitis. *Transplant Proc*. 2007; 39:1710–1713. 67. Fang B, Song YP, Li N, Li J, Han Q, Zhao RC. Resolution of refractory chronic autoimmune thrombocytopenic purpura following mesenchymal stem cell transplantation: A case report. *Transplant Proc*. 2009; 41:1827–1830. 68. Fang B, Mai L, Li N, Song Y. Favorable response of chronic refractory immune thrombocytopenic purpura to mesenchymal stem cells. *Stem Cells Dev*. 2012; 21:497–502. 69. Fang B, Song Y, Li N, Li J, Han Q, Zhao RC. Mesenchymal stem cells for the treatment of refractory pure red cell aplasia after major ABO-incompatible hematopoietic stem cell transplantation. *Ann Hematol*. 2009; 88:261–266. 70. Trivedi HL, Vanikar AV, Thakker U, et al. Human adipose tissue-derived mesenchymal stem cells combined with hematopoietic stem cell transplantation synthesize insulin. *Transplant Proc*. 2008; 40:1135–1139. 71. Vanikar AV, Dave SD, Thakkar UG, Trivedi HL. Cotransplantation of adipose tissue-derived insulin-secreting mesenchymal stem cells and hematopoietic stem cells: A novel therapy for insulin-dependent diabetes mellitus. *Stem Cells Int*. 2010; 2010:582382. 72. Garcia-Olmo D, Herreros D, Pascual M, et al. Treatment of enterocutaneous fistula in Crohn’s Disease with adipose-derived stem cells: A comparison of protocols with and without cell expansion. *Int J Colorectal Dis*. 2009; 24:27–30. 73. Garcia-Olmo D, Garcia-Arranz M, Herreros D. Expanded adipose-derived stem cells for the treatment of complex perianal fistula including Crohn’s disease. *Expert Opin Biol Ther*. 2008; 8:1417–1423. 74. Garcia-Olmo D, Herreros D, Pascual I, et al. Expanded adipose- derived stem cells for the treatment of complex perianal fistula: A phase II clinical trial. *Dis Colon Rectum.* 2009; 52:79–86. 75. Alvarez PD, Garcı´a-Arranz M, Georgiev-Hristov T, Garcı´a- Olmo D. A new bronchoscopic treatment of tracheomediastinal fistula using autologous adipose-derived stem cells. *Thorax* 2008; 63:374–376. 76. Ichim TE, Harman RJ, Min WP, et al. Autologous stromal vascular fraction cells: A tool for facilitating tolerance in rheumatic disease. *Cell Immunol*. 2010; 264:7–17. 77. Riordan NH, Ichim TE, Min WP, et al. Non-expanded adipose stromal vascular fraction cell therapy for multiple sclerosis. *J Transl Med*. 2009; 7:29. 78. Mesimäki K, Lindroos B, Törnwall J, et al. Novel maxillary reconstruction with ectopic bone formation by GMP adipose stem cells. *Int J Oral Maxillofac Surg*. 2009; 38:201–209. 79. Taylor JA. Bilateral orbitozygomatic reconstruction with tissue- engineered bone. *J Craniofac Surg*. 2010;21:1612–1614. 80. Lendeckel S, Jödicke A, Christophis P, et al. Autologous stem cells (adipose) and fibrin glue used to treat widespread traumatic calvarial defects: Case report*. J Craniomaxillofac Surg*. 2004;32:370–373. 81. Pak J. Regeneration of human bones in hip osteonecrosis and human cartilage in knee osteoarthritis with autologous adipose-tissue-derived stem cells: A case series. *J Med Case Reports* 2011;5:296. 82. Yamamoto T, Gotoh M, Hattori R, et al. Periurethral injection of autologous adipose-derived stem cells for the treatment of stress urinary incontinence in patients undergoing radical prostatectomy: Report of two initial cases. *Int J Urol*. 2010; 17:75–82. 83. Van Laar JM, Farge D, Sont JK et al. Autologous hematopoietic stem cell transplantation vs intravenous pulse cyclophosphamide in diffuse cutaneous systemic sclerosis: a randomized clinical trial. JAMA 2014; 311(24):2490-8. |

| **Tabla 1. Perfil de expresión de Células Troncales Derivadas de Tejido Adiposo Humano en la superficie celular** | | |
| --- | --- | --- |
|  | Perfil de expresión | |
|  | Expresión Positiva | Expresión Negativa |
|  | CD9, **CD10, CD13, CD29, CD34, CD44**, CD49a, **CD49d,** CD49e  CD51, CD54, CD55, CD59, CD61, CD63, CD71, CD73, CD90, CD105, CD138, CD140a, CD146, CD166, HLA-ABC, STRO-1 | **CD11a, CD11b, CD11c, CD14,** CD16, CD18, **CD31,** CD41a, CD49f, **CD45,** CD50, CD56,  CD62e, CD62l, CD62P, CD104, **CD106**, CD133, **CD144, CD146, HLA-DR, SMA,** ABCG2 |
| **Fenotipo propuesto de CTDTA**  **(común entre 2 o más estudios)** | **CD10, CD13, CD29, CD34, CD44, CD49d, CD54, CD90, CD140a**    **HLA-ABC, STRO-1** | **CD11a, CD11b, CD11c, CD14, CD31, CD45, CD106, CD144** |
| **Marcadores controversiales en CTDTA** | CD105, CD117, CD140b, CD146, CD166, SMA, HLA-DR | |
| **Marcadores de células estromales** | CD29, CD44, CD73, CD90, CD166 | |
| **Marcadores hematopoyéticos** | CD31, CD34, CD45, ABCG2 | |
| **Marcadores en común entre dos o más estudios marcado en negritas.**  **SMA, actina de musculo liso; HLA, antígeno leucocitario humano: ABCG2, proteína transportadora de múltiples fármacos G2**  **Tomada de: Zuk P. et al. Multilineage cells from human adipose tissue: implications for cell-based therapies. TissueEng. 2001; 7:211–28. Traducida al español.** | | |

| **Tabla 2. Aplicaciones clínicas de las células troncales derivadas de tejido adiposo en otras especialidades** | | | |
| --- | --- | --- | --- |
| **Especialidades** | **Referencias** | **No. De pacientes tratados** | **Dosis de CTDTA y administración** |
| **Trastornos hematológicas e inmunológicas** | Fang et al. | 14 | 1-2x10^6^ CTDTA alogénicas/kg, IV |
| **Diabetes mellitus** | Trivedi et al. | 5 | 3.15x10^6^ CTDTA alogénicas inyectadas por infusión  intraportal bajo anestesia general mediante  minilaparotomía |
| **Enfermedades digestivas** | Vanikar et al.  Garcia-Olmo et al. | 11  63 | 3x10^6^ a 2x10^7^ CTDTA autólogas dentro de la fístula |
| **Enfermedades autoinmunes** | Ichim et al.  Riordan et al. | 1  3 | 53x10^6^ PVETA autólogo en dos infusiones IV  25-75x10^6^ PVETA autólogo IV |
| **Fístula traqueomediastinal** | Alvarez et al. | 1 | 4.9x10^6^ PVETA autólogo dentro de la cavidad de la  fístula |
| **Reparación de tejido óseo** | Lendeckel et al.  Medimäki et al.  Taylor  Pak | 1  1  1  4 | 295x10^6^ PVETA autólogo  13x10^6^ CTDTA autólogas  28 ml de fracción sólida de lipoaspirado autólogo fresco  10 cm^3^ PVETA autólogo |
| **Trastornos urológicos** | Yamamoto et al. | 2 | 2.4-3.2x10^7^ PVETA autólogo dentro del esfínter uretral |
| **Enfermedad neurológica** | Ra et al. | 8 | 4x10^8^ CTDTA autólogas IV |
| **Total** |  | 115 |  |
| **CTDTA, Células troncales derivadas de tejido adiposo; IV, Intravenoso; PVETA, Porción vascular estromal de tejido adiposo.**  **Tomada de: Gir P, et al. Human adipose stem cells: current clinical applications. Plast. Reconstr. Surg. 2012; 129:1277–90. Traducida al español.** | | | |

| **Tabla 3. Aplicaciones Clínicas de Células Troncales Derivadas de Tejido Adiposo en Cirugía Plástica** | | |
| --- | --- | --- |
| **Referencias** | **No. de pacientes tratados** | **Dosis de CTDTA y administración** |
| **Aumento de tejidos blandos** |  |  |
| **Yoshimura et al.**  **Yoshimura et al.**  **Yoshimura et al.**  **Tiryaki et al.**  **Kamakura e Ito**  **Kim et al.** | 15  40  29  20  31 | 263.5 ml de grasa enriquecida con PVETA inyectada en cada seno  272.7 ml de grasa enriquecida con PVETA inyectada en cada seno  133 ml de grasa inyectada en el grupo no-TGCA, 100 ml de grasa enriquecida con PVETA en el grupo TGCA  10-390ml de grasa enriquecida con PVETA (TGCA) por admin local  240ml de grasa enriquecida con PVETA en cada seno (TGCA)  0.11-4.63x10^7^ CTDTA autólogas en cada cicatriz |
| **Cicatrización** |  |  |
| **Rigotti et al.**  **Akita et al.** | 20  1 | 7.4±3.6x10^5^ PVETA autóloga en cada lesión (60-80ml de tejido graso)  3.8x10^7^ PVETA autóloga dentro de cada lesión |
| **Ingeniería de tejidos** |  |  |
| **Stillaert et al.** | 12 | 0.67-1.4x10^6^ CTDTA por andamio |
| **Total** | 174 |  |
| **CTDTA, células troncales derivadas de tejido adiposo; PVETA, porción vascular estromal de tejido adiposo; TGCA, trasplante de grasa celular asistida.**  **Tomada de: Gir P, et al. Human adipose stem cells: current clinical applications. *PlastReconstr Surg*. 2012; 129:1277–90. Traducida al español.** | | |
