# Supplementary material for: Adipose derived stromal vascular fraction and fat graft for treating the hands of patients with systemic sclerosis. A randomized clinical trial
Source: PLoS One. 2023 Aug 14;18(8):e0289594. doi: 10.1371/journal.pone.0289594 (PMC10424873; doi:10.1371/journal.pone.0289594)
Supplement: S2 File — (DOCX) [file pone.0289594.s003.docx]

|  | **Research Ethics Committee/Research Committee** | Code |
| --- | --- | --- |
|  |  | Rev. 1 |
|  | Request for evaluation of research protocols | Sheet: 1 de 31 |

Registration Number CIIBH: SCI-1505-15/15-1

| **1. Project Title** |
| --- |
| Safety of treatment of refractory ischemic digital ulcers due to systemic sclerosis with lipo-graft enriched with adipose derived stromal vascular fraction. |
| **2. Protocol number and version (include version date)** |
| Number: 1  Version 1 Date Jan 19th, 2015 |
| **3.Research Type** |
| \| **Research Type** \| **Option** \| \| --- \| --- \| \| Pharmacology \|  \| \| Biomedical \| **x** \| \| Epidemiological \|  \| \| Interchangeability \|  \| \| Other \|  \| |
| **4. Researchers**  **4a. Identification**   \| **RESEARCHERS** \| **Institutional Position** \| **Roll in the project** \| **Phone (ext.)** \| **E-mail** \| \| --- \| --- \| --- \| --- \| --- \| \| Dr. Martín Iglesias Morales \| Head of Plastic Surgery Service at Instituto Nacional de Ciencias Médicas y Nutrición “Salvador Zubirán” INCMNSZ \| Principal Researcher \| 2140 \| iglesias@drmartiniglesias.com \| \| Dra. Magda Patricia Butrón Gandarillas \| Plastic Surgery Service INCMNSZ \| Associate Researcher \| 2140 \| butronpaty@yahoo.com.mx \| \| Dr. Francisco Javier Pineda Gutiérrez \| Fellow Clerk in Plastic Surgery Service at INCMNSZ \| Associate Researcher \| 2140 \| fjpinedagu@gmail.com \| \| Dr. Armando Roberto Tovar Palacio \| Head Physiology of Nutrition Department at INCMNSZ \| Associate Researcher \| 2809 \| tovar.ar@gmail.com \| \| Dra. Tatiana Sofía Rodríguez Reyna \| Immunology and Rheumatology Service at INCMNSZ \| Associate Researcher \| 2603 \| sofarodriguez@yahoo.com.mx \| \| Dr. Alejandro Zentella Dehesa \| Head of Biochemistry at INCMNSZ \| Associate Researcher \| 4351 \| azentell@biomedicas.unam.mx \| \| Dr. Iván Torre Villalvazo \| Physiology of Nutrition Department at INCMNSZ \| Associate Researcher \|  \| ivan.inn@gmail.com \| \| Dr. Mario Arturo Morán Romero \| Fellow Clerk in Plastic Surgery Service at INCMNSZ \| Associate Researcher \|  \| marioarturom@gmail.com \| \| Dr. Ángel Uriel Cruz Reyes \| Fellow Clerk in Plastic Surgery Service at INCMNSZ \| Associate Researcher \|  \| drangelcr90@gmail.com \| \| Alan Miguel Hernández Campos \| Fellow Clerk in Plastic Surgery Service at INCMNSZ \| Associate Researcher \|  \| dr.alan.campos.hdz@gmail.com \| \| Kenia Paulina Zaragoza Cortés \| Fellow Clerk in Plastic Surgery Service at INCMNSZ \| Associate Researcher \|  \| keniia_zc@hotmail.com \| \| Estefanía Alatorre Vázquez \| Fellow Clerk in Plastic Surgery Service at INCMNSZ \| Associate Researcher \|  \| estefalatorre@gmail.com \| |
| **4b. Relevance of the group of researchers with respect to the project** |
| \| **Research** \| **Member of the National Research System** \| **Experience in research studies** \| \| --- \| --- \| --- \| \| Dr. Martin Iglesias Morales \| 2 \| Head of the Plastic Surgery Service of the INCMNSZ. Surgeon specialized in Plastic and Reconstructive Surgery; subspecialty of Reconstructive Microsurgery and Hand Surgery; member of the National System of Researchers level II; member of the National Academy of Medicine; member of the Mexican Academy of Surgery, member of International Hand and Composite Tissue Transplantation. Professor of the highly specialized course in Rheumatic Hand Surgery of UNAM at the INCMNSZ, Tutor of the Research program of AFINES system of UNAM. Associate Professor at School of Medicine of Instituto Tecnológico y Estudios Superiores de Monterrey, México \| \| Dra. Magda Patricia Butrón Gandarillas \|  \| Plastic Surgery Service of the INCMNSZ. Surgeon with extensive knowledge in relation to Reconstructive Surgery, Microsurgery and Hand Surgery. Member of SCOTTCO and the Mexican Association of Aesthetic and Reconstructive Plastic Surgery. \| \| Dr. Armando Roberto Tovar Palacio \| 3 \| Researcher in Medical Sciences. Dr. Tovar has extensive experience in the analysis and writing of basic research articles and will participate in the analysis and discussion of the results and the writing of the manuscripts. \| \| Dra. Tatiana Sofía Rodríguez Reyna \| 1 \| Researcher in Medical Sciences D. Rheumatologist, in charge of the INCMNSZ cohort of patients with Systemic Sclerosis, which currently consists of 260 patients with annual follow-up. Dr. Rodríguez has training and experience in caring for patients with systemic sclerosis and its complications. \| \| Dr. Alejandro Zentella Dehesa \| 2 \| Head of Department of Biochemistry of the INCMNSZ \| \| Dr. Iván Torre Villalvazo \|  \| Researcher in Medical Sciences C. Dr. Torre has experience in obtaining and primary culture of cells and will participate in the procedure for isolating precursor cells from the vascular stroma of patients, will perform gene expression analysis by real-time PCR and will participate in the discussion of results and the writing of the manuscripts. \| |
| **5. Participant Institutions** |
| \| **Institution**  **(Company name and address)** \| **Role you will play in the project** \| **Did you give approval to the project?** \| \| --- \| --- \| --- \| \| Instituto Nacional de Ciencias Medicas y Nutricion “Salvador Zubiran” Vasco de Quiroga 15, Colonia Sección XVI, Tlalpan C.P.14000, México D.F., MEXICO \| Principal Research. protocol design, follow-up, execution, financing, analysis and publication of results \|  \| |
| **6. Sponsorship**  **6a. Sponsoring Organizations** |
| 1. INCMNSZ Department of Surgery. Own funds of the Department of Surgery. They will provide the necessary supplies before, during and after the adipose tissue extraction procedure and the ADSVF enriched fat grafting procedure, including medications and pre- and post-surgical care.  2. INCMNSZ Department of Immunology and Rheumatology. Own funds dedicated to research, from Dr. Tatiana Rodríguez for taking and initial processing of blood samples.  3. INCMNSZ Department of Biochemistry. Own funds of Dr. Alejandro Zentella for reagents for enrichment of the lipo-graft with ADSVF.  4. Department of Physiology of Nutrition of the INCMNSZ. Own funds of Dr. Armando Tovar for reagents for enrichment of the lipo-graft with ADSVF. |
| **6b. Specify whether researchers receive payment (monetary or in-kind) for their specific participation in the research.** |
| The researchers do not receive any kind of monetary payment for their participation in this research. |
| **7. Abstract (Limit 400 words)** |
| The compromise of the microcirculation caused by Systemic Sclerosis (SSc) produces digital lesions, characterized by ulcers, punctate scars, digital necrosis and calcinosis; digital ulcers are a frequent complication that causes significant disability and complications such as infections and amputation of the tissue involved. The ulcers are usually persistent, difficult to manage, extremely painful, can cause tissue loss and damage to the functions of the hand. They directly impact the quality of life of the patient, generating a greater risk for the development of infections (gangrene, osteomyelitis and septicemia) and amputations, a prolonged healing period and a high economic cost. Its reported average healing time is 76.2 days (minimum 7 days, maximum 810 days) carrying out the treatment based on continuous healing, vasodilators, analgesics and, if necessary, antibiotics.  The application of adipose derived stromal vascular fraction (ADSVF) has shown faster healing in wounds with circulatory compromise. Therefore, the objective of this protocol is to evaluate the safety of ADSVF transplantation in patients with digital ulcers secondary to SSc. Since there is no information on the use of ADSVF in this pathology, carrying out this pilot study will allow us to obtain measurements of the efficacy of ADSVF in the healing time of the DUs in order to carry out a subsequent study with a sample size calculation focused on effectiveness.  The study will be prospective, longitudinal, single center, randomized, and unblinded. 10 patients with digital ulcers will be a control group and in 10 patients with digital ulcers the ADSVF will be applied distributed on the back and palm of the hand and fingers. The Department of Plastic Surgery will perform the extraction of fat through liposuction, which will later be processed in the Department of Physiology of Nutrition for the extraction of ADSVF, to later apply the ADSVF plus lipo-graft in the regions described above.  The follow-up will be 6 months and the following will be evaluated: regional circulation, improvement in pain with a visual analogue scale and hand function with the COCHIN and SHAQ scales, improvement in quality of life with the SF-36 questionnaire. and the immunomodulatory effect with the detection of the subpopulations of regulatory T and B cells.  A database will be created with the demographic variables and the clinical characteristics of the disease and the ulcer, in SPSS version 18. The Mann-Whitney U test will be used to compare the mean healing time of the index ulcer between the 2 treatment groups. The proportions will be compared with the Chi-square test. |
| **8. Background** |
| Systemic sclerosis (SSc) is an autoimmune disease that affects connective tissue, arterioles, and microcirculation. It is characterized by the presence of functional and structural microvasculopathy that results in ischemia and, on the other hand, by the appearance of cutaneous and visceral fibrosis. The initial vascular disease is characteristically evidenced by Raynaud's phenomenon and by the presence of microvascular alterations observed in capillaroscopy, even years before the appearance of other signs of the disease. ^1-^[^5^](#2et92p0)  Microcirculation compromise, generally manifested by Raynaud's phenomenon in SSc, causes digital lesions such as skin infarcts that leave punctate scars, as well as ulcers and gangrene in the fingertips, with digital ulcers being one of the most frequent and refractory complications to treatment. digital ulcers are persistent, difficult to manage, extremely painful, can cause tissue loss, self-amputation, and damage to hand functions. They also have a direct impact on the patient's quality of life, generating a greater risk for the development of infections (gangrene, osteomyelitis and septicemia) and amputations. [^1^](#gjdgxs)^,^ [^2^](#30j0zll)^,^ [^3^](#1fob9te)  The etiology of digital ulcers is multifactorial, but vasospasm, vasculopathy, intraluminal platelet activation, thrombosis, repetitive trauma, xerosis, and skin thinning contribute to their development and perpetuation. [^2^](#30j0zll), [^4^](#3znysh7)  Digital ulcers occur in about 30-40% of patients with SSc. The most common location is the tips of the fingers and over bony prominences.[^3^](#1fob9te) Ulcers are seen in both diffuse and limited SSc patients. [^4^](#3znysh7)  An active ulcer is considered one whose evolution is less than 3 months, and chronic, if it is greater than 3 months. 30% of patients with digital ulcers present loss of soft tissue and bone. Up to 12% of patients require hospitalization and surgery. From 43-58% will develop at least one digital ulcer at some point in the disease. In 31.8-71.4% of cases they will become chronic and persistent ulcers. Twenty-five percent of patients with digital ulcers present with more than two digital ulcers, and between 14-29% of cases progress to gangrene and autoamputation.[^1^](#gjdgxs)^,^ [^4^](#3znysh7)  The study by Amanzi et al showed the characteristics of 1614 digital ulcers in 100 patients over a period of 4 years. The average number of digital ulcers per patient was 15.7. The distribution of the digital ulcers by hand was 55% right hand and 45% left hand. The digital ulcers were located more frequently in the second (24.5%) and third (26.6%) fingers. 52% of the digital ulcers were located at the tip of the finger, 30% in the dorsal region of the fingers, 13% in the nail area and very rarely in the palmar region. The average healing time was 76.2 days (minimum 7 days, maximum 810 days) using conventional treatment.[^1^](#gjdgxs)  The impact on the quality of life and disability of patients with digital ulcers can be objectified through the Health Assessment Questionnaire (HAQ), the visual pain scale, the ulcer severity scale, the loss of function of the involved limb and loss of function of the joint directly compromised by the ulcer. [^4^](#3znysh7)  Current management for the treatment and prevention of Raynaud's phenomenon and digital ulcers includes calcium channel blockers (first-line treatment), angiotensin II receptor antagonists, antiplatelet agents, endothelin receptor antagonists (reduce the number of new lesions especially in patients at risk of multiple ulcers, but no effect on healing has been found), 5-phosphodiesterase inhibitors (show significant benefit in terms of frequency, duration and severity of attacks), statins (show efficacy in the reduction of the appearance and decrease in the total number of digital ulcers, improvement in Raynaud's phenomenon as well as in the damage of vascular function) and prostaglandin analogues. [^2^](#30j0zll) The use of anticoagulants may be considered in cases of acute ischemia or when a thrombotic complication is suspected.  The last therapeutic option is surgical management. Surgical options include arterial bypass, digital artery reconstruction, sympathectomy (peripheral and digital), and botulinum toxin injection. These therapeutic options are only reserved for patients with digital ulcers refractory to medical treatment or in patients with intractable pain.[^2^](#30j0zll) Conventional medical-surgical treatment consists of disease control and serial healing in order to prevent infection until secondary healing of the ulcer is achieved.  **Stem Cells Derived from Adipose Tissue and Vascular Stromal Fraction of Adipose Tissue**  Stem cells are an undifferentiated cell population, they have the ability to self-renew or differentiate into other cell types. These cells are of essential importance for regenerative medicine, which raises the possibility of repairing or replacing diseased cells, tissues and/or organs, through the transplantation of healthy cells and tissues. [^11^](#17dp8vu)  Stem cells are maintained throughout the life of the organism. This process is carried out by two mechanisms:   1. Obligatory asymmetric replication: with each cell division one of the daughter cells retains the capacity for self-renewal, that is, they continue to be stem cells, while the other daughter cell enters a differentiation pathway. 2. Stochastic differentiation: A population of stem cells is maintained by the balance between stem cell divisions that generate either two self-renewing stem cells or two daughter cells that will enter a differentiation pathway.   Embryonic stem cells (ESCs) have the highest regenerative capacity, are pluripotent, and can differentiate into all body tissues. Pluripotent stem cells will give rise to multipotent stem cells with more limited differentiation capacity, which in turn will give rise to differentiated cells of the three embryonic layers (ectoderm, mesoderm, and endoderm). [^12^](#3rdcrjn)^,^ [^11^](#17dp8vu)  Adult stem cells, such as mesenchymal stem cells (MSCs), were described by Friedenstein et al. in the 1960s, [^13^](#26in1rg)^,^ [^14^](#lnxbz9) they reside in places with a special microenvironment which is called a niche. Niche cells generate stimuli that regulate stem cell self-renewal and the generation of offspring cells. [^12^](#3rdcrjn) MSCs can give rise to different tissues such as cartilage, bone, skin, bone marrow, adipose tissue, muscle, epithelial cells, and neuronal cells. Within this group of cells are bone marrow stromal cells (BMSC), adipose-derived stem cells (ASC) or adipose-derived stromal cells named in this way by the International Society Applied to Fatty Tissue. [^13^](#26in1rg) and stromal cells of the skin. All of these cells are multipotent and are restricted to differentiating into the cell lines of the locations where they reside. [^11^](#17dp8vu)^,^ [^15^](#35nkun2)^,^ [^16^](#1ksv4uv)  According to the International Society for Cellular Therapy, the minimum criteria to define a cell as MSCs is the expression of the following markers on its cell membrane: CD73, CD90 and CD105 with the absence of the following membrane markers: CD34, CD45, CD14 or CD11b, CD79α or CD19 and HLA-DR and have the ability to differentiate into specialized cells derived from mesoderm in vitro.[^13^](#26in1rg) However, in the review by Patricia Zuk, ASC cells are positive for CD13, CD29, CD44, CD49, CD90, and CD105; and negative for CD14, CD31, CD45 and CD144 [^17^](#44sinio) ([Table 1](#1opuj5n))  Adult stem cells divide very slowly in most tissues, these cells generate other rapidly dividing cells (in-transit amplifying cells) and give rise to progenitor cells which, in turn, will give rise to stem cells. differentiated cells that will form part of the resident cells of the organ where the differentiation took place.  Some of the places where the presence of adult stem cells has been studied are: bone marrow, liver, brain, skin, intestinal epithelium, cardiac and skeletal muscle, cornea, and adipose tissue.  In the bone marrow there are two types of stem cells, the hematopoietic stem cells that give rise to all blood cell lines and the bone marrow stromal cells (BMSC) which are multipotent and can give rise to osteocytes, chondrocytes, osteoblasts, adipocytes, myoblasts and epithelial cells. [^12^](#3rdcrjn)^,^ [^18^](#2jxsxqh)  In the liver, stem cells are housed in the Hering's ducts/bile duct, these cells can give rise to precursor cells (oval cells) that are bipotential and can differentiate into biliary cells or hepatocytes. [^12^](#3rdcrjn)  In the brain reside neural stem cells that give rise to neurons, astrocytes, and oligodendrocytes. They have been described in two locations in adult brains, the subventricular zone and the dentate gyrus of the hippocampus. [^12^](#3rdcrjn)^,^ [^19^](#z337ya)  In the skin, stem cells are found in three regions of the epidermis, in the protrusion of the hair follicle, in the interfollicular regions (basal cells), and in the sebaceous glands. These cells give rise to amplification cells in transit and these in turn generate the cells of the differentiated epidermis. [^12^](#3rdcrjn)^,^ [^20^](#3j2qqm3)  In the intestinal epithelium, stem cells are found above Paneth cells in the small intestine and at the base of Lieberkühn's crypt in the colon. The cells give rise to the intestinal epithelium. [^12^](#3rdcrjn)^,^ [^21^](#1y810tw)  In cardiac and skeletal muscle, myocyte cell regeneration occurs by the replication of satellite cells, located mainly on the periphery of the myocyte sarcolemma. [^12^](#3rdcrjn)  In the cornea, the trunks of the limbus are located in the sclerocorneal limbus or sclerocorneal junction. These cells maintain the cells of the anterior epithelium of the cornea and with this help to maintain the transparency of the cornea, essential for optimal vision. [^12^](#3rdcrjn)^,^ [^22^](#4i7ojhp)  In 2001 Zuk y cols.[^23^](#2xcytpi) isolated and characterized mesenchymal stem cells (MSCs) from subcutaneous adipose tissue with the same differentiation potential found for BMSC. This discovery has opened up the possibility of obtaining stem cells without high-risk procedures, in addition to the fact that adipose tissue contains a large number of these cells compared to other adult tissues (5000-8000 ASC /ml). [^13^](#26in1rg)^,^ [^24^](#1ci93xb)^,^ [^25^](#3whwml4) If adipose tissue were divided into 2 components, these would be: the fatty component (adipocytes) and the stroma (loose connective tissue + vascular stromal fraction of adipose tissue or ADSVF). It is in this second that the ASC s are found. [^26^](#2bn6wsx)  69 type I diabetes mellitus, 70, 71 72 complex perianal fistula, 73, 74, tracheo-mediastinal fistula 75, rheumatoid arthritis 76, multiple sclerosis 77, maxilla reconstruction 78, zygomatic reconstruction 79, skull reconstruction 80, femoral head reconstruction 81 27  To date, a wide variety of specialties have used stem cell therapies derived from adipose tissue [^27^](#qsh70q) ([Table 2](#48pi1tg)). They have been used for the treatment of graft-versus-host disease [^65^](#2r0uhxc)^,^[^66^](#1664s55), idiopathic thrombocytopenic purpura [^67^](#3q5sasy)^,^ [^68^](#25b2l0r), pure red cell aplasia [^69^](#kgcv8k), type I diabetes mellitus [^70^](#34g0dwd)^,^ [^71^](#1jlao46), enterocutaneous fistula [^72^](#43ky6rz), complex perianal fistula [^73^](#2iq8gzs)^,^ [^74^](#xvir7l), tracheo-mediastinal fistula [^75^](#3hv69ve), rheumatoid arthritis [^76^](#1x0gk37), multiple sclerosis [^77^](#4h042r0), maxilla reconstruction [^78^](#2w5ecyt), zygomatic reconstruction [^79^](#1baon6m), skull reconstruction [^80^](#3vac5uf), femoral head reconstruction [^81^](#2afmg28) and post-prostatectomy stress urinary incontinence correction 82. The authors have reported decrease in healing time and increase in tissue regeneration. So far, no adverse effects have been reported from infusions or intralesional administration of ASC. It refers to a beneficial immunomodulatory effect. [^27^](#qsh70q)  In 2011, Ra et al. [^29^](#1pxezwc) published a study of 8 patients suffering from spinal cord injury who were treated with intravenous infusions of ASC. The author noted no serious adverse effects. At 12 weeks motor function improved in 4 patients, the author concluded that they could not determine the efficacy of ASC therapy because of the small group of patients and the short follow-up period. [^27^](#qsh70q)  **Uses of ASC in plastic surgery**  ASC s have been used in three main fields: soft tissue augmentation, wound healing, and tissue engineering [^27^](#qsh70q) ([Table 3](#2nusc19)).  **Soft tissue augmentation**  Several studies have been published using the ADSVF-enriched autologous adipose tissue (AT) transplantation technique for the treatment of facial atrophy[^30^](#49x2ik5)^,^ [^31^](#2p2csry)^,^ [^32^](#147n2zr), cosmetic breast augmentation and immediate breast augmentation after surgery. removal of an implant [^31^](#2p2csry)^,^ [^33^](#3o7alnk)^,^ [^34^](#23ckvvd), *pectus excavatum* [^31^](#2p2csry), loss of soft tissue due to trauma or iatrogenesis or degenerative diseases [^31^](#2p2csry), treatment for depressed scars [^27^](#qsh70q)^,^ [^35^](#ihv636) And for the treatment of localized scleroderma (in *saber stroke*) [^9^](#4d34og8). These authors report in all their cases improvement in soft tissue volume increase, greater graft survival, improvement in skin characteristics, and they concluded that the soft tissue augmentation technique is safe and effective. However, the studies were not compared with control groups.  **Wound healing**  ASC s and their potential for wound healing have been shown in several in vitro or in vivo studies. ASC and its secretory factors have shown improvement in chronic wound healing in animal models and in clinical trials. [^36^](#32hioqz) The first clinical application of ASC was a case report where a “calvarium” defect was treated after injury. After three months of treatment, new bone formation was reported and there was almost complete wound healing.  Subsequently, Rigoti and Akita have used them for the treatment of post-radiotherapy ulcers with favorable results.[^27^](#qsh70q)  So far, the mechanism of action has not been adequately described. It is proposed that the use of ASC has immunomodulatory effects that prove favorable conditions for tissue growth. This is speculated as ASC s have the ability to secrete growth factors, cytokines, and chemoattractants that enhance angiogenesis and increase blood supply to tissues thus creating or providing support to cells. To date, no cases of neoplasms have been reported in patients treated with lipoinjection or direct ASC injection. Recent clinical studies using ASC in other pathologies indicated no risk for tumor formation with the use of ASC. However, long-term follow-up studies are needed to establish the adverse effects of using these cells. [^36^](#32hioqz)  Tissue engineering  The general principles of tissue engineering strategies incorporate a combination of three factors: 1.- Living cells that are embedded in the defect site, 2.- A three-dimensional (3D) protective structure (scaffold) of cells in characteristic structural, functional and mechanical conditions, 3.- Creation of a microenvironment to provide additional factors that ultimately promote the growth and formation of new tissue. When new tissue is formed, the biodegradable scaffold structure is replaced. [^37^](#1hmsyys)  Many biomaterials have been investigated with the aim of being used as scaffolds in the construction of adipose tissue engineering, both natural and synthetic polymers. [^37^](#1hmsyys)  The advancement of tissue engineering as a regenerative therapy relies on rapid vascularization of tissue constructs formed ex vivo by the host's vasculature. Mesenchymal stem cells may be beneficial in promoting rapid assembly and growth of vasculature to support tissue engineering results in vivo. [^38^](#41mghml)  Under these conditions they have been used for the regeneration of bone tissue [^39^](#2grqrue)^,^, cartilage [^40^](#vx1227), muscle [^37^](#1hmsyys) And nerve. [^41^](#3fwokq0)  **Experimental studies carried out with ASC.**  Experimental studies with ASC have been carried out mainly in rodents [^42^](#1v1yuxt)^,^ [^43^](#4f1mdlm)^,^ [^44^](#2u6wntf)^,^ [^45^](#19c6y18)^,^ [^46^](#3tbugp1)^,^ [^47^](#28h4qwu)^,^ [^48^](#nmf14n)^,^ [^49^](#37m2jsg)^,^ [^50^](#1mrcu09). Some studies in rabbits [^51^](#46r0co2) and pigs have also been reported [^52^](#2lwamvv). They have basically focused on wound healing in diabetic rodents [^42^](#1v1yuxt), [^43^](#4f1mdlm)^,^ [^44^](#2u6wntf)^,^ [^45^](#19c6y18)^,^ [^46^](#3tbugp1)^,^ [^47^](#28h4qwu) And in irradiated rodents [^49^](#37m2jsg)^,^[^50^](#1mrcu09). They have been applied in conjunction with small intestinal submucosal scaffolds, acellular dermal matrix, and in scaffolds composed of collagen, chondroitin sulfate, and hyaluronic acid. No significant differences were found between them. Healing was better in the ASC and scaffold group than in the control groups. The use of ASC with scaffolds was not compared with ASC without scaffolds ^16^. Results with the application of ASC in isolation in excisional wounds in diabetic mice have reported acceleration of healing which occurred between days 9-18 compared with 28 days of diabetic rodents without application of ASC [^42^](#1v1yuxt)^,^ [^44^](#2u6wntf)^,^ [^45^](#19c6y18)^,^ [^46^](#3tbugp1)^,^ [^47^](#28h4qwu). The use of ASC on wounds in diabetic mice was found to equal healing time compared to the same wounds in healthy mice without ASC application [^48^](#nmf14n). Accelerated healing was due to the ability to synthesize and secrete growth factors in a hypoxic environment, released by ASC. The study by Zhu et al. [^53^](#111kx3o) supports these results by showing that ASC s promote increased gene expression and secretion of large amounts of angiogenic growth factors, including vascular endothelial growth factor (VEGF). Additionally, the secretion of TGF-β, KGF, FGF2, PDGF, HGF, fibronectin 1 and collagen 1 has been reported [^36^](#32hioqz). Lin et al. [^54^](#3l18frh) compared multilayered ASC sheets against single-layered ASC sheets. They reported improved wound healing in the murine excisional wound model using multilayered ASC sheets [^16^](#1ksv4uv).  The study by Cianfarani et al. [^55^](#206ipza) suggests that comorbidities such as diabetes could affect the potential of ASC in murine models, although the clinical implications of this assertion have not been established. Nambu et al. [^42^](#1v1yuxt) have been the only ones that have used diabetic mouse models and they still found improvement in wound healing. This suggests that comorbidities such as diabetes might not have an effect on the potential of ASC [^16^](#1ksv4uv).  El-Ftesi et al. [^57^](#4k668n3) showed that ASC from aged diabetic mice had an altered response to hypoxia (decreased ADSVF expression) when compared to ASC from healthy mice.  **Complications of the use of ASC**  In most of the published studies in which ASC has been used, no complications or adverse effects have been reported, although the clinical use of ASC is new. [^26^](#2bn6wsx)  In some clinical trials where ASC -enriched lipo-grafts were used, the development of subcutaneous bleeding as well as inflammation of 4 weeks duration was observed in six patients with facial lipoatrophy (one patient diagnosed with Parry-Roomberg syndrome and the remaining five diagnosed with lupus deep erythematous). They were divided into two groups: the control consisted of three patients who were injected with subcutaneous fat tissue in the area with lipoatrophy, while the experimental group consisting of three patients was injected with ASC -enriched fat tissue. Therapeutic maneuvers were not performed to treat the subcutaneous bleeding that some patients presented. This complication resolved in all patients after 1 to 2 weeks [^30^](#49x2ik5). In clinical trials, the presence of ectopic fibrogenesis and distal lymphadenopathy was observed in two patients [^57^](#2zbgiuw). Unfavorable behavior of mesenchymal stem cells, such as differentiation into myofibroblasts, has also been reported [^58^](#1egqt2p). For this reason, it is suggested that ASC s should be attached to cells, tissues, or biological scaffolds before being administered, to avoid unexpected migration or differentiation [^59^](#3ygebqi).  Some publications conclude that ASC can increase the growth of active tumor cells, but not those that are at rest (G0) [^26^](#2bn6wsx)^,^ [^60^](#2dlolyb).  In a study in which immunodeficient mice were injected with human ASC in subcutaneous tissue, no teratoma formation was found [^15^](#35nkun2).  **Current status, application and regulation uncertainty.**  The Food and Drug Administration (FDA) has developed a regulatory framework based on three areas: prevention of the use of contaminated tissues and cells; prevention of improper handling and processing that could damage or contaminate cells or tissues; and the clinical safety of all tissues and cells that may be processed, used for purposes other than their normal functions, combined with components other than tissues, or used for metabolic purposes. In the United States of America, ASC are considered in the context of human cells, human tissues, so their products and their production must comply with the current requirements of good tissue practice, regulated under the Code of Federal Regulations, Title 21, Part 1271. [^27^](#qsh70q)  In Europe, ASC s are considered advanced therapy medicinal products, defined in this way by the European Regulation (European Commission), which contains rules for "authorization", supervision and technical requirements regarding the summary of product characteristics, the labeling, and packaging of advanced therapy medicinal products that are prepared by industry and by academic institutions. [^27^](#qsh70q)  The process of converting from research-based protocols using ASC to a safe manufacturing process that has good manufacturing process requires protocols that have carefully considered all risks and benefits to the patient. Sensebe et al. established the following parameters that should be considered: collection sources and methods, cell sowing, proliferation rate, and culture medium. [^27^](#qsh70q)  In Mexico, the General Health Law published its last reform in the Official Gazette of the Federation on 01/15/2014. This stipulates in its Title XIV (donation, transplants and loss of life) in article 330 the following:  Article 330: The National Transplant Center will be in charge of the National Transplant Registry, which will integrate and keep the following information updated:  I. The registry of authorized establishments in accordance with article 315 of this Law.  II. The transplant surgeons responsible for the extraction and transplants, and the hospital donation coordinators.  III. Data on transplants with the exception of autotransplants and those relating to progenitor or stem cells.  The registry of progenitor or stem cell transplants will be in charge of the National Center for Blood Transfusion. [^61^](#sqyw64)  Regarding the use of ADSVF in localized and systemic scleroderma, there are few studies, most are isolated case reports, which have shown improvement in skin lesions. For example, there are reports of the application of fat grafts for localized scleroderma (in saber stroke), Consorti et al. reported the case of a 34-year-old woman affected with scleroderma located in the frontal-orbital region, asymmetry in the arch of the eyebrow, both on the right side of the face. Fat grafts were performed. After two years of follow-up, the patient's condition was satisfactory, with improvement in the symmetry and morphology of the fronto-orbital region, as well as in cell atrophy and texture [^6^](#tyjcwt). Oh et al. reported a report of a 21-year-old man with a trilinear atrophic depression on the forehead and frontal portion of the scalp, secondary to localized scleroderma. A 50% fat graft and a 50% dermis graft were injected in the regions with depression. The patient presented convex elevation of the shape in the postoperative period until approximately two months when the injected areas already presented characteristics similar to the surrounding areas and the dark brown appearance of the atrophic lines had disappeared [^7^](#3dy6vkm). It is concluded that the use of fat grafts with ADSVF has good results, and no complications are reported [^5^](#2et92p0). Bank et al. carried out a series of 13 cases (12 women and one man) of patients. Nine had scleroderma, two had mixed connective tissue disease, and two had primary Raynaud's phenomenon. Each hand with manifestations was taken separately for the application of hand grease. A total of 21 hands were treated. In the pretreatment, pain and functional level were evaluated. At the end of the study, an improvement in pain was found after the application of fatty tissue (p<0.001). 18 of the 21 hands had a decrease in pain, the remaining 3 did not present changes in pain. 20 of the 21 hands presented improvement in cold attacks (p<0.001) as well as improvement in the severity of cold attacks, since the 21 hands had a decrease in it (p<0.001). 12 of the 21 hands had ulcers before treatment; 5 of the 21 hands continued to have ulcers after treatment. As a conclusion Bank et al. describe that this procedure appears to be safe and simple for the treatment of this condition. The improvement of the patients is attributed to angiogenesis and a decrease in the pathways that promote healing and fibrosis, this attributed to ADSVF [^8^](#1t3h5sf). It has also been used experimentally for the treatment of skin lesions caused by systemic sclerosis, mice were injected subcutaneously with bleomycin to cause skin sclerosis. Once the sclerosis was provoked, the rats were injected with ADSVF at the site of the sclerosis, showing a decrease in the fibrosis of the dermis and a proangiogenic effect [^5^](#2et92p0).  This has attracted attention for the application of ADSVF in the treatment of digital ulcers caused by systemic sclerosis. Only this author has used ADSVF for the treatment of localized systemic sclerosis (en coup de saber) with good results [^9^](#4d34og8)  There is currently a protocol (SCLERADEC [NCT01813279]. www.clinicaltrials.gov) carried out in Marseille, which aims to evaluate the effects of injection of autologous vascular stromal fraction of adipose tissue (ADSVF) in patients with systemic sclerosis who present functional alterations in the hands, performing a subcutaneous injection of ADSVF in the fingers in contact with neurovascular pedicles in 11 patients with scleroderma with a one-year follow-up. Preliminary results from Dr. Del Papa's group showed a decrease in ulcer healing time and an improvement in the number of capillaries in 15 patients with ES who were administered stem cells derived from autologous adipose tissue (Del Papa N, Di Luca G, Sambataro D, et al. regional implantation of adipose tissue-derived cells induces a prompt healing of long-lasting indolent digital ulcers in patients with systemic sclerosis. Arthritis & Rheumatology 2014; supplement 1, S737).  In addition, there are data to suggest that the administration of autologous hematopoietic stem cells (which are found in large numbers in ADSVF) may produce clinical improvement in various clinical, vascular, and immunological parameters in patients with systemic sclerosis. An example of this are the results of the ASTIS study that recruited patients with severe, early SSc and showed that, although there was a higher early mortality associated with transplantation (due to severe infections), said treatment was associated with greater long-term disease-free survival., decrease in skin fibrosis and stabilization of damage to internal organs.[^83^](#39kk8xu)  There is also indirect evidence from case reports. For example: Guiducci et al, report the case of a patient diagnosed with systemic sclerosis, who developed acute gangrene in the extremities. Treatment consisted of 3 intravenous infusions of autologous mesenchymal stem cells obtained from bone marrow. The results of the angiography and the high expression of angiogenic factors in tissue regeneration obtained one week after the administration of the third pulse, suggest that the mesenchymal stem cells promoted the formation of new vessels and vascular remodeling in the extremities. [^10^](#2s8eyo1) |
| **9. Definition of the problem** |
| The current conventional treatment of digital ulcers consists of vasodilators, antiplatelet agents, analgesics, control of systemic sclerosis, concomitant diseases, and continuous treatment to avoid local infections until secondary healing is obtained. During this period, patients need multiple consultations and treatment, they have pain and significant disability, which can be irreversible, and they are in risk of infections and tissue loss. |
| **10.Justification** |
| Due to pain, the long time needed to obtain secondary healing, the disability they produce, and the high cost that the treatment of digital ulcers generates for the patient, the local application of vascular stromal fraction derived from adipose tissue is proposed, since there is experimental and clinical evidence to suggest that it accelerates the healing process of chronic wounds associated with vasculopathy. As there are no previous controlled clinical trials that evaluate the use of ADSVF in this pathology, we propose a pilot study to evaluate safety and obtain data that allows calculating a sample size to subsequently design a study to evaluate the efficacy of the use of ADSVF. |
| **11. Hypothesis** |
| The use of lipo-graft enriched with the vascular fraction of adipose tissue injected subcutaneously in patients with digital ulcers, will be safe in patients with digital ulcers secondary to SSc.  As a secondary hypothesis: The use of lipo-graft enriched with the vascular fraction of adipose tissue injected subcutaneously in patients with digital ulcers will allow to accelerate the secondary healing process compared to conventional medical and surgical treatment. |
| **12. Objectives** |
| **Main objective**  Evaluate the safety of the administration of vascular stromal fraction of adipose tissue (autologous) in patients with ischemic digital ulcers associated with systemic sclerosis.  Secondary Objectives.   1. Evaluate the effect of the proposed treatment on the healing time of the index ulcer. 2. Evaluate the improvement in pain in the hand. 3. Evaluate the frequency, intensity and duration of Raynaud Phenomenon. 4. Evaluate improvement in hand function. 5. Evaluate the improvement in the quality of life. 6. Evaluate the effect of the proposed treatment on the number of nail capillaries by videocapillaroscopy. 7. Evaluate the immunomodulatory effect of the administration of ADSVF in patients with digital ulcers. 8. Evaluate the reproducibility of the extraction procedure of the vascular fraction of adipose tissue (ADSVF) 9. As an exploratory objective, we wish to determine if the administration of ADSVF in patients with digital ulcers decreases the concentration of the following inflammatory markers: erythrocyte sedimentation rate (ESR), C-reactive protein (CRP), endothelin-1, (ET-1), molecule of intercellular adhesion (ICAM-1), interleukin 1 (IL-1), interleukin 6 (IL-6), interleukin 17 (IL-17), interleukin 22 (IL-22), vascular endothelial growth factor (VEGF) |
| **13. Methodology: General design** |
| Prospective, longitudinal, randomized, controlled, not blinded.  In the initial assessment, x-rays of the hands in AP and oblique projection will be requested, as well as a pregnancy test will be carried out for patients of childbearing age to confirm that they are not pregnant, since pregnancy is the reason for exclusion.  **OBTAINING AND ISOLATION OF ADSVF:**  In the initial assessment, x-rays of the hands in AP and oblique projection will be requested, as well as a pregnancy test will be carried out for patients of childbearing age to confirm that they are not pregnant, since pregnancy is the reason for exclusion.  The procedure will be performed in the operating rooms of the outpatient unit (UPA) of the INCMNSZ. Preoperatively, percutaneous oximetry of all the fingers of the hand will be performed, with a portable oximeter, to determine the degree of perfusion as a control.  With the patient standing, the fat deposits susceptible to liposuction for fat extraction will be marked.  As prophylaxis an IV dose of Amoxicillin and Sulbactam 1g/500mgr IV will be applied or in case of being allergic Ciprofloxacin at a dose of 400mg IV DU. Additionally, Ketorolac 30 mg IV DU will be applied. Local anesthesia with Klein's Solution (1000 ml Hartmann Solution, 25 cc of 2% simple Xylocaine, 1 ml of adrenaline and 10 mEq of bicarbonate) and intravenous sedation will be administered.  100 to 200 ml of Klein's solution will be infiltrated (tumescent technique) in the site with the highest concentration of subcutaneous adipose tissue susceptible to liposuction. Fifteen minutes later, liposuction of the area will be performed, with a blunt cannula with 2 mm diameter holes and a 20 ml syringe. A minimum of 100 ml of fat will be extracted. Of this amount, 60 ml will be placed in sterile vials with Hank's Balanced Salt Solution (HBSS) with 5% albumin and will be immediately transported to the INCMNSZ Department of Nutritional Physiology for processing.  ADSVF will be isolated from adipose tissue by the collagenase separation method described by Rodbell as follows:  The tissue will be washed with sterile phosphate-buffered saline with antibiotic-antifungal (1%) at 37°C twice to remove residual blood, then incubated for 40 min in a 5% collagenase solution in HBSS. Once disintegrated, the cells will be filtered through a 100-micron mesh and centrifuged at 1000 rpm for 5 min to separate the vascular stromal cells from the adipocytes. The supernatant will be removed, and the compacted cells are resuspended in 30 ml of sterile HBSS at 37°C, the density and viability are quantified.  Forty ml of aspirated fat will be placed in a 50 ml syringe and kept at rest to decant it. The blood and serum that separate from the fat will be eliminated.  The procedure will be carried out under the provisions of the General Law Title XIV (donation, transplants and loss of life) article 330.  **ADSVF APPLICATION:**  The previously extracted and characterized vascular stroma will be mixed with the 40 ml. of fat that were kept in the 50 ml syringe. Local anesthesia with 2% Xylocaine without epinephrine will be applied to the median, ulnar and radial nerve at the wrist level, in order to obtain anesthesia of the entire hand. After mixing, this solution will be placed in 1 ml and 3 ml syringes. With a 19-gauge (0.8 mm) needle, 0.5 ml will be applied to the radial and ulnar border of each phalanx of each finger (3 ml per finger) and 10 ml subcutaneously distributed throughout the back of the hand, and 5 ml subcutaneously distributed in the palmar region.  Digital finger oximetry will be performed one now after finishing the fat infiltration. Likewise, pain will be evaluated, and IV analgesics will be applied if necessary.  An occlusive dressing will be placed on digital ulcers.   1. Sample size. This is an exploratory study to evaluate the reproducibility of the ADSVF. extraction procedure and the safety of the fat graft in patients with ischemic digital ulcers associated with systemic sclerosis. The results obtained in this study will serve as a basis for calculating the sample size for a subsequent study where the efficacy of fat grafting in these patients will be evaluated. 2. Mechanism of treatment assignment: random 3. Treatment groups: a) Control group with conventional treatment. b) Study group with conventional treatment plus application of the stromal vascular fraction of adipose tissue. 4. Duration of individual follow-up: 6 months from the zero visit. |
| **14. Temporality of the study** |
| \| **Study type** \| **Select an option** \| \| --- \| --- \| \| Retrospective \|  \| \| Prospective \| X \| |
| **15. Assignment process of the study group** |
| \| Maneuver \| Yes (Include the corresponding information) \| No \| Does not apply \| \| --- \| --- \| --- \| --- \| \| Randomization \| X \|  \|  \| \| Open study \| X \|  \|  \| \| Single-blind study \|  \| X \|  \| \| Double-blind study \|  \| X \|  \| \| Triple-blind study \|  \| X \|  \| |
| **16.Description of the maneuvers or interventions** |
| Once the patient has completed the informed consent procedure, randomization will be carried out to determine the group in which the patient will participate and the visit will be scheduled on the initial day, which will be called day zero.  **Zero-day visit.**  **Pre-treatment phase:**  All participants in the clinical trial will undergo an initial evaluation consisting of:   1. Evaluation of ulcers secondary to systemic sclerosis (the quantity, location, and size of the ulcers will be evaluated). 2. Photographs of the hands with ulcers (of the palmar and dorsal region) will be taken. 3. A videocapillaroscopy will be performed, a study that will help us to know changes in the digital microcirculation. 4. A transcutaneous oximetry with a portable oximeter will be performed on each finger, this in order to know the degree of perfusion. 5. Pain assessment (Visual Analogue Scale) will be performed. 6. Carry out a special questionnaire for patients with scleroderma ulcers. Functionality evaluations will be carried out with the questionnaires (SHAQ and COCHIN). 7. A questionnaire will be carried out to find out their quality of life (SF 36). 8. AP and oblique hand radiographs will be taken. 9. Standard evaluation of systemic sclerosis will be carried out, which includes determination of the modified Rodnan score and the parameters of the Medsger severity scale. This follow-up is carried out periodically on all the participants of the Institute's systemic sclerosis cohort. 10. An immunological pregnancy test will be performed on all female participants of childbearing age. 11. Standard treatment for ischemic digital ulcers will be prescribed that includes the allowed medications as described in the inclusion and exclusion criteria of the protocol. 12. At the visit on day 0 and on day 84, a peripheral venous blood sample will be taken. Peripheral blood mononuclear cells (PBMC) will be isolated by gradient centrifugation with Ficoll-Paque (Amersham Bioscience, Diegem, Belgium) and serum will be obtained from a 2020 ml sample of peripheral blood.   Subpopulations of T cells (Th1, Th2, Th17, Treg) and regulatory B cells will be detected using previously published methodology, which is summarized below: [^62^](#3cqmetx)  1 x 10^6^ peripheral blood mononuclear cells (PBMCs) will be stained with 5 μL of monoclonal antibodies anti-CD4 stained with PECy5 and anti-CD14 stained with FITC (BD Biosciences, San José CA), at room temperature, in the dark, for 20 minutes After 2 washes, the PBMCs will be permeabilized with 200 μL of cytofix/cytoperm solution (BD Biosciences) at 4°C for 20 min; after 2 washes with permwash solution (BD, Biosciences) PBMCs will be stained for intracellular cytokines and transcription factors with PE-stained anti-IFNγ for Th1 cells (BD, Biosciences), PE-stained anti-IL-4 for cells Th2 (BD, Biosciences), PE-stained anti-IL-17 for Th17 cells (clone eBio64CAP17, mouse IgG11K, from eBioscience, San Diego, CA) and PE-stained anti-Foxp3 for regulatory T cells (eBioscience; clone 259D /C7, mouse IgG1K) for 30 min at 4°C in the dark. Finally, after washing with permwash solution, PBMCs will be analyzed for subtypes with flow cytometry using a FACScan (BD Biosciences). A window for CD4+/CD14- cells will be used, 50,000 events will be recorded for each sample and analyzed with the CellQuest program (BD, Biosciences). Results will be expressed as the percentage of cells expressing IFNγ, IL-4, IL-17 or Foxp3 in each window. Isotype controls (murine IgG1-FITC/IgG1-PE/CD45-PeCy5/IgG1k (BD Tritest, BD Biosciences)) will be used to establish thresholds and windows on the cytometer. To avoid false positive PE results and to establish compensation for multicolor cytometric analysis, instrument calibration procedures will be performed every day according to the protocols established by our laboratory. Briefly, a smear without dye (autofluorescence control) and a smear with a sample of permeabilized PBMCs will be performed. autofluorescence (unstained cells) will be compared to single stain positive controls to confirm that stained cells were on scale for each parameter. In addition, 3 BD calibration beads will be used to adjust instrument parameters, establish fluorescence offsets, and revise instrument sensitivity (BD CaliBRITETM, BD, Biosciences).  Erythrocyte sedimentation rate (ESR), C-reactive protein (CRP), endothelin-1 (ET-1), intercellular adhesion molecule (ICAM-1), interleukin 1 (IL-1), interleukin 6 (IL- 6), interleukin 17 (IL-17), interleukin 22 (IL-22), vascular endothelial growth factor (VEGF). The ESR will be determined the day the sample is taken by Westergern, the ultrasensitive PCR will be determined the day the sample is taken by nephelometry. The rest of the sample will be frozen for subsequent processing by luminometry following the manufacturer's instructions (Bioplex, Biorad, USA).  PBMCs will be labeled with 5 IL of anti-CD4-marker-PECy5 and anti-CD14-marker-FITC monoclonal antibodies at room temperature in the dark for 20 min. After two washes, PBMCs will be permeabilized with 200 IL of cytofix/cytoperm solution at 4 °C for 20 minutes. After two washes with permwash solution, PBMCs will be labeled for intracellular cytocones and transcription factors with PE-labeled-anti-IL4 for the Th2 subpopulation, PE-labeled-anti-INF-c for the Th1 subpopulation, PE-labeled-anti -IL17 for the Th17 subpopulation and PE-labeled-anti-Foxp3 for the regulatory T cell subpopulation for 30 minutes at 4°C in the dark. Finally, after washing with permwash solution, the subpopulations of PBMCs will be analyzed with flow cytometry with a FACScan. An electronic "gate" will be made for CD4/CD14 cells and a total of 50,000 events will be recorded for each sample and analyzed with the CellQuest software. Results will be expressed as the relative percentage of INF-c, IL-4, IL-17, or Foxp3 that is expressed in each cell at each gate. Isotype controls will be taken, IgG1-FITC/IgG1-PE/CD45-PeCy5 mouse IgG1, k will be used to establish the threshold and gates of the cytometer. In order to avoid false positive PE results and also for compensation adjustment multicolor flow cytometry analysis will be done. The instruments will be calibrated every day according to the protocols established in the laboratory.  In patients assigned to treatment with ADSVF, on day zero, in addition to the evaluations described above, the following procedures will be performed:  **OBTAINING AND ISOLATION OF ADSVF:**  The procedure will be performed in the operating rooms of the outpatient unit (UPA) of the INCMNSZ. Preoperatively, percutaneous oximetry of all the fingers of the hand will be performed, with a portable oximeter, to determine the degree of perfusion as a control.  With the patient standing, the fat deposits susceptible to liposuction for fat extraction will be marked.  As prophylaxis an IV dose of Amoxicillin and Sulbactam 1000/500 mg IV will be applied or in case of being allergic Ciprofloxacin at a dose of 400 mg IV DU. Additionally, Ketorolac 30 mg IV DU will be applied. Local anesthesia will be administered with Klein's Solution (1000 ml Hartmann Solution, 25 cc of 2% simple Xylocaine, 1 ml of adrenaline and 10 mEq of bicarbonate) and intravenous sedation.  100 to 200 ml of Klein's solution will be infiltrated (tumescent technique) in the site with the highest concentration of subcutaneous adipose tissue susceptible to liposuction. Fifteen minutes later, liposuction of the area will be performed, with a blunt cannula with 2 mm diameter holes and a 20 ml syringe. A minimum of 100 ml of fat will be extracted. Of this amount, 60 ml will be placed in sterile vials with Hank's Balanced Salt Solution (HBSS) with 5% albumin and will be immediately transported to the INCMNSZ Department of Nutritional Physiology for processing.  ADSVF will be isolated from adipose tissue by the collagenase separation method described by Rodbell as follows:  The tissue will be washed with sterile phosphate-buffered saline with antibiotic-antifungal (1%) at 37°C twice to remove residual blood, then incubated for 40 min in a 5% collagenase solution in HBSS. Once disintegrated, the cells will be filtered through a 100-micron mesh and centrifuged at 1000 rpm for 5 min to separate the vascular stromal cells from the adipocytes. The supernatant will be removed, and the compacted cells are resuspended in 30 ml of sterile HBSS at 37°C, the density and viability are quantified.  Forty ml of aspirated fat will be placed in a 50 ml syringe and kept at rest to decant it. The blood and serum that separate from the fat will be eliminated.  **APPLICATION OF FATGRAFT ENRICHED WITH ADSVF:**  The previously extracted and characterized vascular stroma will be mixed with the 40 ml. of fat that were kept in the 50 ml syringe. Local anesthesia with 2% Xylocaine without epinephrine will be applied to the median, ulnar and radial nerve at the wrist level, in order to obtain anesthesia of the entire hand. After mixing, this solution will be placed in 1 ml and 3 ml syringes. With a 19-gauge (0.8 mm) needle, 0.5 ml will be applied to the radial and ulnar border of each phalanx of each finger (3 ml per finger) and 10 ml subcutaneously distributed throughout the back of the hand, and 5 ml subcutaneously distributed in the palmar region.  Digital finger oximetry will be performed one now after finishing the fat infiltration. Likewise, pain will be evaluated, and IV analgesics will be applied if necessary.  An occlusive dressing will be placed on digital ulcers.  **Phase of follow-up and evaluation of results**  Regardless of the group to which it is assigned, follow-up will be carried out first weekly and then every 28 days (every month) until day 168 (up to 6 months).  In the visits of days 7, 14, 21, 28, 56, 84, 112, 140 and 168 all patients will be performed:   1. Evaluation of ulcers secondary to systemic sclerosis (the amount, location, size of the ulcers will be evaluated) which will be carried out weekly during the first month and subsequently monthly (days 28, 56, 84, 112, 140, 168) up to 6 months (day 168). 2. Hand x-rays will be performed in 2 positions (at visit zero and at 168 days, 6 months). 3. Photographs of the hands with ulcers (of the palmar and dorsal region) will be taken, which will be carried out weekly during the first month and subsequently monthly (days 28, 56, 84, 112, 140, 168) until 6 months (day 168). 4. Videocapillaroscopy will be performed at the baseline visit, on day 28 and on day 168 (6 months). 5. Transcutaneous oximetry will be carried out with a portable oximeter on each finger, this in order to evaluate the perfusion immediately after surgery, weekly during the first month and then monthly until 6 months. 6. Pain assessment will be carried out weekly during the first month and subsequently monthly, then monthly until 6 months (days 28, 56, 84, 112, 140, 168). 7. The SHAQ and COCHIN questionnaires, the questionnaire for patients with scleroderma ulcers (SHAQ, COCHIN) will be administered one week after surgery, one month (day 28) and 6 months (day 168). 8. A questionnaire will be carried out to find out quality of life (SF 36) a week after surgery, a month (day 28) and 6 months (day 168). |
| **17. Treatments (if applicable) (include a table for each study drug)** |
| \| Medication 1 \| Include the relevant information \| Not \| Does not apply \| \| --- \| --- \| --- \| --- \| \| Name \|  \|  \| X \| \| Does it fulfill "Good Manufacturing Practices"? \|  \|  \| X \| \| Codes, labeling, storage, retention and protection of medication samples \|  \|  \| X \| \| Pharmaceutical dosage form \|  \|  \| X \| \| Dosage \|  \|  \| X \| \| Administration interval \|  \|  \| X \| \| Route of administration \|  \|  \| X \| \| Rate of administration \|  \|  \| X \| \| Treatment duration \|  \|  \| X \| |
|  |
| **18. Tracking** |
| \|  \| Include the relevant information \| Not \| Does not apply \| \| --- \| --- \| --- \| --- \| \| Number of study phases \|  \|  \| X \| \| Number of visits and their schedule (include times) \| 10 visits in the morning \|  \|  \| \| Duration of each phase of the study \|  \|  \| X \| \| Labs tests and diagnostic procedures that will be used \| AP and oblique X-rays of both hands  Videocapillaroscopy \|  \|  \| \| Follow-up duration \| 168 days \|  \|  \| \| Sampling methods \|  \|  \| X \| \| Treatment options to be offered at the end of the study \|  \|  \| X \| |
|  |
| **19. Management of Drug Overdose** |
| Does not apply |
| **20. First Aid and Rescue therapy** |
| Wound healing, analgesics, antibiotic therapy, antiplatelet drugs and hyperbaric chamber therapy |
| **21. Permitted concomitant therapies** |
| Usual treatment as directed by your treating physician, except those described in section 22 |
| **22. Prohibited concomitant therapies** |
| 1. Patients with vasodilator drug treatment (such as calcium channel antagonists, Angiotensin-converting enzyme inhibitors, nitroglycerin, alpha-adrenergic blockers, angiotensin II receptor antagonists, 5-phosphodiesterase inhibitors, endothelin receptor inhibitors, prostanoids), N-acetylcysteine, antiplatelet therapy, conventional heparin or Low-molecular-weight heparin, who have received this treatment for less than 2 weeks prior to the selection visit or whose treatment has not been stable for this period. 2. Immunomodulatory drug treatment (such as prednisone or its equivalent, cyclophosphamide, azathioprine, mycophenolic acid, D-penicillamine, methotrexate, chloroquine, hydroxychloroquine, leflunomide or any other drug immunosuppressant) that has been started within 3 months prior to the selection visit or whose treatment has not been stable for at least 1 month prior to the selection visit. 3. Use of topical growth factors or hyperbaric oxygen concomitant to the study. 4. Local injection of botulinum toxin during the study or up to 4 weeks prior to the baseline visit. 5. Upper extremity surgical sympathectomy or surgical wound debridement within one month prior to baseline visit. 6. Concomitant treatment with another experimental drug within 4 weeks prior to the study or during the study. |
| **23. Definition of monitoring variables** |
| Presence of adverse events  Healing time of digital ulcers  Improvement in hand pain  Improvement in Raynaud's Phenomenon  Improvement in hand function  Improvement in quality of life  Number of capillaries per field in videocapillaroscopy  Immunomodulatory effect of treatment (proportions of subpopulations of CD4+ T cells)  Inflammation marker levels and cytokines |
| **24. Methods that will be used to collect information** |
| An Excel table will be used in which the data variables under study will be collected (attached as an appendix). |
| **25.Monitoring procedure and audits during the development of the study** |
| Does not apply. |
| **26. Failure and success criteria** |
| Success (primary outcome).  Total healing of the index digital ulcer within the observation period, with or without improvement in pain, hand function, and quality of life.  Improvement (secondary outcomes).   1. Improvement in hand pain. 2. Improvement in hand function. 3. Improvement in quality of life. 4. Increase in the number of capillaries per field in videocapillaroscopy.   Failure.   1. Lack of healing within the study period. 2. Increased pain according to the scale performed before treatment. 3. Impaired function of the hand. 4. Deterioration in quality of life.   Adverse events:   1. Infection of the ulcer or ADSVF injection site 2. Infection of the adipose tissue collection site 3. Tissue necrosis at the ADSVF application site 4. Impaired hand function 5. Deterioration in quality of life 6. Increased pain 7. Appearance of new ulcers 8. Any event that requires unscheduled hospitalization of the patient (related or not to treatment), prolongation of hospitalization, or death will be considered an adverse event. |
| **27. Sample size (please include the formula used for the calculation and the source of information on which the assumptions were based)** |
| This is a pilot study to evaluate the safety of ADSVF administration in digital ulcers in patients with systemic sclerosis. There are no previous studies that make it possible to adequately calculate the sample size, so the results of this study will provide information to be able to calculate the sample size for a study that will evaluate the efficacy of ADSVF on healing time of digital ulcers in Systemic Sclerosis patients. |
| **28. Description of the techniques, devices and/or instruments to be used in the measurement (Including: special mechanical, electronic, cybernetic equipment)** |
| Videocapillaroscopy. Optilia 200x video capillaroscope.  Omron pulse oximeter or equivalent. |
| **29.Description of the evaluation formats, questionnaires, comparison tables, etc., indicating the criteria of validity, reproducibility and quality controls used for them.** |
| COCHIN  SHAQ  SF-36  (Attached as appendix) |
| **30.Does the protocol involve the handling and labeling of biological samples? If applicable, mention the procedures that will be used** |
| The adipose tissue obtained from each patient will be labeled with the name and registration number of each patient, as well as the date it was obtained. Each patient will also be assigned a consecutive number that will be attached to the labels of the adipose tissue and all tubes. Throughout the processing of the tissue and the obtaining of the ADSVF, all the tubes will be marked with the initials, the registration number of each patient and the assigned protocol number, since it is an open study (unblinded) it is not necessary to code the samples to blind them.  Yes, with the patient standing, the fat deposits susceptible to performing liposuction for fat extraction will be marked.  As prophylaxis, an IV dose of Amoxicillin and Sulbactam 1g/500mg IV will be applied or in case of being allergic Ciprofloxacin at a dose of 400mg IV DU. Additionally, Ketorolac 30 mg IV DU will be applied. Local anesthesia will be administered with Klein's Solution (1000 ml Hartmann Solution, 25 cc of 2% simple Xylocaine, 1 ml of adrenaline and 10 mEq of bicarbonate) and intravenous sedation.  100 to 200 ml of Klein's solution will be infiltrated (tumescent technique) in the site with the highest concentration of subcutaneous adipose tissue susceptible to liposuction. Fifteen minutes later, liposuction of the area will be performed, with a blunt cannula with 2 mm diameter holes and a 20 ml syringe. A minimum of 100 ml of fat will be extracted. Of this amount, 60 ml will be placed in sterile vials with Hank's Balanced Salt Solution (HBSS) with 5% albumin and will be immediately transported to the INCMNSZ Department of Nutritional Physiology for processing.  ADSVF will be isolated from adipose tissue by the collagenase separation method described by Rodbell as follows:  The tissue will be washed with sterile phosphate-buffered saline with antibiotic-antifungal (1%) at 37°C twice to remove residual blood, then incubated for 40 min in a 5% collagenase solution in HBSS. Once disintegrated, the cells will be filtered through a 100-micron mesh and centrifuged at 1000 rpm for 5 min to separate the vascular stromal cells from the adipocytes. The supernatant will be removed, and the compacted cells are resuspended in 30 ml of sterile HBSS at 37°C, the density and viability are quantified.  Forty ml of aspirated fat will be placed in a 50 ml syringe and kept at rest to decant it. The blood and serum that separate from the fat will be eliminated. |
| **31. Corresponding information to ensure that the biological samples obtained will not be used for permanent or immortal cell lines or for purposes not related to the study** |
| The biological samples obtained will be used in their entirety for the purposes of this study. In the event that a part of these is not used, they will be discarded in accordance with the corresponding regulations. Participating researchers will ensure that remaining biological samples are discarded. |
| **32. Description of treatment groups** |
| Control group with conventional treatment.  stromal vascular fraction of adipose tissue. |
| **33. Mechanisms for treatment assignment** |
| Randomization. |
| **34. If a placebo group is used, include your justification** |
| Our study does not include a placebo group, it includes a group of patients who will not receive the intervention but who will continue with conventional treatment. Due to the nature of the intervention (ADSVF injection obtained from adipose tissue) it is not possible to blind the study, nor would it be feasible or ethical to offer a "white" intervention (sham) since it is not justified to submit to liposuction and injection of any solution without ADSVF in patients of the control group. |
| **35. Criteria for premature withdrawal from the study** |
| Only by the patient's desire to renounce the protocol. |
| **36. Procedures for withdrawing a patient from the study** |
| You will be informed in writing by means of a letter that it will no longer be part of the protocol, the cause will be explained, you will sign in agreement and your subsequent appointments will be scheduled according to the usual medical care of the institute. |
| **37. Criteria for premature (partial or complete) suspension of the study** |
| Significant loss of study patients. |
| **38. Selection criteria** |
| a) Inclusion criteria (It must include the definition of the age groups, sex and severity of the condition that will be allowed in the study) |
| 1. Comply with the informed consent procedure and sign the informed consent form. 2. Patients of legal age who have met the 2013 ACR Systemic Sclerosis criteria, the LeRoy-Medsger criteria, or the CREST syndrome criteria (with sclerodactyly and 2 of 4 of the other criteria (calcinosis, Raynaud's phenomenon, esophageal dysmotility, and telangiectasias). 3. Presence of at least one active digital ulcer at the time of inclusion in the study. The ulcer must have developed or worsened at least 30 days prior to study entry and must be located over or distal to the proximal interphalangeal joints.    1. An active ulcer is defined as a lesion on the finger with a depth discernible to the naked eye and with loss of continuity of the epithelium, which is associated with pain, and which is not due to other primary causes such as infection, arthritis, etc. This definition does not include fissures, paronychia, punctate scars, calcium extrusion, and indeterminate lesions (lesions in which denudation of tissue is not clearly observed and cannot be judged due to the presence of crust or necrotic tissue). 4. Stable vasodilator treatment in the 2 weeks prior to study inclusion. 5. Stable statin treatment in the 4 weeks prior to study enrollment. 6. Stable immunomodulatory or immunosuppressive treatment in the 4 weeks prior to study inclusion (stable dose of prednisone or its equivalent, cyclophosphamide, azathioprine, mycophenolic acid, D-penicillamine, methotrexate, chloroquine, hydroxychloroquine, leflunomide, or any other immunosuppressant). 7. Women of childbearing potential should use 1 reliable method of contraception.    1. Women of childbearing age with a negative pre-treatment pregnancy test and who consistently and correctly use (from the screening visit and up to 30 days after the end of the study) 1 reliable method of contraception may be included. Reliable methods of contraception include intrauterine devices, bilateral tubal occlusion, hormonal methods (combined or progesterone-only oral contraceptives, transdermal patches, vaginal rings, injections, and implants), and barrier methods (condom, diaphragm, or vaginal cap). Partner vasectomy and abstinence still require additional contraception.    2. A woman is considered infertile if she meets one or more of the following criteria:       1. Previous bilateral salpingo-oophorectomy, bilateral tubal occlusion or hysterectomy.       2. Premature ovarian failure confirmed by a specialist.       3. Genetic syndromes: Turner, XY genotype, uterine agenesis.       4. Age greater than 50 years, without treatment with hormone replacement therapy for the 2 years prior to the screening visit, with amenorrhea for at least 24 consecutive months prior to the screening visit. FSH measurement greater than 40 IU/L can be used as a postmenopausal equivalent. |
| b) Exclusion criteria |
| 1. Digital ulcers secondary to a condition other than SS 2. Comorbidities that could seriously affect the evaluation of hand function. 3. Women who are pregnant, lactating, or planning to become pregnant during the course of the study. 4. Abuse or dependence on alcohol and/or other substances in the 12 months prior to the screening visit 5. Patients with vasodilator treatment (such as calcium channel antagonists, ACE inhibitors, nitroglycerin, alpha-adrenergic blockers, angiotensin II receptor antagonists, 5-phosphodiesterase inhibitors, endothelin receptor inhibitors, prostanoids), N-acetylcysteine, antiplatelet therapy, conventional heparin or low molecular weight, who have received this treatment for less than 2 weeks prior to the screening visit or whose treatment has not been stable for this period. 6. Immunomodulatory treatment (such as prednisone or its equivalent, cyclophosphamide, azathioprine, mycophenolic acid, D-penicillamine, methotrexate, chloroquine, hydroxychloroquine, leflunomide or any other immunosuppressant) that has been started within 3 months prior to the screening visit or whose treatment has not been stable for at least 1 month prior to the screening visit. 7. Digital ulcers secondary to infected ES. 8. Use of topical growth factors or hyperbaric oxygen concomitant to the study. 9. Local injection of botulinum toxin during the study or up to 4 weeks prior to the baseline visit. 10. Upper extremity surgical sympathectomy or surgical wound debridement within one month prior to baseline visit. 11. Concomitant treatment with another experimental drug within 4 weeks prior to the study or during the study 12. Body mass index <18 13. Any condition that prevents the patient from attending protocol visits or understanding the nature of the protocol. 14. Diagnosis of active osteomyelitis with or without local or systemic treatment. 15. History of cancer in the family (parents/siblings/children). 16. Presence of cancer. 17. Presence of diabetes mellitus. 18. Presence of infection by hepatitis B, C or human immunodeficiency virus. |
| c) Elimination criteria |
| Patient's desire to renounce the protocol. |
| **39. Outcomes and variables** |
| **a)** **Main variables/outcomes to be measured.**   - The main outcome variable will be the presence of adverse events.   **b) Secondary variables/outcomes to be measured.**   1. Demographic variables will be recorded, as well as the clinical subtype of the disease and condition of internal organs according to the Medsger severity scale [^63^](#1302m92) . 2. Characteristics of the ulcers: The following characteristics will be evaluated immediately before the proposed treatment; after treatment these will be evaluated every week for the first four weeks, and then monthly for up to 6 months.    1. Number of ulcers.    2. Location. This variable will be recorded on a record sheet with a drawing of a hand.    3. Dimensions of digital ulcers in clinical form. The dimensions will be taken in the major axis and minor axis and will be expressed in millimeters. To evaluate the primary outcome, the largest and deepest ulcer will be taken into account, which will be designated as the index ulcer.    4. injured tissues. It will be recorded if the skin, subcutaneous tissue, tendons, joints and/or bone are affected.    5. Clinical photographs in dorsal and palmar projection. 3. Pain. An evaluation based on a visual analogue scale will be carried out before treatment, immediately after treatment, every week for the first four weeks, and then monthly for up to 6 months. 4. Duration, intensity and frequency of Raynaud Phenomenon. The frequency will be registered as the number of events per week, the duration will be registered in minutes and intensity will be registered as pale, cyanosis and hyperemic. This variable will be registered every 28 days. 5. Digital perfusion. The transcutaneous oximetry value will be recorded on each finger with a portable oximeter. You will have a record immediately before treatment, immediately after treatment, one hour after treatment; and then every week for the first four weeks, and then monthly for up to 6 months. 6. Nail bed vascularity with a video capillaroscope. It will be evaluated at the visit on day 0, before the administration of the treatment and on day 168. It will be classified according to the capillaroscopic pattern in early, active or late pattern and the number of capillaries will be counted per field. [^64^](#3mzq4wv) 7. Hand functionality.    1. The SHAQ (appendix 1) and COCHIN (appendix 2) questionnaires will be completed, similarly before treatment, and at week, month and 6 months. 8. Improvement in quality of life.    1. The evaluation will be carried out using the analogous pre-treatment questionnaire SF-36, and at week, month and 6 months. 9. Immunomodulatory effect of treatment: Percentage change of Th1, Th2, Th17 and Treg cells in peripheral venous blood between day 0 and 84. Inflation marker and cytokine levels in serum at day 0 and 84. |
| **40. Methods that will be used to contact patients** |
| 1. Medical appointments. 2. Phone calls. |
| **41. Statistical analysis (Description of the information processing and presentation plan. Include the justification of the statistical tests that will be used)** |
| A database will be created with the demographic variables and the clinical characteristics of the disease and the ulcer, in SPSS version 18. The Mann- Whitney U test will be used to compare the mean healing time of the index ulcer and other numerical paramethers between the 2 treatment groups. The proportions will be compared with the Chi square test and Fisher's exact if needed. |
| **42. Sample size justification (include the power of the study and the p-value that will be considered significant)** |
| This is a pilot study to evaluate the safety of ADSVF administration in digital ulcers in patients with systemic sclerosis. There are no previous studies that make it possible to adequately calculate the sample size, so the results of this study will provide information to be able to calculate the sample size for a study that will evaluate the efficacy of ADSVF on the healing time of digital ulcers in SSc patients. |
| **43. Recruitment potential (number of subjects to be recruited)** |
| Twenty |
| **44. In case of being multicenter, include the global number and the local number of the sample** |
| Does not apply. |
| **45. Procedures for reporting deviations from the original statistical plan** |
| Does not apply. |
| **46. Possible discomfort resulting from the study** |
| - 1. Pain and inflammation data in the liposuction area that will limit your daily activities for 3 days.   2. Pain in the area of infiltration of the lipo-graft enriched with Stromal Vascular Fraction. |
| **47. Potential risks** |
| 1. Surgical site infection. 2. Infection in the area of infiltration of the fat graft enriched with the stromal vascular fraction of the adipose tissue. 3. Exacerbation of tissue ischemia in the treated hand or fingers. |
| **48. Anticipated risk detection methods** |
| The clinical history of the patients who are considered to participate in the protocol will be studied. |
| **49. Safety measures for timely diagnosis and risk prevention** |
| 1. Telephone communication with the medical team to report symptoms or alarm signs (pain, hyperemia and/or redness at the liposuction site; pain, hyperemia and/or redness in the hands; fever; hypothermia and/or digital pallor in the hand). 2. To avoid infection in the surgical site, as prophylaxis, an IV dose of Amoxicillin/ Sulbactam 1 gr/500 mg IV will be applied or, in case of being allergic, Ciprofloxacin at a dose of 400 mg IV DU. |
| **50. Procedures to follow to resolve risks in case they arise** |
| 1. The patient will be informed of the clinical data of the infection and ischemia alarm, telling them to come within the next 24 hours for a consultation with one of the surgeons participating in the protocol. In that case, you will be given a free consultation and the necessary medications will be prescribed. 2. Antibiotics 3. Analgesics. 4. Wound care. 5. Application of Hyperbaric Medicine. |
| **51. Expected direct benefits** |
| 1. Decreased digital ulcers healing time. 2. Decreased pain secondary to the presence of the digital ulcers. 3. Improvement in the functionality of the hand. 4. Improvement in quality of life. |
| **52. Expected indirect benefits** |
| Close monitoring by a multidisciplinary team with experience in the pathology of these patients. |
| **53. Overall weighting of risks against benefits of the proposed study** |
| The risks are foreseeable with the correct selection of the patients participating in the study and with the application of antibiotics. The appearance of the most important risk is digital ischemia, which would be treated with Hyperbaric Medicine. Therefore, the predictability of complications and their successful management, versus decreased ulcer healing, justifies testing this treatment modality. |
| **54. Specify costs (direct/indirect, monetary, participation time, visits/transfers) that the research generates for the study subjects** |
| Subjects must attend 10 consultations of approximately 1 hour each, except for visit 0, which will require approximately 4 hours. Subjects will not pay for any of the procedures, treatments or consultations. |
| **55. Specify whether the consultations, laboratory/office exams, and medical/surgical treatments generated as a result of the study will or will not be covered by the patient/research subject** |
| No procedure, treatment or consultation will be paid by the patient. |
| **56. Inform who will cover the costs associated with the investigation** |
| 1. INCMNSZ Department of Surgery. Own funds of Department of Surgery. They will provide the necessary supplies before, during and after the adipose tissue extraction procedure and the stem cell-enriched fat grafting procedure, including medications and pre- and post-surgical care.  2. Department of Immunology and Rheumatology of INCMNSZ. Own funds from Dr. Tatiana Rodríguez for taking and processing blood samples to evaluate subpopulations of T lymphocytes and levels of inflammatory markers and cytokines.  3. INCMNSZ Department of Biochemistry. Own funds from Dr. Alejandro Zentella for reagents for enrichment of the lipograft with stem cells.  4. Department of Physiology of Nutrition of INCMNSZ. Own funds from Dr. Armando Tovar for reagents for enrichment of the lipograft with stem cells. |
| **57. If applicable, specify the incentives that will be offered (an incentive is understood as an offer or influence that compels us to carry out an action without implying a significant deviation from our general life plan; for example: giving a book for having participated)**  **Note: Compensation/incentive out of proportion is considered coercive.** |
| Does not apply. |
| **58. Bibliographic citations.** |
| **Bibliography**   1. Amanzi L, Braschi F, Fiori G, Galluccio F, Miniati I, Guiducci S, et al. Digital ulcers in scleroderma: staging, characteristics and sub-setting through observation of 1614 digital lesions. *Rheumatology (Oxford)*. 2010 Jul;49(7):1374–82. 2. Galluccio F, Matucci-Cerinic M. Two faces of the same coin: Raynaud phenomenon and digital ulcers in systemic sclerosis. *Autoimmun Rev*. Elsevier B.V.; 2011 Mar;10(5):241–3. 3. Botzoris V, Drosos A. Management of Raynaud’s phenomenon and digital ulcers in systemic sclerosis. *Joint Bone Spine*. 2011 Jul;78(4):341–6. 4. Nitsche A. Raynaud, digital ulcers and calcinosis in scleroderma. *Reumatol Clin*. SEGO; 2012;8(5):270–7. 5. Daumas a, Eraud J, Hautier a, Sabatier F, Magalon G, Granel B. Interests and potentials of adipose tissue in scleroderma. *Rev Med Interne*. Elsevier Masson SAS; 2013 Dec;34(12):763–9. 6. Oh C-K, Lee J, Jang B-S, Kang Y-S, Bae Y-C, Kwon K-S, et al. Treatment of atrophies secondary to trilinear scleroderma en coup de sabre by autologous tissue cocktail injection. *Dermatol Surg*. 2003 Oct;29(10):1073–5. 7. Consorti G, Tieghi R, Clauser LC. Frontal linear scleroderma: long-term result in volumetric restoration of the fronto-orbital area by structural fat grafting. *J* *Craniofac Surg*. 2012 May;23(3): e263–5. 8. Bank J, Fuller SM, Henry GI, Zachary LS. Fat grafting to the hand in patients with Raynaud phenomenon: a novel therapeutic modality. *Plast Reconstr Surg*. 2014 May;133(5):1109–18. 9. Karaaltin MV, Akpinar AC, Baghaki S, Akpinar F. Treatment of “en coup de sabre” deformity with adipose-derived regenerative cell-enriched fat graft*. J Craniofac Surg*. 2012 Mar;23(2): e103–5. 10. Guiducci S, Porta F, Saccardi R, Guidi S, Ibba-Manneschi L, Manetti M, et al. Autologous mesenchymal stem cells foster revascularization of ischemic limbs in systemic sclerosis: a case report. *Annals of internal medicine*. 2010. p. 650–4. 11. Salibian A, Widgerow AD, Abrouk M, Evans GR. Stem cells in plastic surgery: a review of current clinical and translational applications*. Arch Plast Surg*. 2013; 40:666–75 12. Kumar, V. et al., 2010. *Robbins & Cotran Pathologic Basis of Disease*. 13. TrojahnKølle S-F, Oliveri RS, Glovinski P viktor, Elberg JJ, Fischer-Nielsen A, Drzewiecki KT. Importance of mesenchymal stem cells in autologous fat grafting: A systematic review of existing studies. *J PlastSurg Hand Surg*. 2012; 46:59–68. 14. Friedenstein AJ, Petrakova KV, Kurolesova AI, Frolova GP. Heterotopic of bone marrow. Analysis of precursor cells for osteogenic and hematopoietic tissues. *Transplantation*.1968; 6:230–47. 15. López-Iglesias, P. et al., 2011. Short- and long-term fate of human AMSC subcutaneously injected in mice. *World journal of stem cells*, 3(6), pp.53–62. 16. Toyserkani NM, Christensen ML, Sheikh SP, Sørensen JA. Adipose-Derived Stem Cells: New Treatment for Wound Healing? *Ann Plast Surg*. 2014;00. 17. Zhu M, et al. Manual isolation of adipose-derived stem cells from human lipoaspirates. *Journal of Visualized Expermients*. 2013; 79:1-10. 18. Owen M, Friedenstein AJ. Stromal stem cells: marrowderivedosteogenic precursors. *Ciba Found Symp.*1988; 136:42–60. 19. Taupin, P., 2006. Adult neural stem cells, neurogenic niches, and cellular therapy. *Stem cell reviews*, 2, pp.213–219. 20. Watt, F.M., Lo Celso,C. & Silva-Vargas, V. Epidermal stem cells: an update. *Current opinion in genetics & development*.2006;16:518-524 21. Yen, T.H. & Wright, N.A. The gastrointestinal tract stem cell niche. *Stem Cell Rev*. 2006; 2:203-212. 22. Daniels, J.T., Harris, A.R. & Mason, C., 2006. Corneal epithelial stem cells in health and disease. *Stem cell reviews*, 2, pp.247–254. 23. Zuk PA, Zhu M, Mizuno H, Huang J, Futrell JW, Katz AJ, et al. Multilineage cells from human adipose tissue: implications for cell-based therapies. *TissueEng*. 2001;7:211–28. 24. Francis MP, Sachs PC, Elmore LW, Holt E. Isolating adipose-derived mesenchymal stem cells from lipoaspirateblood and saline fraction. *Organogenesis*. 2010; 6:11–14. 25. BarretJP, Sarobe N, Grande N, Vila D, Palacin JM. Maximizing results for lipofilling in facial reconstruction. *ClinPlastSurg* 2009; 36:487–92. 26. Tabit CJ, Slack GC, Fan K, Wan DC, Bradley JP. Fat grafting versus adipose-derived stem cell therapy: Distinguishing indications, techniques, and outcomes. *Aesthetic Plast Surg.* 2012;36: 704–13. 27. Gir P, Oni G, Brown S, Mojallal A, Rohrich RJ. Human adipose stem cells: current clinical applications. *PlastReconstr Surg*. 2012; 129:1277–90. 28. Fang B, Song YP, Li N, Li J, Han Q, Zhao RC. Resolution of refractory chronic autoimmune thrombocytopenic purpurafollowing mesenchymal stem cell transplantation: A case report. *Transplant Proc.* 2009; 41:1827–1830. 29. RaJC, Shin IS, Kim SH, Kang SK, Kang BC, Lee HY, et al. Safety of intravenous infusion of human adipose tissue-derived mesenchymal stem cells in animals and humans. *Stem Cells Dev*. 2011; 20:1297–308. 30. Yoshimura K, Sato K, Aoi N, Kurita M, Inoue K, Suga H, et al. Cell-assisted lipotransfer for facial lipoatrophy: Efficacy of clinical use of adipose-derived stem cells. *Dermatologic Surg*. 2008; 34:1178–85. 31. TiryakiT, Findikli N, Tiryaki D. Staged stem cell-enriched tissue (SET) injections for soft tissue augmentation in hostile recipient areas: A preliminary report. *Aesthetic Plast Surg*. 2011; 35:965–71. 32. Castro-Govea, Y. et al., 2012. Cell-assisted lipotransfer for the treatment of parry-romberg syndrome. *Archives of plastic surgery*, 39(6), pp.659–62. 33. Yoshimura K, Sato K, Aoi N, Kurita M, Hirohi T, Harii K. Cell-assisted lipotransfer for cosmetic breast augmentation: Supportive use of adipose-derived stem/stromal cells. *Aesthetic Plast Surg*. 2008; 32:48–55. 34. Kamakura T, Ito K. Autologous cell-enriched fat grafting for breast augmentation. *Aesthetic Plast Surg*. 2011; 35:1022–1030. 35. Kim M, Kim I, Lee SK, Bang SI, Lim SY. Clinical trial of autologous differentiated adipocytes from stem cells derived from human adipose tissue*Dermatol Surg*. 2011; 37:750–9. 36. Hassan WU, Greiser U, Wang W. Role of adipose-derived stem cells in wound healing. *Wound Repair Regen*. 2014 May; 22(3):313–25. 37. NaeS, Bordeianu I, Stăncioiu AT, Antohi N. Human adipose-derived stem cells: definition, isolation, tissue-engineering applications. *Rom J MorpholEmbryol*. 2013; 54:919–24. 38. Matsuda K, Falkenberg KJ, Woods AA, Choi YS, Morrison WA, Dilley RJ. Adipose-Derived Stem Cells Promote Angiogenesis and Tissue Formation for In Vivo *Tissue Engineering. Tissue Eng Part A*. 2013; 19:1327–35. 39. BarbaM, Cicione C, Bernardini C, Michetti F, Lattanzi W. Adipose-derived mesenchymal cells for bone regereneration: state of the art. *Biomed Res Int*. 2013 Jan;2013:416391. 40. Wu L, Cai X, Zhang S, Karperien M, Lin Y. Regeneration of articular cartilage by adipose tissue derived mesenchymal stem cells: perspectives from stem cell biology and molecular medicine. *J Cell Physiol*. 2013 May;228(5):938–44. 41. Euler de Souza Lucena E, Guzen FP, Lopes de Paiva Cavalcanti JR, Galvão Barboza CA, Silva do Nascimento Júnior E, Cavalcante JDS. Experimental considerations concerning the use of stem cells and tissue engineering for facial nerve regeneration: a systematic review*. J Oral Maxillofac Surg*. 2014 May;72(5):1001–12. 42. NambuM, Kishimoto S, Nakamura S, Mizuno H, Yanagibayashi S, Yamamoto N, et al. Accelerated wound healing in healing-impaired db/db mice by autologous adipose tissue-derived stromal cells combined with atelocollagen matrix. *Ann Plast Surg*. 2009; 62:317–21. 43. Amos PJ, Kapur SK, Stapor PC, et al. Human adipose-derived stromal cellsaccelerate diabetic wound healing: impact of cell formulation and delivery.*Tissue Eng Part A*. 2010;16:1595Y1606. 44. Di Rocco G, Gentile A, Antonini A, Ceradini F, Wu JC, Capogrossi MC, et al. Enhanced healing of diabetic wounds by topical administration of adipose tissue-derived stromal cells overexpressing stromal-derived factor-1: biodistribution and engraftment analysis by bioluminescent imaging. *Stem Cells Int*. 2010; 2011:304562. 45. MaharlooeiMK, Bagheri M, Solhjou Z, Jahromi BM, Akrami M, Rohani L, et al. Adipose tissue derived mesenchymal stem cell (AD-MSC) promotes skin wound healing in diabetic rats. *Diabetes Res ClinPract*. 2011; 93:228–34. 46. Nie C, Yang D, Xu J, Si Z, Jin X, Zhang J. Locally administered adipose-derived stem cells accelerate wound healing through differentiation and vasculogenesis. *Cell Transplant*. 2011; 20:205–16. 47. Nie C, Zhang G, Yang D, et al. Targeted delivery of adipose-derived stem cells via acellular dermal matrix enhances wound repair in diabetic rats. *J Tissue EngRegen Med*. 2012. 48. Kim EK, Li G, Lee TJ, Hong JP. The effect of human adipose-derived stem cells on healing of ischemic wounds in a diabetic nude mouse model.*PlastReconstr Surg*. 2011;128:387–94. 49. EbrahimianTG, Pouzoulet F, Squiban C, et al. Cell therapy based on adipose tissue derived stromal cells promotes physiological and pathological wound healing. *ArteriosclerThrombVasc Biol*. 2009; 29:503Y510. 50. TsumanoT, Kawai K, Ishise H, Nishimoto S, Fukuda K, Fujiwara T, et al. A new mouse model of impaired wound healing after irradiation.*J PlastSurg Hand Surg*. 2013;47:83–8. 51. SteinbergJP, Hong SJ, Geringer MR, Galiano RD, Mustoe TA. Equivalent Effects of Topically-Delivered Adipose-Derived Stem Cells and Dermal Fibroblasts in the Ischemic Rabbit Ear Model for Chronic Wounds.*AesthetSurg J*. 2012;32:504–19. 52. Hadad I, Johnstone BH, Brabham JG. Development of a porcine delayed wound healing model and its use in testing a novel cell-based therapy. *Int J Radiat Oncol Biol Phys*. 2010; 78:888Y896. 53. Zhu M, Zhou Z, Chen Y, et al. Supplementation of fat grafts with adipose-derived regenerative cells improves long-term graft retention. *Ann Plast Surg.* 2010; 64:222–8. 54. LinYC, Grahovac T, Oh SJ, Ieraci M, Rubin JP, Marra KG. Evaluation of a multi-layer adipose-derived stem cell sheet in a full-thickness wound healing model*. ActaBiomater*. 2013; 9:5243–50. 55. CianfaraniF, Toietta G, Di Rocco G, Cesareo E, Zambruno G, Odorisio T. Diabetes impairs adipose tissue-derived stem cell function and efficiency in promoting wound healing. *Wound Repair Regen*. 2013; 21:545–53. 56. El-Ftesi S, Chang EI, Longaker MT, Gurtner GC. Aging and diabetes impair the neovascular potential of adipose-derived stromal cells. *PlastReconstr Surg*. 2009; 123:475–85. 57. Yoshimura, K. et al. Ectopic fibrogenesis induced by transplantation of adipose-derived progenitor cell suspension immediately after lipoinjection. *Transplantation*.2008;85:12:1868–9. 58. RussoFP, Alison MR, Bigger BW, Amofah E, Florou A, Amin F, et al. The Bone Marrow Functionally Contributes to Liver Fibrosis. Gastroenterology. 2006; 130:1807–21. 59. Yoshimura, K. et al. *In vivo* Manipulation of stem cells for adipose tissue repair/reconstruction. *Regenerative Medicine*.2011;6:33–41. 60. DonnenbergVS, Zimmerlin L, Rubin JP, Donnenberg AD. Regenerative therapy after cancer: what are the risks? *Tissue Eng Part B Rev*. 2010; 16:567–75. 61. Ley General de Salud. Estados Unidos Mexicanos. Diario Oficial de la Federación 7 de febrero de 1984. Última reforma publicada en el Diario Oficial de la Federación el 15 de enero del 2014. 62. Rodríguez-Reyna TS, Furuzawa J, Cabiedes J. et al. Th17 peripheral cells are increased in diffuse cutaneous systemic sclerosis compared with limited illness: a cross-sectional study. *Rheumatol Int*. 2012;32(9):2653-60. 63. Medsger TA Jr, Bombardieri S, Czirjak L, et al. Assessment of disease severity and prognosis. Clin Exp Rheumatol. 2003; 21 (Suppl 29): S60-S64. 64. Maricq HR, Spencer-Green G, LeRoy EC. Skin capillary abnormalities as indicators of organ involvement in scleroderma (systemic sclerosis), Raynaud’s syndrome and dermatomyositis. *Am J Med*. 1976; 61:862-70. 65. Fang B, Song YP, Liao LM, Han Q, Zhao RC. Treatment of severe therapy-resistant acute graft-versus-host disease with human adipose tissue-derived mesenchymal stem cells. *Bone Marrow Transplant*. 2006; 38:389–390. 66. Fang B, Song Y, Zhao RC, Han Q, Lin Q. Using human adipose tissue-derived mesenchymal stem cells as salvage therapy for hepatic graft-versus-host disease resembling acute hepatitis. *Transplant Proc*. 2007; 39:1710–1713. 67. Fang B, Song YP, Li N, Li J, Han Q, Zhao RC. Resolution of refractory chronic autoimmune thrombocytopenic purpura following mesenchymal stem cell transplantation: A case report. *Transplant Proc*. 2009; 41:1827–1830. 68. Fang B, Mai L, Li N, Song Y. Favorable response of chronic refractory immune thrombocytopenic purpura to mesenchymal stem cells. *Stem Cells Dev*. 2012; 21:497–502. 69. Fang B, Song Y, Li N, Li J, Han Q, Zhao RC. Mesenchymal stem cells for the treatment of refractory pure red cell aplasia after major ABO-incompatible hematopoietic stem cell transplantation. *Ann Hematol*. 2009; 88:261–266. 70. Trivedi HL, Vanikar AV, Thakker U, et al. Human adipose tissue-derived mesenchymal stem cells combined with hematopoietic stem cell transplantation synthesize insulin. *Transplant Proc*. 2008; 40:1135–1139. 71. Vanikar AV, Dave SD, Thakkar UG, Trivedi HL. Cotransplantation of adipose tissue-derived insulin-secreting mesenchymal stem cells and hematopoietic stem cells: A novel therapy for insulin-dependent diabetes mellitus. *Stem Cells Int*. 2010; 2010:582382. 72. Garcia-Olmo D, Herreros D, Pascual M, et al. Treatment of enterocutaneous fistula in Crohn’s Disease with adipose-derived stem cells: A comparison of protocols with and without cell expansion. *Int J Colorectal Dis*. 2009; 24:27–30. 73. Garcia-Olmo D, Garcia-Arranz M, Herreros D. Expanded adipose-derived stem cells for the treatment of complex perianal fistula including Crohn’s disease. *Expert Opin Biol Ther*. 2008; 8:1417–1423. 74. Garcia-Olmo D, Herreros D, Pascual I, et al. Expanded adipose- derived stem cells for the treatment of complex perianal fistula: A phase II clinical trial. *Dis Colon Rectum.* 2009; 52:79–86. 75. Alvarez PD, Garcı´a-Arranz M, Georgiev-Hristov T, Garcı´a- Olmo D. A new bronchoscopic treatment of tracheomediastinal fistula using autologous adipose-derived stem cells. *Thorax* 2008; 63:374–376. 76. Ichim TE, Harman RJ, Min WP, et al. Autologous stromal vascular fraction cells: A tool for facilitating tolerance in rheumatic disease. *Cell Immunol*. 2010; 264:7–17. 77. Riordan NH, Ichim TE, Min WP, et al. Non-expanded adipose stromal vascular fraction cell therapy for multiple sclerosis. *J Transl Med*. 2009; 7:29. 78. Mesimäki K, Lindroos B, Törnwall J, et al. Novel maxillary reconstruction with ectopic bone formation by GMP adipose stem cells. *Int J Oral Maxillofac Surg*. 2009; 38:201–209. 79. Taylor JA. Bilateral orbitozygomatic reconstruction with tissue- engineered bone. *J Craniofac Surg*. 2010; 21:1612–1614. 80. Lendeckel S, Jödicke A, Christophis P, et al. Autologous stem cells (adipose) and fibrin glue used to treat widespread traumatic calvarial defects: Case report*. J Craniomaxillofac Surg*. 2004; 32:370–373. 81. Pak J. Regeneration of human bones in hip osteonecrosis and human cartilage in knee osteoarthritis with autologous adipose-tissue-derived stem cells: A case series. *J Med Case Reports* 2011; 5:296. 82. Yamamoto T, Gotoh M, Hattori R, et al. Periurethral injection of autologous adipose-derived stem cells for the treatment of stress urinary incontinence in patients undergoing radical prostatectomy: Report of two initial cases. *Int J Urol*. 2010; 17:75–82. 83. Van Laar JM, Farge D, Sont JK et al. Autologous hematopoietic stem cell transplantation vs intravenous pulse cyclophosphamide in diffuse cutaneous systemic sclerosis: a randomized clinical trial. JAMA 2014; 311(24):2490-8. |

| **Table 1. Expression Profile of Human Adipose Tissue-Derived Stem Cells on the Cell Surface** | | |
| --- | --- | --- |
|  | Expression Profile | |
|  | Positive expression | Negative expression |
|  | CD9, **CD10, CD13, CD29, CD34, CD44**, CD49a, **CD49d,** CD49e  CD51, CD54, CD55, CD59, CD61, CD63, CD71, CD73, CD90, CD105, CD138, CD140a, CD146, CD166, HLA-ABC, STRO-1 | **CD11a, CD11b, CD11c, CD14,** CD16, CD18, **CD31,** CD41a, CD49f, **CD45,** CD50, CD56,  CD62e, CD62l, CD62P, CD104, **CD106**, CD133, **CD144, CD146, HLA-DR, SMA,** ABCG2 |
| **Proposed ASC phenotype**  **(Common between 2 or more studies)** | **CD10, CD13, CD29, CD34, CD44, CD49d, CD54, CD90, CD140a**    **HLA-ABC, STRO-1** | **CD11a, CD11b, CD11c, CD14, CD31, CD45, CD106, CD144** |
| **Controversial markers in ASC** | CD105, CD117, CD140b, CD146, CD166, SMA, HLA-DR | |
| **Stromal cell markers** | CD29, CD44, CD73, CD90, CD166 | |
| **Hematopoietic markers** | CD31, CD34, CD45, ABCG2 | |
| **Common markers between 2 or more studies marked in bold.**  **SMA, smooth muscle actin; HLA, human leukocyte antigen; ABCG2, multidrug transporter protein G2**  **Taken from: Zuk P. et al. Multilineage cells from human adipose tissue: implications for cell-based therapies. TissueEng. 2001; 7:211–28.** | | |

| **Table 2. Clinical applications of stem cells derived from adipose tissue in other specialties** | | | |
| --- | --- | --- | --- |
| **Specialties** | **References** | **No. of patients treated** | **CTDTA Dosage and Administration** |
| **Hematologic and immunologic disorders** | Fang et al. | 14 | 1-2x10 6 allogeneic ASC/kg, IV |
| **Diabetes mellitus** | Trivedi et al. | 5 | 3.15x10 6 allogeneic ASC injected by infusion  intraportal under general anesthesia using  minilaparotomy |
| **Digestive disorders** | Vanikar et al.  Garcia-Olmo et al. | 11  63 | 3x10 6 to 2x10 7 autologous ASC within the fistula |
| **Autoimmune diseases** | Ichim et al.  Riordan et al. | 1  3 | 53x10 6 autologous ADSVF in two IV infusions  25-75x10 6 autologous ADSVF IV |
| **Tracheal-mediastinal fistula** | Alvarez et al. | 1 | 4.9x10 6 autologous ADSVF within the cavity of the  fistula |
| **Bone tissue repair** | Lendeckel et al.  Medimäki et al.  Taylor  Pak | 1  1  1  4 | 295x10 6 autologous ADSVF  13x10 6 autologous ASC  28 ml of solid fraction of lipoaspirate fresh autologous  10 cm 3 autologous ADSVF |
| **Urological disorders** | Yamamoto et al. | 2 | 2.4-3.2x10 7 autologous ADSVF within the urethral sphincter |
| **Neurological disease** | Ra et al. | 8 | 4x10 8 autologous ASC IV |
| **Total** |  | 115 |  |
| **ASC, Stem cells derived from adipose tissue; IV, Intravenous; ADSVF, Stromal vascular portion of adipose tissue.**  **Taken from: Gir P, et al. Human adipose stem cells: current clinical applications. Plast. Reconstr. Surg. 2012; 129:1277–90.** | | | |

| **Table 3. Clinical Applications of Stem Cells Derived from Adipose Tissue in Plastic Surgery** | | |
| --- | --- | --- |
| **References** | **No. of patients treated** | **ASC Dosage and Administration** |
| **Soft tissue augmentation** |  |  |
| **Yoshimura et al.**  **Yoshimura et al.**  **Yoshimura et al.**  **Tiryaki et al.**  **Kamakura and Ito**  **Kim et al.** | 15  40  29  20  31 | 263.5 mL of ADSVF-enriched fat injected into each breast.  272.7 mL of ADSVF-enriched fat injected into each breast.  133 ml of injected fat in the non-ACFT group, 100 ml of ADSVF-enriched fat in the TGCA group  10-390ml of grease enriched with ADSVF (TGCA) by local admin  240ml of ADSVF-enriched fat in each breast (TGCA)  0.11-4.63x10 7 autologous ASC in each scar |
| **Cicatrization** |  |  |
| **Rigotti et al.**  **Akita et al.** | 20  1 | 7.4±3.6x10 5 autologous ADSVF in each lesion (60-80ml of fatty tissue)  3.8x10 7 autologous ADSVF within each lesion |
| **Tissue engineering** |  |  |
| **Stillaert et al.** | 12 | 0.67-1.4x10 6 ASC per scaffold |
| **Total** | 174 |  |
| **ASC, adipose tissue-derived stem cells; ADSVF, stromal vascular portion of adipose tissue; ACFT, assisted cellular fat transplantation.**  **Taken from: Gir P, et al. Human adipose stem cells: current clinical applications. Plast. Reconstr. Surg. 2012 ;129: 1277–90.** | | |
