# Supplementary material for: Adipose derived stromal vascular fraction and fat graft for treating the hands of patients with systemic sclerosis. A randomized clinical trial
Source: PLoS One. 2023 Aug 14;18(8):e0289594. doi: 10.1371/journal.pone.0289594 (PMC10424873; doi:10.1371/journal.pone.0289594)
Supplement: S1 Table — (PDF) [file pone.0289594.s004.pdf]

Patient medications

Patient	Immunosuppressant	Steroid	Other
**Control 1**	Mycophenolate		Sildenafil, omeprazole, laxoya
**Control 2**			Pregabalin, propranolol, tramacet, spironolactone, lactulax
**Control 3**	Tocilizumab	Prednisone	Pantoprazole, seretide, calcium, fluoxetine, prolia
**Control 4**	Colchicine		Omeprazole, calcium, losartan, vitamin D, carvedilol
**Control 5**			Pantoprazole, cinitapride, cilostazol, aspirin
**Control 6**	Azathioprine		Rivaroxaban
**Control 7**	Azathioprine		Spironolactone, pentoxifylline, levothyroxine, omeprazole, seretide, calcium, vitamin D
**Control 8**	Methotrexate	Prednisone	Enalapril, omeprazole, calcium, tradol
**Control 9**	Mycophenolate		Omeprazole, tramadol, paracetamol, calcium, vitamin B12
**Control 10**			Nifedipine, atorvastatin, calcium, vitamin D, ursodeoxycholic acid, cholestyramine
**Experimental 1**	Colchicine		Omeprazole
**Experimental 2**	Methotrexate		Omeprazole, metoprolol, ciprofloxacin

**Experimental 3**	Mycophenolate		Omeprazole, aspirin, nifedipine, rosuvastatin, bromazepam fluoxetine
**Experimental 4**	Mycophenolate		Losartan
**Experimental 5**			Aspirin protect, clonazepam, sertraline
**Experimental 6**	Mycophenolate		Omeprazole
**Experimental 7**	Colchicine, mycophenolate, ursofalk		Omeprazole, bezafibrate, amlodipine
**Experimental 8**	Azulfidine	Prednisone	Meloxicam, enalapril, amitriptyline, amlodipine, furosemide
**Experimental 9**	Azatriprine		Omeprazole, raloxifene, vitamin D, citalopram
**Experimental 10**	Mycophenolate	Prednisone	Dexivant, metoprolol, rifaximin
