# Supplementary material for: Adipose derived stromal vascular fraction and fat graft for treating the hands of patients with systemic sclerosis. A randomized clinical trial
Source: PLoS One. 2023 Aug 14;18(8):e0289594. doi: 10.1371/journal.pone.0289594 (PMC10424873; doi:10.1371/journal.pone.0289594)
Supplement: S2 Table — (PDF) [file pone.0289594.s005.pdf]

Patient Comorbidities

Patient	Type of SSc	Comorbidities:	Finished the study
**Control 1**	dSSc		A
**Control 2**	dSSc	2,3,4,5	A
**Control 3**	dSSc	2,8,9	A
**Control 4**	dSSc	11	A
**Control 5**	LSSc	10,11	A
**Control 6**	dSSc	1,2,3,10	B
**Control 7**	LSSc	14	A
**Control 8**	dSSc	10	A
**Control 9**	LSSc		A
**Control 10**	LSSc	5,6	A
**Experimental 1**	dSSc		A
**Experimental 2**	dSSc	6	A
**Experimental 3**	dSSc		A
**Experimental 4**	dSSc	6,7	A
**Experimental 5**	LSSc		A
**Experimental 6**	dSSc		A
**Experimental 7**	dSSc	12	A
**Experimental 8**	dSSc	13	A
**Experimental 9**	LSSc		A
**Experimental 10**	dSSc		A

Key: LSSc. Limited systemic sclerosis, dSSc. Diffuse systemic sclerosis 1. Sx. Sjögren, 2. Diffuse interstitial lung disease, 3. Pulmonary arterial hypertension, 4. Barrett's esophagus, 5. Liver cirrhosis, 6. Systemic arterial hypertension, 7. Dyslipidemia, 8. Uterine myomatosis, 9. Major depressive disorder, 10. Gastroesophageal reflux disease, 11. CREST syndrome, 12. Osteoarthritis, 13. Rheumatoid arthritis, 14. Mixed connective tissue disease. A. Yes, B. No
