# Supplementary material for: Adipose derived stromal vascular fraction and fat graft for treating the hands of patients with systemic sclerosis. A randomized clinical trial
Source: PLoS One. 2023 Aug 14;18(8):e0289594. doi: 10.1371/journal.pone.0289594 (PMC10424873; doi:10.1371/journal.pone.0289594)
Supplement: S3 Table — (PDF) [file pone.0289594.s006.pdf]

Complete results control and experimental groups

Concept	Day 0	Day 168	P[1]	P[2]
**Total Digit Active Motion** (degrees)				
**Thumb**				
Control	80(54.2-94.7)	67.5(54.1-95.8)	0.72	0.68
Experimental	80(39.4,120)	77.5(42.5-103)	0.52	
**Index finger**				
Control	160(112,192)	170(106,185)	0.15	0.32
Experimental	105 (58.1,178)	110 (59.8,164)	0.61	
**Middle finger**				
Control	152(116,192)	170(117,207)	0.20	0.79
Experimental	130(78.0,198)	117(78.1,176)	0.67	
**Ring finger**				
Control	177(125,203)	185(117,207)	0.81	0.76
Experimental	145 (89.2,198)	137(86.6,195)	0.73	
**Little finger**				
Control	160(110,218)	175(109,220)	0.09	0.29
Experimental	180 (93.2,208)	127(73.3,185)	0.20	
**Digital Sp02 (% oxygen saturation)**				
**Thumb**				
Control	93.5(91.2,94.7)	97.0(93.5,97.5)	**0.05**	0.09
Experimental	94.0(93.0-96.1)	95.0 (93.0,96.5)	0.83	
**Index finger**				
Control	94.0(92.0,95.1)	96.0(93.0,97.8)	0.07	0.30
Experimental	92.0(85.8,94.7)	94.5(92.8,95.5)	0.09	
**Middle finger**				
Control	93.0 (88.1,97.1)	94.0(89.7,96.8)	0.75	0.73
Experimental	93.5 (89.7-96.2)	94.5(92.7,96.8)	0.44	
**Ring finger**				
Control	91.5(88.2,93.7)	94.0(92.2,96.0)	0.17	0.63
Experimental	91.0(85.5,95.5)	95.5(93.0,96.5)	**0.05**	
**Little finger**				
Control	94.0(91.3,95.6)	92.0 (88.7,95.0)	0.44	0.08
Experimental	93.5 (87.5,96.3)	95.0(93.7,96.2)	0.13	
**Thumb Opposition**				
Control	8.50(6.86,8.94)	9.00(6.93,9.29)	0.31	0.63
Experimental	7.00(5.16,8.04)	6.50(4.81,7.99)	0.58	
**Pain**				
Control	4.00(2.64,6.16)	2.00(1.51,6.04)	0.91	**0.02**
Experimental	5.00(3.08,6.12)	0.00(0.00,2.95) *	**0.006**	
**Raynaud Phenomenon**				
**Frequency, number of events per day/week**				
Control	5.50(2.68,13.1)	00(0.00,3.18)	**0.01**	0.418
Experimental	4.50(2.87,6.33)	0.50(0.10,1.10)	**0.005**	

**Raynaud Intensity**				
Control	2.00(1.22,2.18)	0.00(0.00,1.44)	**0.03**	0.60
Experimental	2.00(1.50,2.10)	0.50(0.10,1.10)	**0.006**	
**Duration in minutes in every event**				
Control	17.5(2.12,54.4)	0.00(0.00,10.3)	**0.02**	0.14
Experimental	12.5(6.55,35.6)	0.00(0.00,6.82)	**0.005**	
**Hand Function (Cochin)**				
Control	21.0(13.6,39.9)	23.0(11.8,40.6)	0.85	0.79
Experimental	21.5 (12.3,38.7)	23.5(12.5,36.5)	0.51	
**Health status and disability index (SHAQ)**				
Control	0.70(0.53-1.17)	0.70(0.41-0.95)	0.40	0.32
Experimental	1.05 (0.61-1.42)	0.67(0.36-0.90)	0.11	
**Quality of life (SF-36)**				
Control	40.0(28.5,46.4)	35.0(17.5-51.3)	0.55	0.15
Experimental	37.5 (32.1-57.8)	45.0(38.7-62.2)	**0.04**	
**Vascular density of the nail bed**				
Control	4.62(3.43,5.36)	4.65(3.70,5.27)	0.76	0.30
Experimental	4.31(3.46,6.60)	4.63(3.51,6.26)	0.33	
**Skin affection of the hand**				
Control	6.50(2.32,15.8)	5.50(1.42,12.9)	**0.05**	0.19
Experimental	15.0(6.84,17.7)	15.5(6.36,16.6)	0.07	

The sample size of the control group n=9, experimental group n=10; Data are presented as median (95% confidence intervals).

*P<0.05, U de Mann-Whitney analyze the differences between group

P^1 Analyze the differences between baseline and final was used Wilcoxon signed-rank test.

P^2 These data were log-transformed before statistical analyses was ANOVA for repeated measures to determine the time x group interaction.
