# Supplementary material for: Adipose derived stromal vascular fraction and fat graft for treating the hands of patients with systemic sclerosis. A randomized clinical trial
Source: PLoS One. 2023 Aug 14;18(8):e0289594. doi: 10.1371/journal.pone.0289594 (PMC10424873; doi:10.1371/journal.pone.0289594)
Supplement: Attachment — Submitted filename: LETTER 2 OF RESPONSES TO REVIEWERS.docx [file pone.0289594.s010.docx]

**LETTER 2 OF RESPONSES TO REVIEWERS**

Dear editor:

We are very happy to hear about the publisher's decision. We send the responses to the reviewers and hope to meet all your requirements.

Response to editor

Comment 1. Dear editor, we have written the manuscript according to the journal's instructions. However, as you have suggested, we have added to the end of the references a section called Figure Legends.

Comment 2. The p-value in the manuscript has been changed as you have suggested.

**Changes suggested.**

Comment 1. The figure 4 has been modified using box plots, and the statistical analysis was performed with Wilcoxon signed rank test.

Comment 2. The discussion has been shortened, and the references have been restructured.

**Response to reviewers**

**Reviewer 4.**

Comment 1. The discussion has been modified and shortened and the references have been restructured.

Comment 2. The p value in the manuscript has been modified according to your suggestions.

Comment 3. The figure 4 has been modified using box plots and the statistical analysis was performed with Wilcoxon signed rank test.

Comment 4. Thank you for your comment. We agree with your point of view. Due to an error in the initial capture of data on the number of ulcers per patient, we were unable to know how many initial and final ulcers there were in a patient. Therefore, in the discussion section it has been pointed out as a limitation as follows:

“Although the number of digital ulcers was statistically lower in the experimental group compared to the control group at the end of the study, these results must be interpreted with caution, since unfortunately we did not assess the number of digital ulcers existing at the beginning and at the end of the study in each patient.”
